# Supplementary material for: MARS an improved de novo peptide candidate selection method for non-canonical antigen target discovery in cancer
Source: Nat Commun. 2024 Jan 22;15:661. doi: 10.1038/s41467-023-44460-z (PMC10803737; doi:10.1038/s41467-023-44460-z)

SUPPLEMENTARY INFORMATION

Supplementary Table 1: Five-Fold Cross-Validations using Baseline ALC model, Two-Factor alpha model, Two-Factor beta model, and Three-Factor MARS model. Column 2-5 shows the optimal parameters learned by grid search for each part, suggesting the models are robust. Column 6-8 gives the results of a one-sided Paired Wilcoxon signed-rank tests on F-ranks of true peptide sequences; significant drop of F-ranks indicates improved identification power.

Optimal Parameter Values					P-Value of Paired Wilcoxon Tests		
Part	Two-Factor α Model	Two-Factor β Model	Three-Factor MARS		F-Rank(ALC) v F-Rank (Two-Factor β)	F-Rank(Two-Factor β) v F-Rank (Two-Factor α)	F-Rank(Two-Factor α) v F-Rank (Three-Factor MARS)
	α	β	α	β	F-Rank (Two-Factor β)	F-Rank (Two-Factor α)	F-Rank (Three-Factor MARS)
1	0.77	0.28	0.72	0.08	<2.2×10-16	<2.2×10-16	<2.2×10-16
2	0.76	0.27	0.73	0.07	<2.2×10-16	<2.2×10-16	<2.2×10-16
3	0.77	0.28	0.72	0.08	<2.2×10-16	<2.2×10-16	=3.5×10-13
4	0.77	0.28	0.69	0.09	<2.2×10-16	<2.2×10-16	=1.3×10-10
5	0.77	0.28	0.70	0.08	<2.2×10-16	<2.2×10-16	<2.2×10-16

Supplementary Table 2: Number of peptide sequences reported to bind to each respective HLA allele in the Wilhelm et al. dataset

HLA	Peptides	HLA	Peptides
**HLA-A01:01**	1547	**HLA-B35:01**	635
**HLA-A02:01**	3541	**HLA-B35:03**	465
**HLA-A02:02**	661	**HLA-B35:07**	467
**HLA-A02:03**	530	**HLA-B35:08**	138
**HLA-A02:04**	664	**HLA-B37:01**	163
**HLA-A02:05**	503	**HLA-B38:01**	203
**HLA-A02:06**	476	**HLA-B38:02**	210
**HLA-A02:07**	528	**HLA-B39:01**	244
**HLA-A02:11**	581	**HLA-B39:24**	51
**HLA-A03:01**	1604	**HLA-B40:01**	1236
**HLA-A11:01**	1860	**HLA-B40:02**	2083
**HLA-A11:02**	1008	**HLA-B40:06**	207
**HLA-A23:01**	664	**HLA-B41:01**	176
**HLA-A24:02**	1425	**HLA-B41:03**	20
**HLA-A24:06**	93	**HLA-B42:01**	892
**HLA-A24:07**	323	**HLA-B44:02**	1971
**HLA-A24:13**	87	**HLA-B44:03**	2487
**HLA-A25:01**	345	**HLA-B44:09**	59
**HLA-A26:01**	269	**HLA-B44:27**	119
**HLA-A26:08**	51	**HLA-B44:28**	58
**HLA-A29:02**	825	**HLA-B45:01**	282
**HLA-A30:01**	208	**HLA-B46:01**	211
**HLA-A30:02**	178	**HLA-B49:01**	449
**HLA-A31:01**	262	**HLA-B50:01**	188
**HLA-A32:01**	411	**HLA-B51:01**	460
**HLA-A33:01**	72	**HLA-B51:08**	52
**HLA-A33:03**	96	**HLA-B52:01**	76
**HLA-A34:01**	82	**HLA-B53:01**	339
**HLA-A34:02**	335	**HLA-B54:01**	58
**HLA-A36:01**	350	**HLA-B55:01**	144
**HLA-A66:01**	109	**HLA-B55:02**	113
**HLA-A68:01**	215	**HLA-B56:01**	140
**HLA-A68:02**	110	**HLA-B57:01**	442
**HLA-A69:01**	37	**HLA-B57:03**	256
**HLA-A74:01**	265	**HLA-B58:01**	226
**HLA-B07:02**	3180	**HLA-B58:02**	56
**HLA-B07:04**	442	**HLA-C01:02**	64
**HLA-B08:01**	1096	**HLA-C02:02**	329
**HLA-B13:01**	224	**HLA-C03:02**	150
**HLA-B13:02**	286	**HLA-C03:03**	334
**HLA-B14:02**	151	**HLA-C03:04**	391
**HLA-B15:01**	813	**HLA-C04:01**	488
**HLA-B15:02**	295	**HLA-C04:03**	129
**HLA-B15:03**	194	**HLA-C05:01**	594
**HLA-B15:10**	164	**HLA-C06:02**	281
**HLA-B15:11**	55	**HLA-C07:01**	159
**HLA-B15:17**	125	**HLA-C07:02**	323
**HLA-B18:01**	817	**HLA-C07:04**	232
**HLA-B18:03**	79	**HLA-C07:06**	17
**HLA-B27:01**	590	**HLA-C08:01**	182
**HLA-B27:02**	431	**HLA-C08:02**	270
**HLA-B27:03**	281	**HLA-C12:02**	769
**HLA-B27:04**	259	**HLA-C12:03**	360
**HLA-B27:05**	1705	**HLA-C14:02**	229
**HLA-B27:06**	205	**HLA-C14:03**	207
**HLA-B27:07**	336	**HLA-C15:02**	255
**HLA-B27:08**	322	**HLA-C16:01**	397
**HLA-B27:09**	655	**HLA-C17:01**	110

Supplementary Table 3: Overview of MARS peptide identifications in each sample for the associated spectra of the 508 identified non-canonical peptides in the Chong et al. dataset

	ME275	*ME290*	*0D5P*	*OMM745*	*0NVC*	*T1015A*	*T1185B*	*T2289*	*T2671*	*Peptides*
Peaks DeNovo	10	19	82	22	17	30	119	26	10	213
MARS	10	18	85	22	24	32	120	27	10	227
Total	17	24	149	45	33	47	169	49	20	508

Supplementary Table 4: Overview of MARS peptide identifications for each sample in the complete Chong et al. dataset

Sample ID	*Sample Types*	*# DB Peptides (netMHCpan ≤ 10)*	*# MARS Human Peptides*	*# MARS 1AA Substitution Peptides*	*# MARS Non-Canonical Peptides*	*# MARS Unmatched Peptides*
0NVC	Melanoma Cell-line	8106	1134	2763	1468	50322
0MM745	Melanoma Cell-line	11954	2922	3018	1708	34106
0D5P	Melanoma Cell-line	16594	4060	4905	3607	76978
Me275	Melanoma Cell-line	6991	1553	1568	946	9923
Me290	Melanoma Cell-line	7585	1237	1556	680	10358
T1015A	Melanoma Cell-line	10179	2325	1740	1050	20307
T1185B	Melanoma Cell-line	22145	12058	6764	4150	76701
C3N2671	Tumour/Control Tissues (Lung squamous cell carcinoma, grade 2)	12416	4370	3906	2104	21402
C3N2289	Tumour/Control Tissues (lung adenocarcinoma, G2)	13090	2441	2204	1333	20945
Total		83295	28232	26386	15966	310309

Supplementary Table 5: HLA-typing results for cervical tumour patients

SAMPLE	HLA-A1	HLA-A2	HLA-B1	HLA-B2	HLA-C1	HLA-C2
1	HLA-A*02:01	HLA-A*11:01	HLA-B*13:02	HLA-B*27:05	HLA-C*06:02	HLA-C*02:02
2	HLA-A*25:01	HLA-A*01:01	HLA-B*40:01	HLA-B*18:01	HLA-C*03:04	HLA-C*12:03
3	HLA-A*24:02	HLA-A*01:01	HLA-B*49:01	HLA-B*37:01	HLA-C*06:02	HLA-C*07:01
4	HLA-A*03:01	HLA-A*02:01	HLA-B*40:02	HLA-B*07:02	HLA-C*07:02	HLA-C*02:02
5	HLA-A*02:01	HLA-A*02:01	HLA-B*56:01	HLA-B*15:01	HLA-C*01:02	HLA-C*03:04
6	HLA-A*26:01	HLA-A*01:01	HLA-B*37:01	HLA-B*13:02	HLA-C*06:02	HLA-C*06:02
7	HLA-A*02:01	HLA-A*03:01	HLA-B*51:01	HLA-B*18:01	HLA-C*14:02	HLA-C*07:01
8	HLA-A*11:01	HLA-A*03:01	HLA-B*07:02	HLA-B*35:01	HLA-C*04:01	HLA-C*07:02
9	HLA-A*03:01	HLA-A*02:01	HLA-B*57:01	HLA-B*44:02	HLA-C*05:01	HLA-C*06:02
10	HLA-A*24:02	HLA-A*01:01	HLA-B*13:02	HLA-B*50:01	HLA-C*06:02	HLA-C*06:02

Supplementary Figure 1: I replacement by L maintains validity of NetMHCpan 4.1 prediction across distinct HLA alleles. (A) K-L divergences were computed as statistics to characterize the similarity of two distributions D(Original || Only I) and D(Original || Only L). KLDiv Boxplot shows that "Only L" had consistently very small K-L divergences, and a one-sided paired Wilcoxon signed rank test suggested that D(Original || Only I) is significantly greater than D(Original || Only L) with p = 0.0156. (B) Rank score distributions for example alleles for original peptide sequence, all I replaced by L (Only L), and vice versa (Only I). Peptide numbers included in this analysis: HLA-A*01:01: 1713; HLA-A*02:01: 5051; HLA-A*03:01: 6732; HLA-A*11:01: 10820; HLA-B*44:02: 6433; HLA-B*57:03: 8022; HLA-C*03:04: 2900.

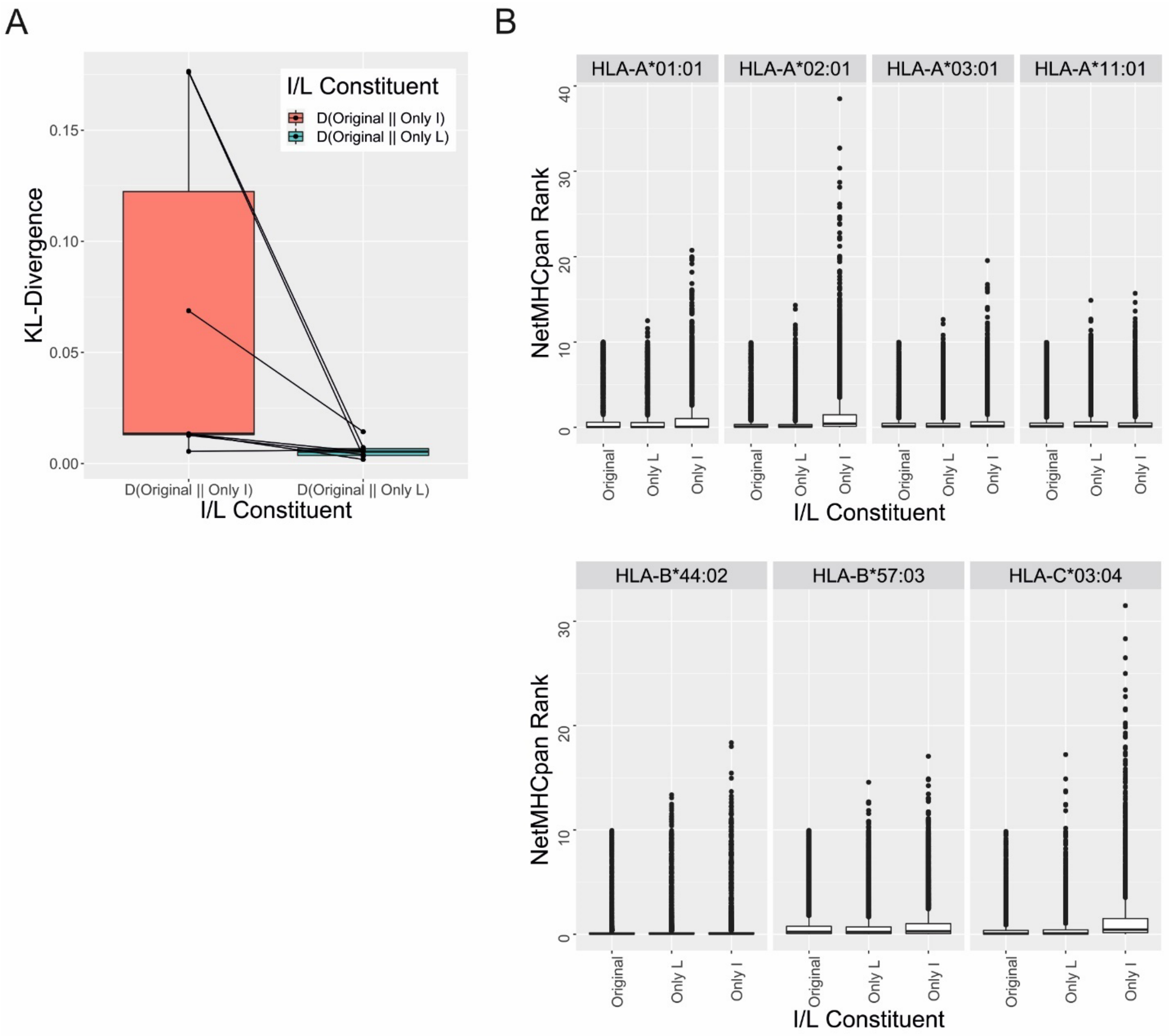

Supplementary Figure 2: HLA allele distributions for Chong et al. dataset, and cervical tumour patient cohort.

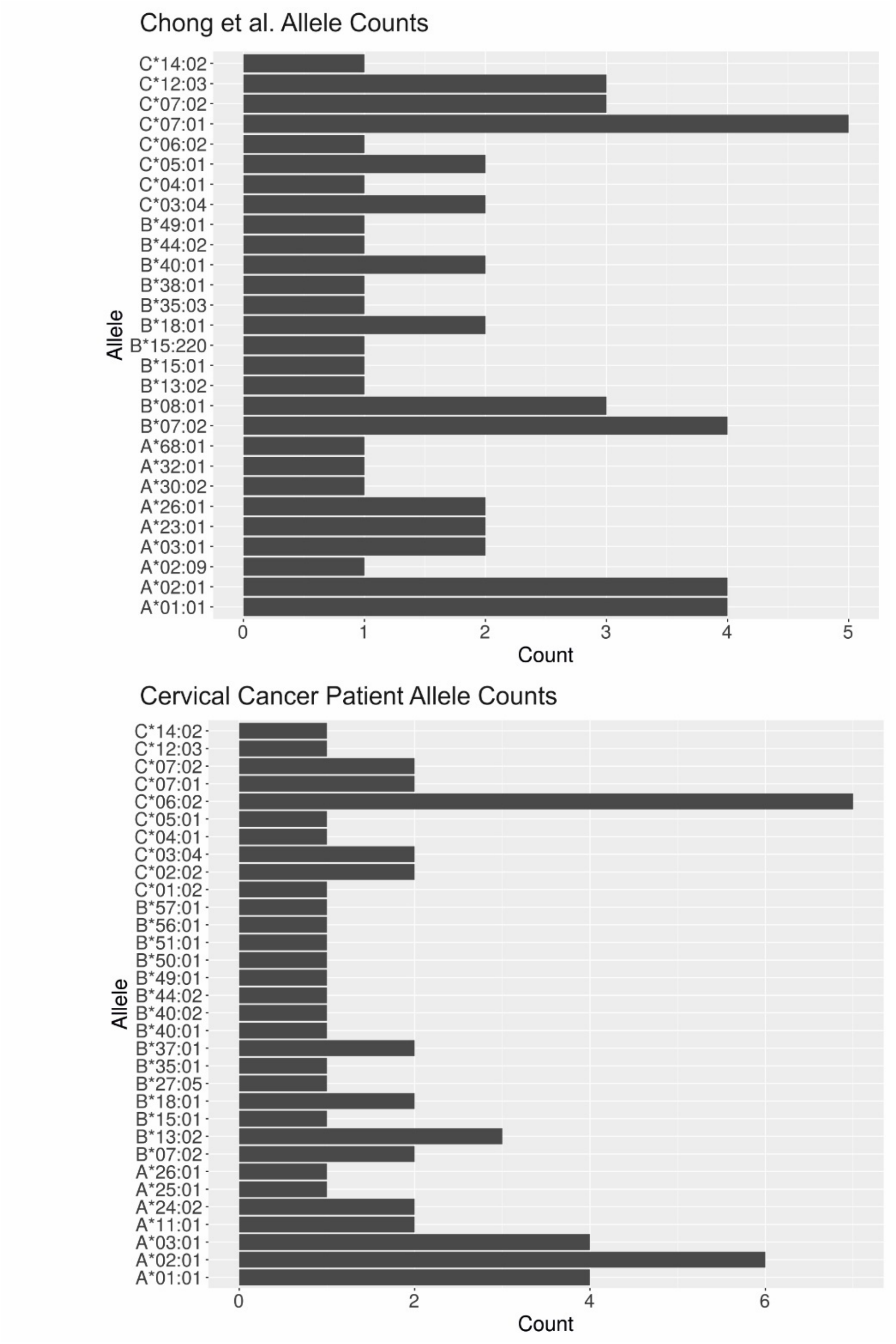

Supplementary Figure 3: Distribution of A-score (mean confidence, left panel) and MARS score for the discoverable (1904) and non-recoverable (370) peptide spectra for the Chong *et al.* dataset.

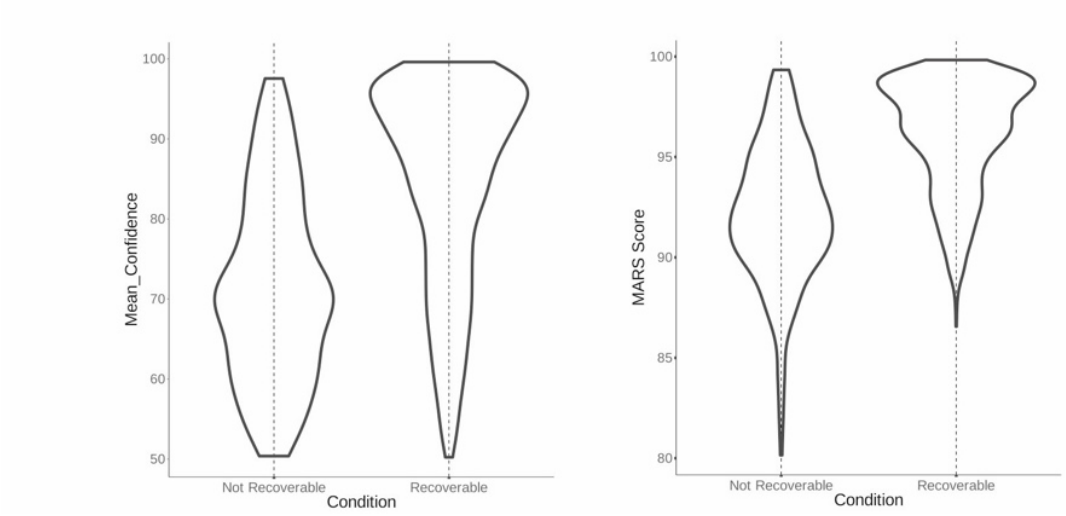

Supplementary Figure 4: Spectral matching analysis with the Universal Spectrum Explorer[59]. Top: experimental spectrum, bottom: spectrum derived from synthetic counterpart peptide.

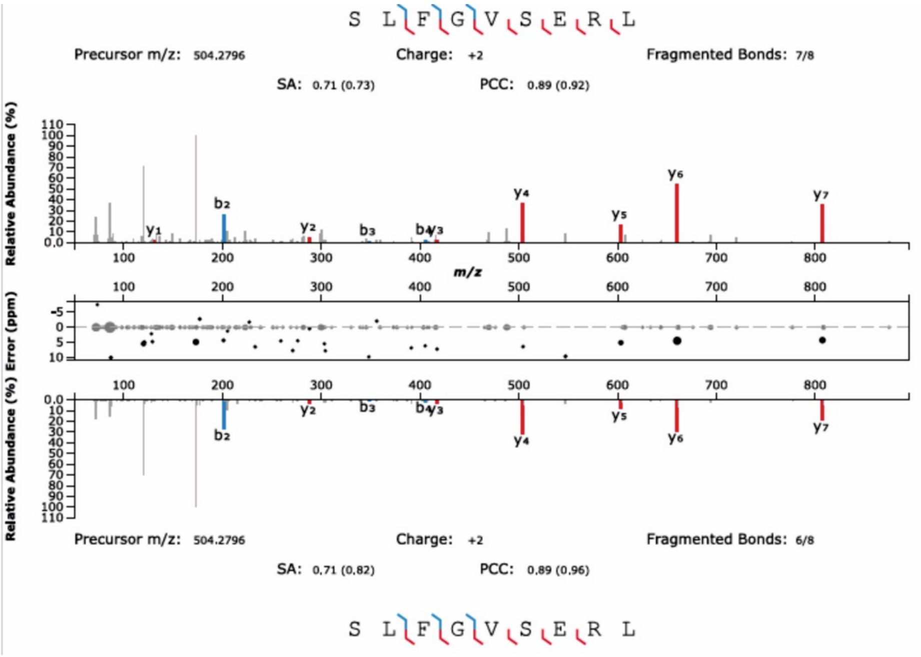

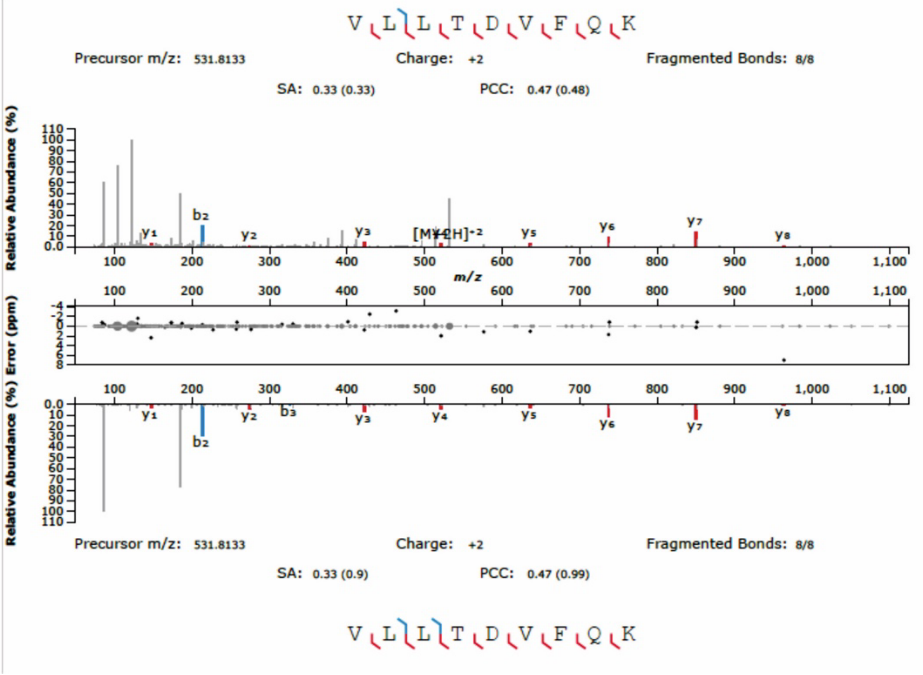

Supplementary Figure 5. Gating strategy for the identification of peptide-reactive CD8+ T cells. After gating on physical parameters and FSC-A/FSC-H doublet exclusion, live (Live/Dead Aqua−) CD3+ T cells were selected and the frequency of multimer+ or Dex+ CD8+ T cells out of total CD8+ T cells calculated.

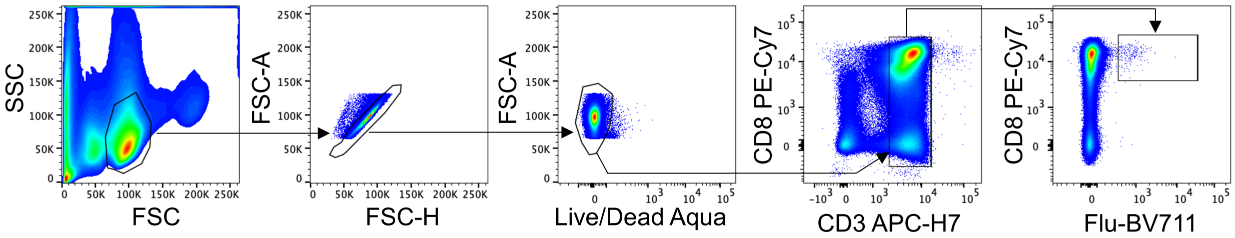

Supplement: Supplementary file 1 — Supplementary Information [file 41467_2023_44460_MOESM1_ESM.pdf]
