# Supplementary material for: MARS an improved de novo peptide candidate selection method for non-canonical antigen target discovery in cancer
Source: Nat Commun. 2024 Jan 22;15:661. doi: 10.1038/s41467-023-44460-z (PMC10803737; doi:10.1038/s41467-023-44460-z)
Supplement: Supplementary file 3 — Description of Additional Supplementary Files [file 41467_2023_44460_MOESM3_ESM.pdf]

Description of Additional Supplementary Files

Supplementary Data 1: Peptide validation for FDR estimation (107 peptides)

Supplementary Data 2: Spectral matching analysis with the Universal Spectrum Explorer for the 107 selected peptides for FDR estimation. Spectral comparison between experimental detected peptide spectra and the respective spectra originating from the relevant synthetic peptide counterpart
