# Supplementary material for: MARS an improved de novo peptide candidate selection method for non-canonical antigen target discovery in cancer
Source: Nat Commun. 2024 Jan 22;15:661. doi: 10.1038/s41467-023-44460-z (PMC10803737; doi:10.1038/s41467-023-44460-z)
Supplement: Supplementary file 5 — Supplementary Data 2 [file 41467_2023_44460_MOESM5_ESM.pdf]

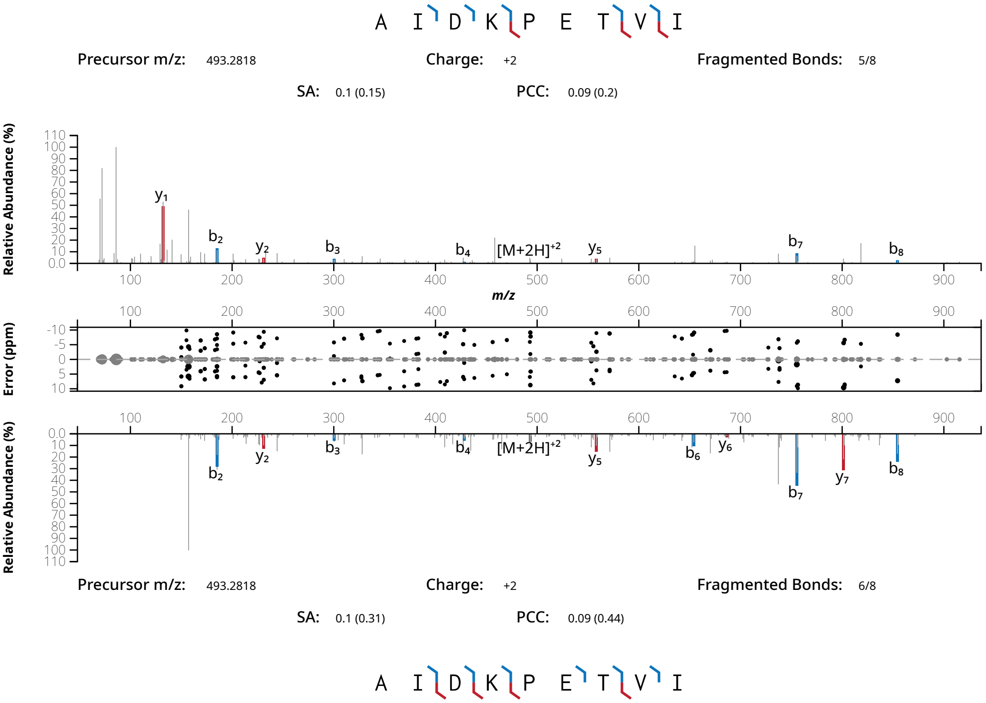
A I D K P E T V I
Precursor m/z: 493.2818
Charge: +2
Fragmented Bonds: 5/8
SA: 0.1 (0.15)
PCC: 0.09 (0.2)
Relative Abundance (%)
y1
b2
y2
b3
b4
[M+2H]+2
y5
b7
b8
m/z
Error (ppm)
Relative Abundance (%)
b2
y2
b3
b4
[M+2H]+2
y5
b6
y6
b7
y7
b8
Precursor m/z: 493.2818
Charge: +2
Fragmented Bonds: 6/8
SA: 0.1 (0.31)
PCC: 0.09 (0.44)
A I D K P E T V I

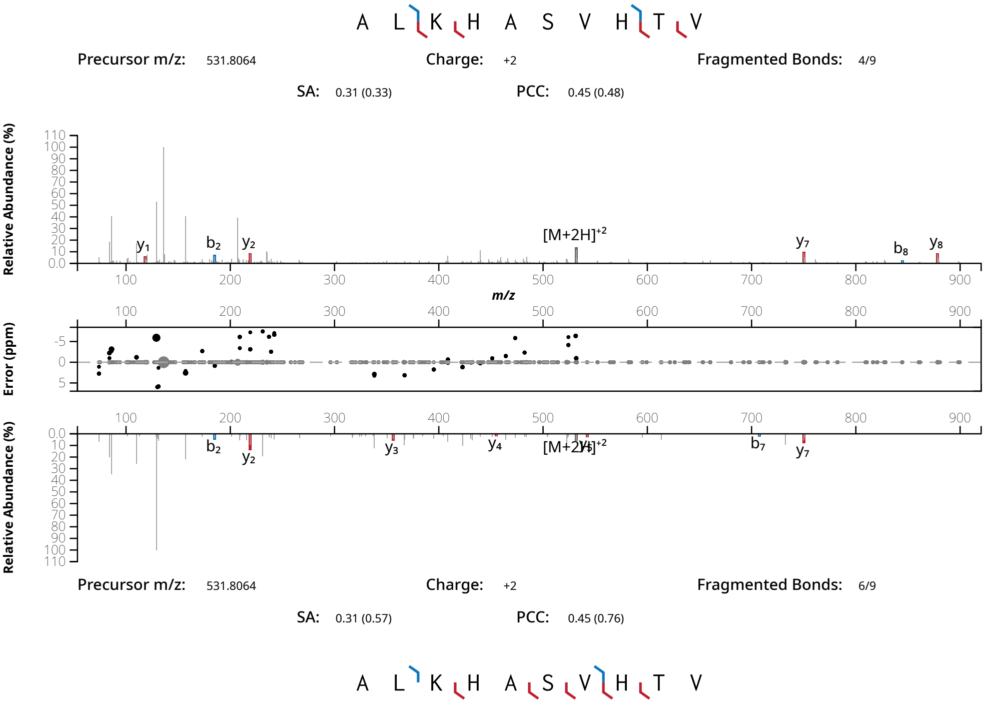
A L K H A S V H T V
Precursor m/z: 531.8064
Charge: +2
Fragmented Bonds: 4/9
SA: 0.31 (0.33)
PCC: 0.45 (0.48)
Relative Abundance (%)
Error (ppm)
m/z
[M+2H]+2
y1
b2
y2
y7
b8
y8
b2
y2
y3
y4
[M+2H]+2
b7
y7
Relative Abundance (%)
Precursor m/z: 531.8064
Charge: +2
Fragmented Bonds: 6/9
SA: 0.31 (0.57)
PCC: 0.45 (0.76)
A L K H A S V H T V

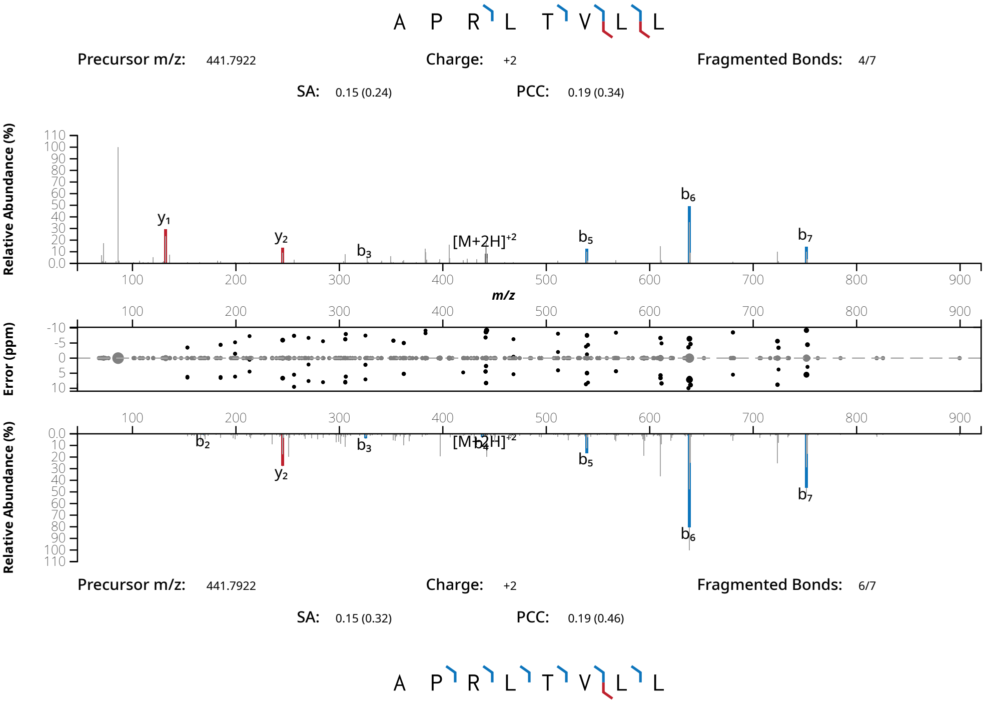

A P R L T V L L
Precursor m/z: 441.7922
Charge: +2
Fragmented Bonds: 4/7
SA: 0.15 (0.24)
PCC: 0.19 (0.34)
Relative Abundance (%)
y1
y2
b3
[M+2H]+2
b5
b6
b7
m/z
Error (ppm)
b2
b4
Precursor m/z: 441.7922
Charge: +2
Fragmented Bonds: 6/7
SA: 0.15 (0.32)
PCC: 0.19 (0.46)
A P R L T V L L

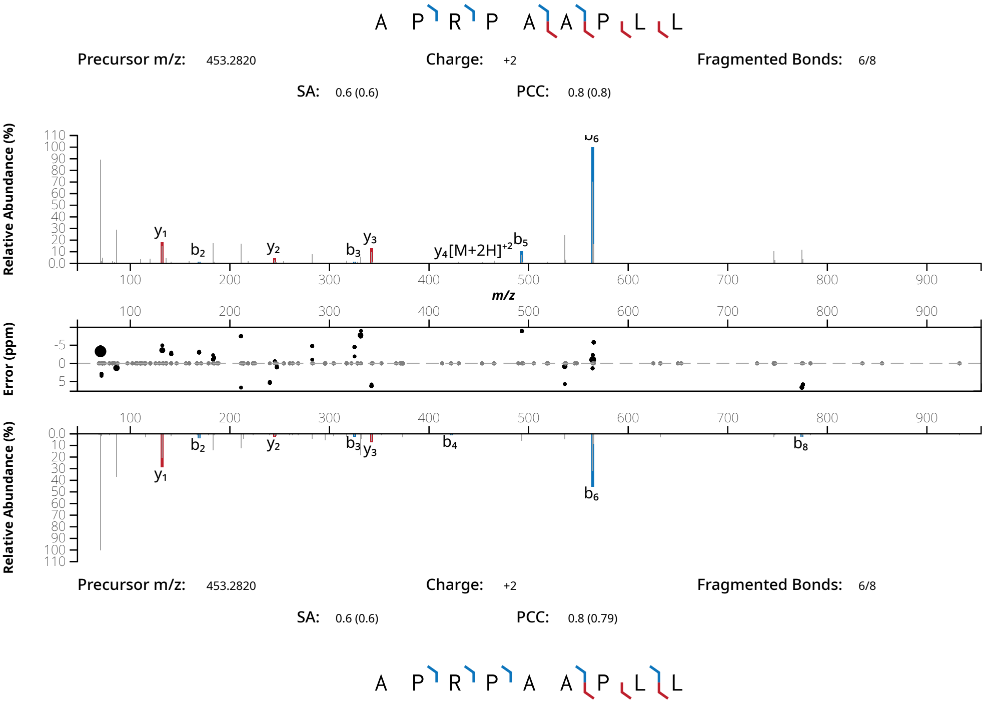
A P R P A A P L L
Precursor m/z: 453.2820
Charge: +2
Fragmented Bonds: 6/8
SA: 0.6 (0.6)
PCC: 0.8 (0.8)
Relative Abundance (%)
y1
b2
y2
b3
y3
y4[M+2H]+2
b5
b6
m/z
Error (ppm)
Relative Abundance (%)
y1
b2
y2
b3
y3
b4
b6
b8
Precursor m/z: 453.2820
Charge: +2
Fragmented Bonds: 6/8
SA: 0.6 (0.6)
PCC: 0.8 (0.79)
A P R P A A P L L

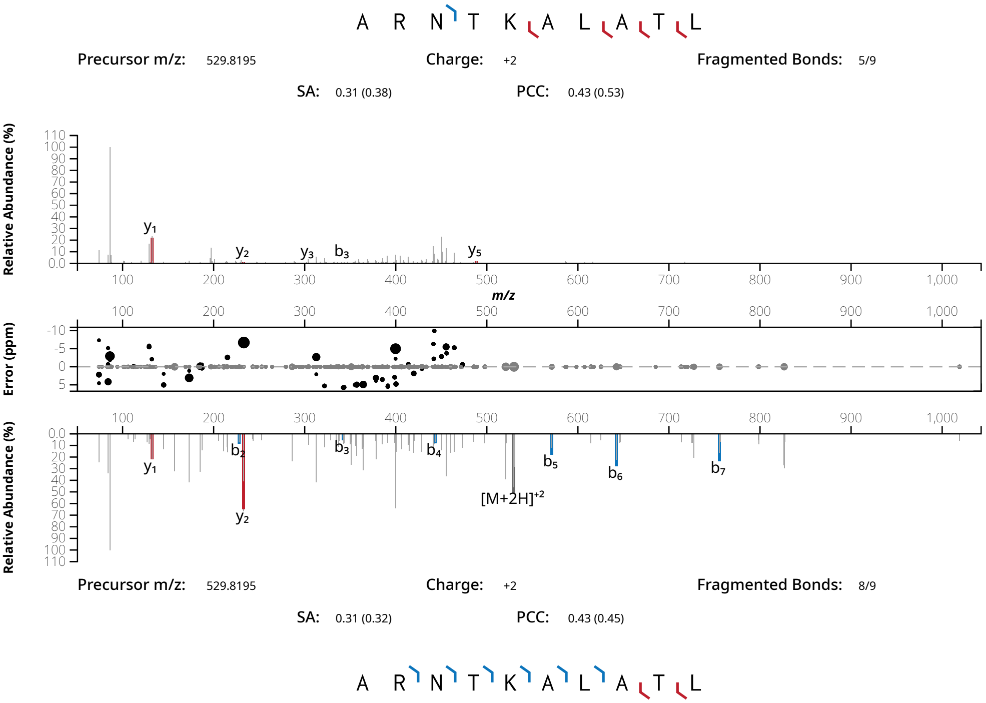
A R N T K A L A T L
Precursor m/z: 529.8195
Charge: +2
Fragmented Bonds: 5/9
SA: 0.31 (0.38)
PCC: 0.43 (0.53)
Relative Abundance (%)
y1
y2
y3
b3
y5
m/z
Error (ppm)
y1
b2
y2
b3
b4
[M+2H]+2
b5
b6
b7
Relative Abundance (%)
Precursor m/z: 529.8195
Charge: +2
Fragmented Bonds: 8/9
SA: 0.31 (0.32)
PCC: 0.43 (0.45)
A R N T K A L A T L

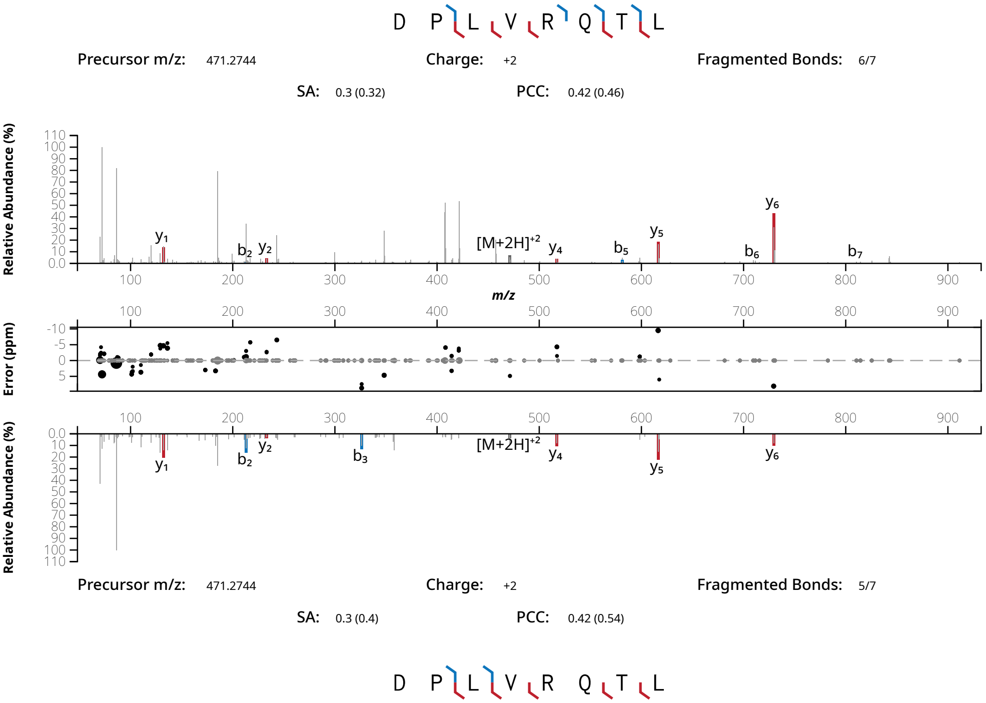
D P L V R Q T L
Precursor m/z: 471.2744
Charge: +2
Fragmented Bonds: 6/7
SA: 0.3 (0.32)
PCC: 0.42 (0.46)
Relative Abundance (%)
m/z
Error (ppm)
[M+2H]+2
Precursor m/z: 471.2744
Charge: +2
Fragmented Bonds: 5/7
SA: 0.3 (0.4)
PCC: 0.42 (0.54)
D P L V R Q T L

E A F K K H S L

Precursor m/z: 320.5151 **Charge:** +3 **Fragmented Bonds:** 6/7

SA: 0.68 (0.68) **PCC:** 0.87 (0.87)

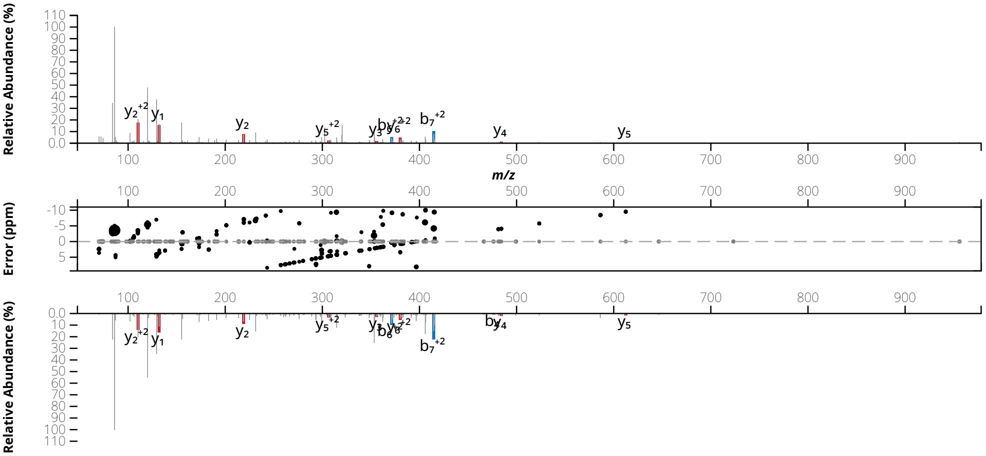

Precursor m/z: 320.5151 **Charge:** +3 **Fragmented Bonds:** 6/7

SA: 0.68 (0.7) **PCC:** 0.87 (0.88)

E A F K K H S L

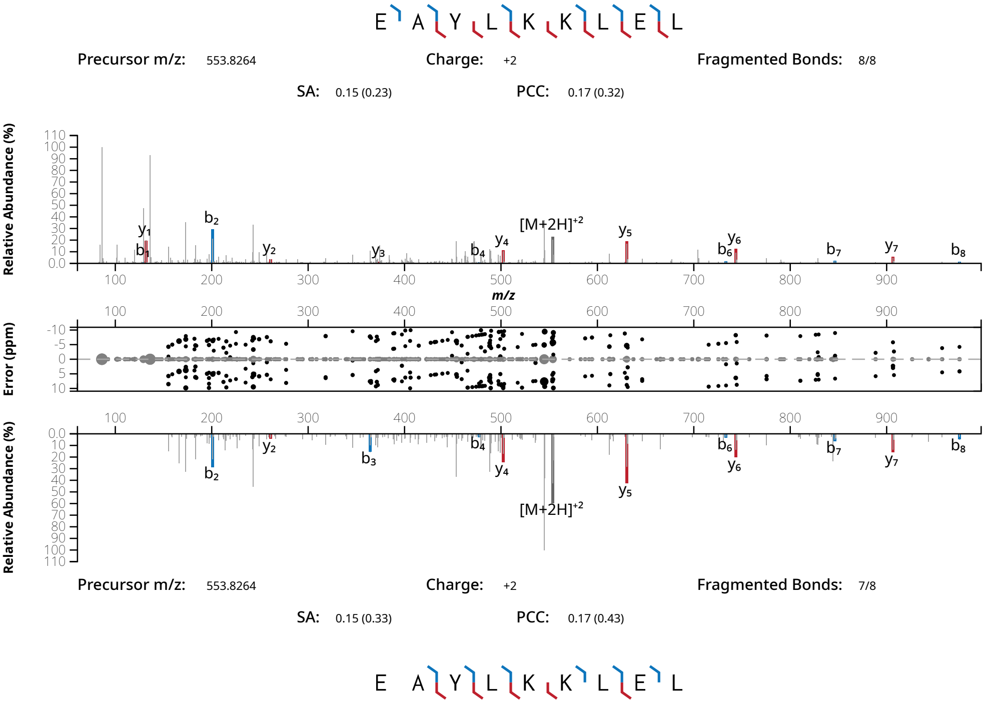
E A Y L K K L E L
Precursor m/z: 553.8264
Charge: +2
Fragmented Bonds: 8/8
SA: 0.15 (0.23)
PCC: 0.17 (0.32)
Relative Abundance (%)
Error (ppm)
m/z
[M+2H]+2
Precursor m/z: 553.8264
Charge: +2
Fragmented Bonds: 7/8
SA: 0.15 (0.33)
PCC: 0.17 (0.43)
E A Y L K K L E L

E G R E L R G S F

Precursor m/z: 525.7700 Charge: +2 Fragmented Bonds: 6/8

SA: 0.79 (0.8) PCC: 0.94 (0.95)

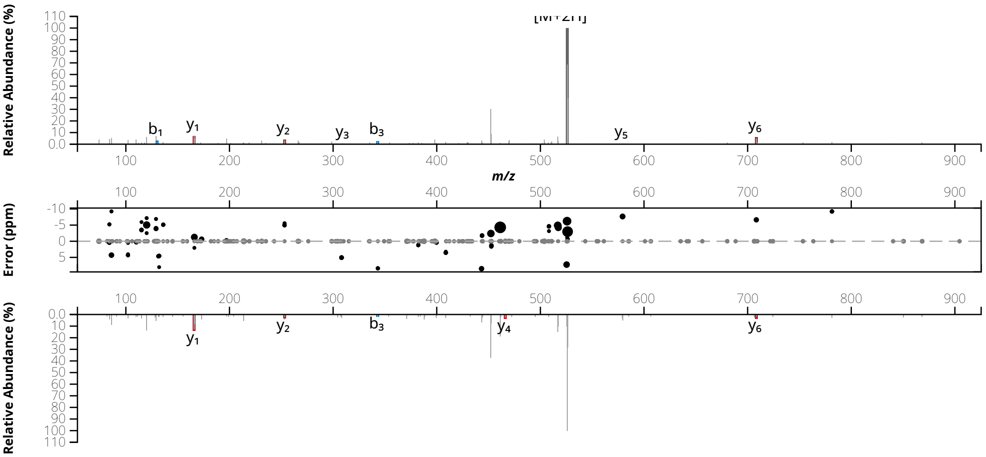

Precursor m/z: 525.7700 Charge: +2 Fragmented Bonds: 4/8

SA: 0.79 (0.8) PCC: 0.94 (0.95)

E G R E L R G S F

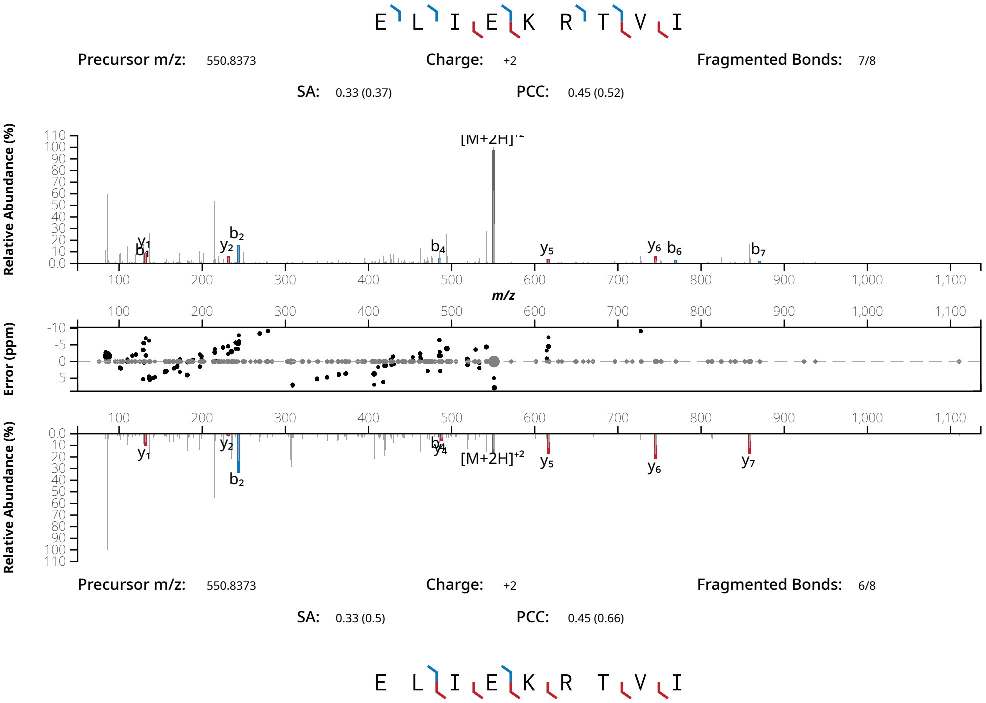
E L I E K R T V I
Precursor m/z: 550.8373
Charge: +2
Fragmented Bonds: 7/8
SA: 0.33 (0.37)
PCC: 0.45 (0.52)
Relative Abundance (%)
m/z
Error (ppm)
Relative Abundance (%)
Precursor m/z: 550.8373
Charge: +2
Fragmented Bonds: 6/8
SA: 0.33 (0.5)
PCC: 0.45 (0.66)
E L I E K R T V I

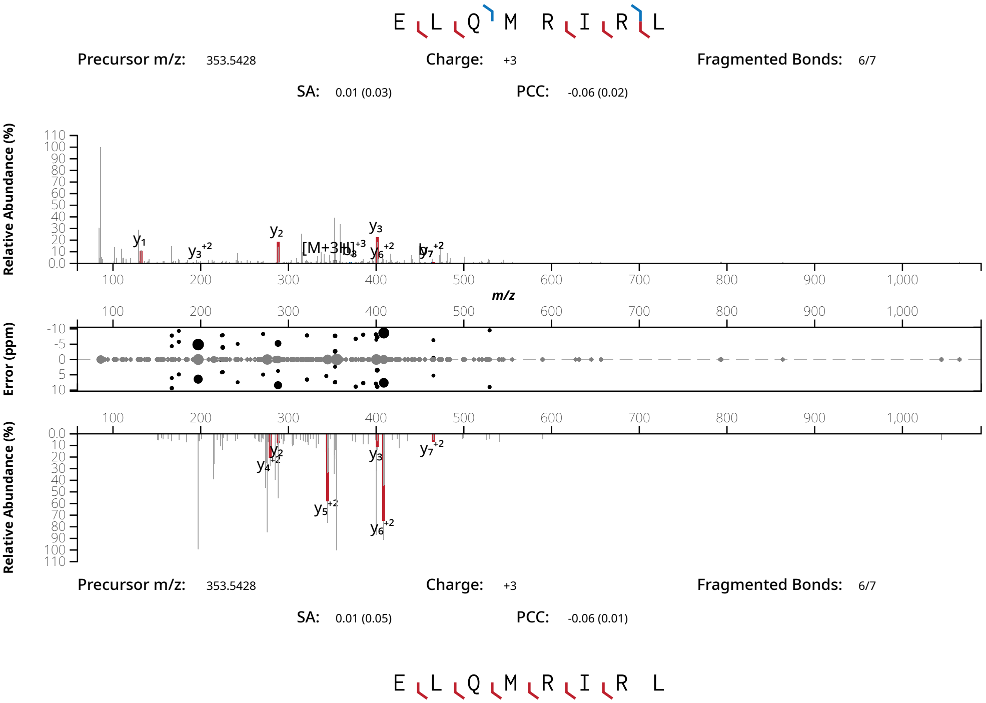
E L Q M R I R L
Precursor m/z: 353.5428
Charge: +3
Fragmented Bonds: 6/7
SA: 0.01 (0.03)
PCC: -0.06 (0.02)
Relative Abundance (%)
m/z
Error (ppm)
Relative Abundance (%)
Precursor m/z: 353.5428
Charge: +3
Fragmented Bonds: 6/7
SA: 0.01 (0.05)
PCC: -0.06 (0.01)
E L Q M R I R L

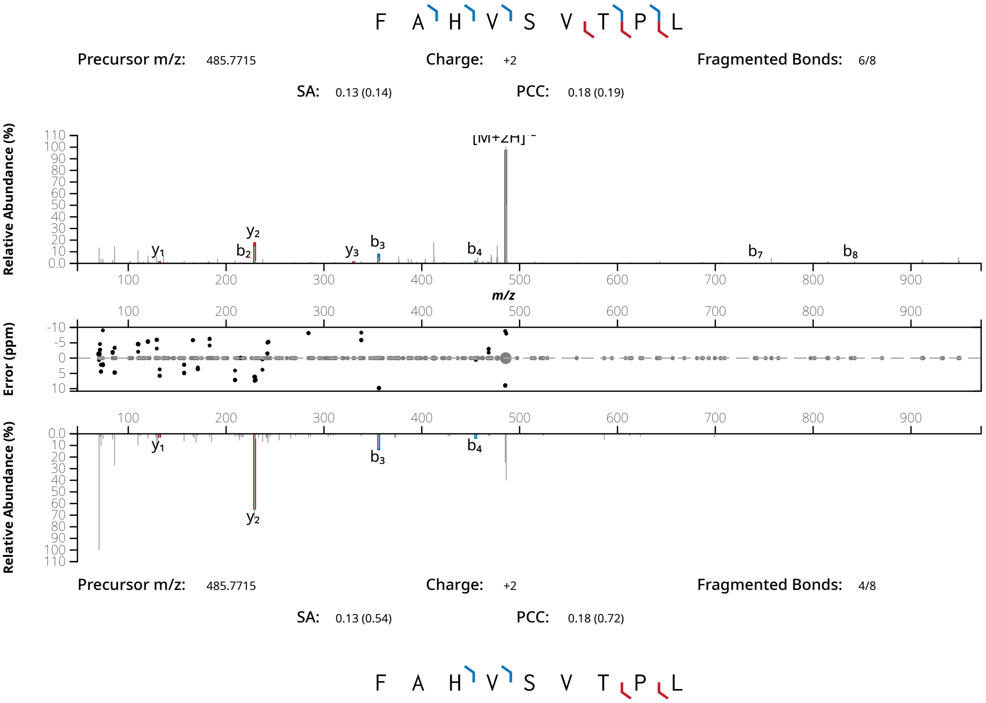
F A H V S V T P L
Precursor m/z: 485.7715
Charge: +2
Fragmented Bonds: 6/8
SA: 0.13 (0.14)
PCC: 0.18 (0.19)
Relative Abundance (%)
m/z
Error (ppm)
Relative Abundance (%)
Precursor m/z: 485.7715
Charge: +2
Fragmented Bonds: 4/8
SA: 0.13 (0.54)
PCC: 0.18 (0.72)
F A H V S V T P L

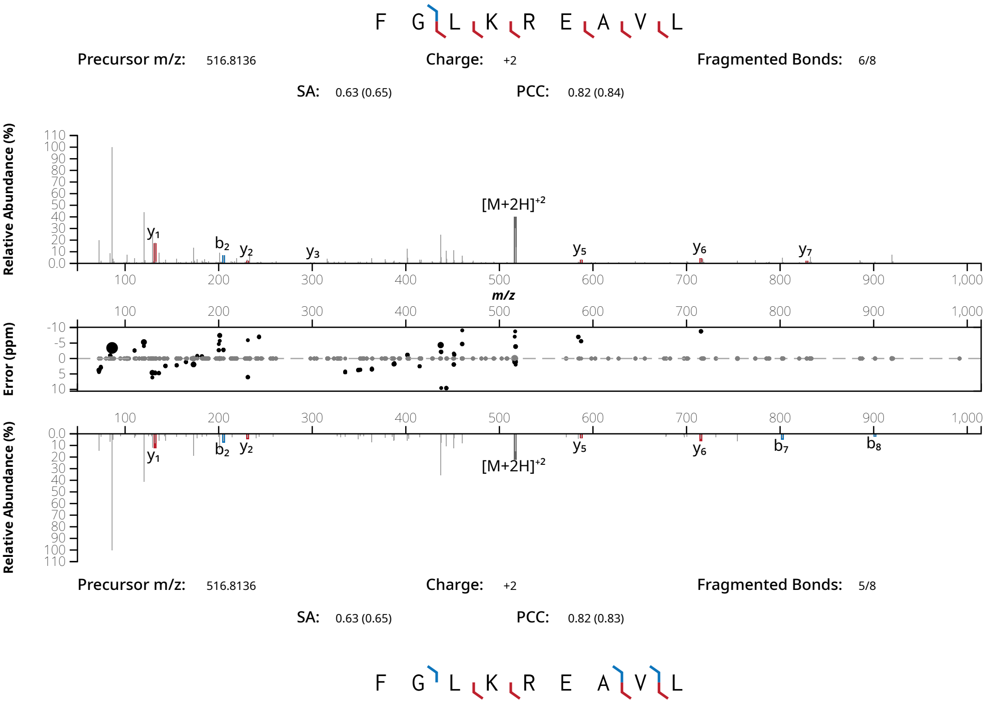
F G L K R E A V L
Precursor m/z: 516.8136
Charge: +2
Fragmented Bonds: 6/8
SA: 0.63 (0.65)
PCC: 0.82 (0.84)
Relative Abundance (%)
[M+2H]+2
y1
b2
y2
y3
y5
y6
y7
m/z
Error (ppm)
y1
b2
y2
y5
y6
b7
b8
[M+2H]+2
Relative Abundance (%)
Precursor m/z: 516.8136
Charge: +2
Fragmented Bonds: 5/8
SA: 0.63 (0.65)
PCC: 0.82 (0.83)
F G L K R E A V L

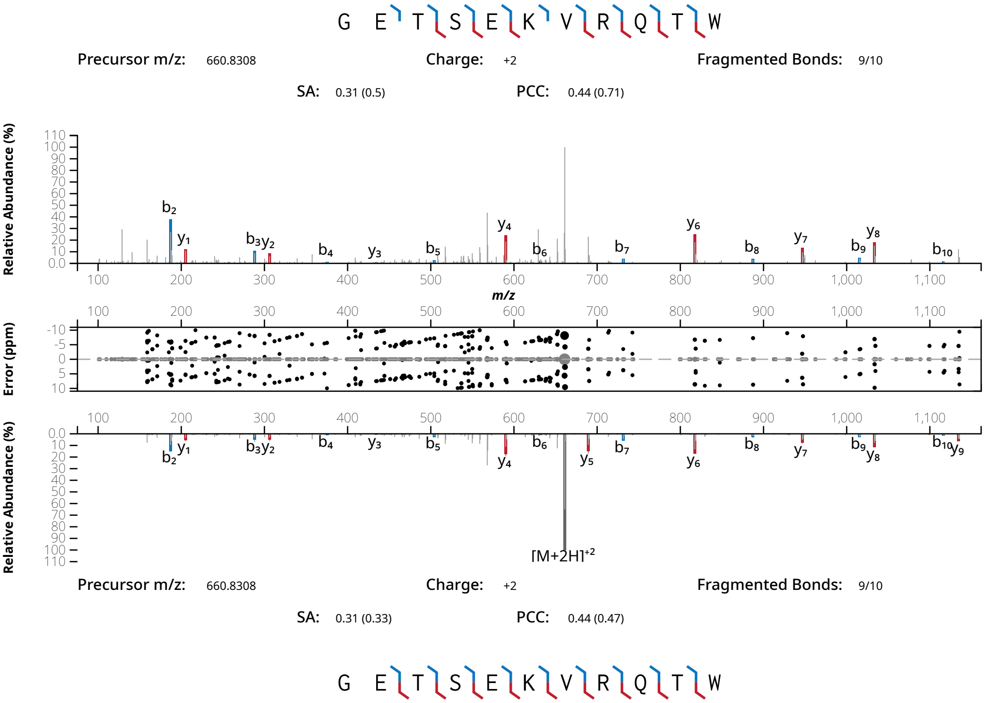
G E T S E K V R Q T W
Precursor m/z: 660.8308
Charge: +2
Fragmented Bonds: 9/10
SA: 0.31 (0.5)
PCC: 0.44 (0.71)
Relative Abundance (%)
m/z
Error (ppm)
[M+2H]+2
SA: 0.31 (0.33)
PCC: 0.44 (0.47)

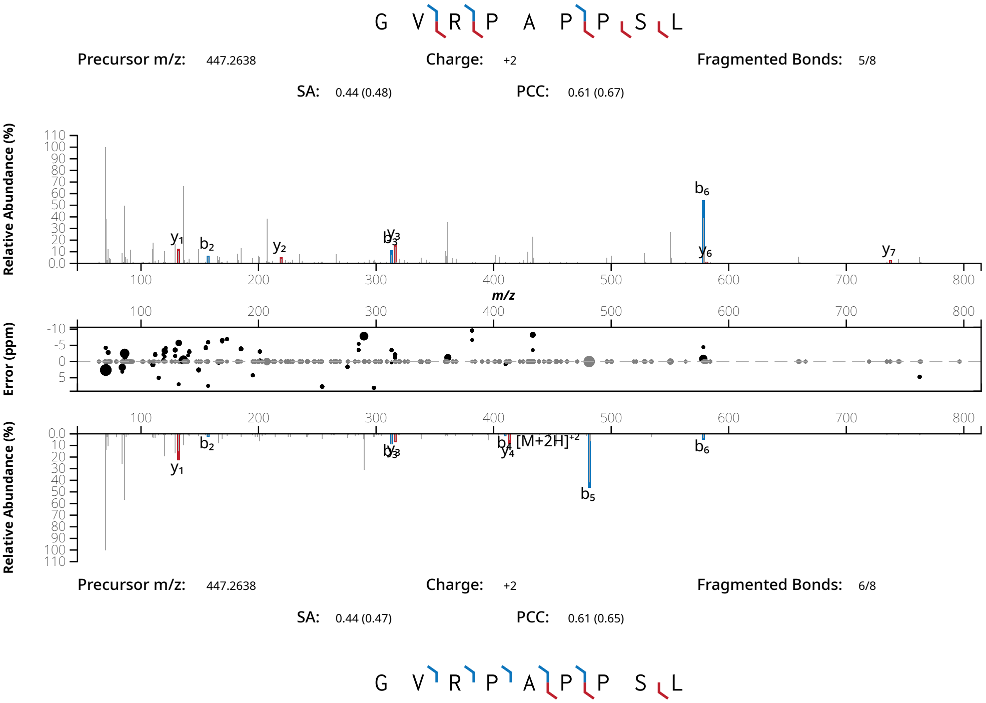
G V R P A P P S L
Precursor m/z: 447.2638
Charge: +2
Fragmented Bonds: 5/8
SA: 0.44 (0.48)
PCC: 0.61 (0.67)
Relative Abundance (%)
m/z
Error (ppm)
y1 b2 y2 b3 y3 b6 y6 y7
y1 b2 b3 y3 b4 y4 [M+2H]+2 b5 b6
Relative Abundance (%)
Precursor m/z: 447.2638
Charge: +2
Fragmented Bonds: 6/8
SA: 0.44 (0.47)
PCC: 0.61 (0.65)
G V R P A P P S L

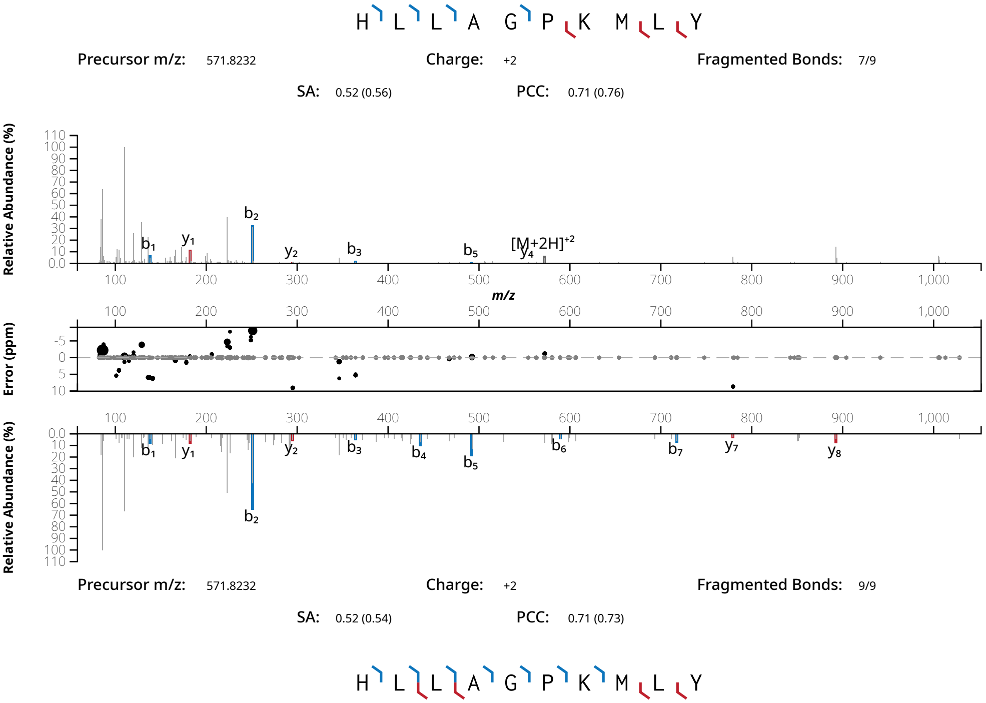
H L L A G P K M L Y
Precursor m/z: 571.8232
Charge: +2
Fragmented Bonds: 7/9
SA: 0.52 (0.56)
PCC: 0.71 (0.76)
Relative Abundance (%)
b1
y1
b2
y2
b3
b5
[M+2H]+2
y4
m/z
Error (ppm)
b4
b6
b7
y7
y8
Precursor m/z: 571.8232
Charge: +2
Fragmented Bonds: 9/9
SA: 0.52 (0.54)
PCC: 0.71 (0.73)
H L L A G P K M L Y

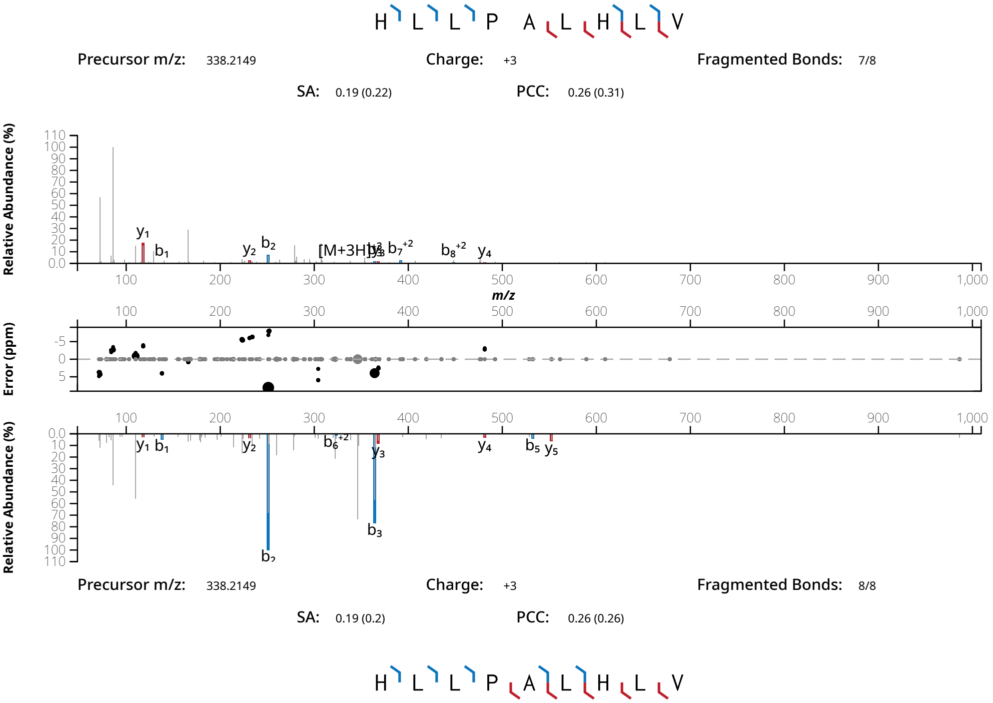

H L L P A L H L V
Precursor m/z: 338.2149
Charge: +3
Fragmented Bonds: 7/8
SA: 0.19 (0.22)
PCC: 0.26 (0.31)
Relative Abundance (%)
Error (ppm)
m/z
Precursor m/z: 338.2149
Charge: +3
Fragmented Bonds: 8/8
SA: 0.19 (0.2)
PCC: 0.26 (0.26)
H L L P A L H L V

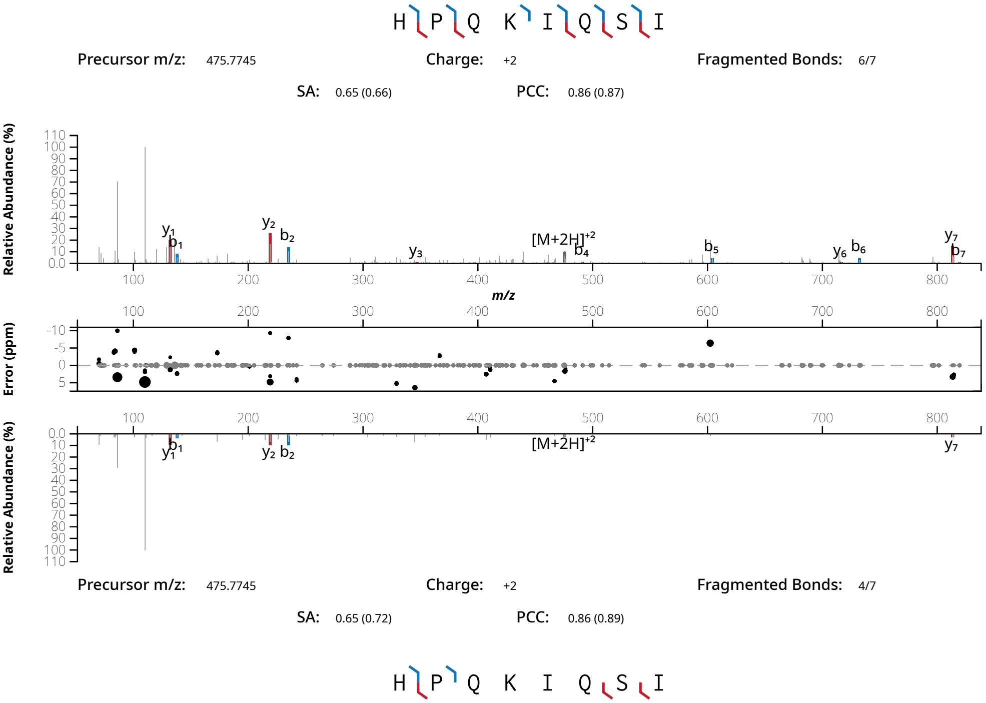
H P Q K I Q S I
Precursor m/z: 475.7745
Charge: +2
Fragmented Bonds: 6/7
SA: 0.65 (0.66)
PCC: 0.86 (0.87)
Relative Abundance (%)
m/z
Error (ppm)
Relative Abundance (%)
Precursor m/z: 475.7745
Charge: +2
Fragmented Bonds: 4/7
SA: 0.65 (0.72)
PCC: 0.86 (0.89)
H P Q K I Q S I

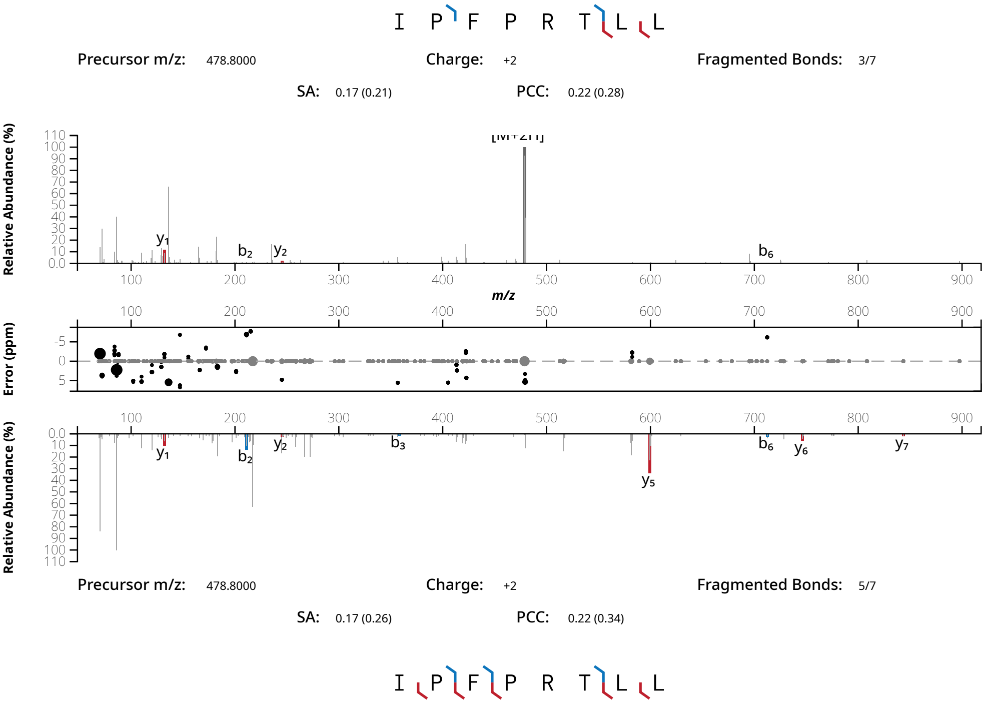
I P F P R T L L
Precursor m/z: 478.8000
Charge: +2
Fragmented Bonds: 3/7
SA: 0.17 (0.21)
PCC: 0.22 (0.28)
Relative Abundance (%)
y1
b2
y2
b6
m/z
Error (ppm)
Relative Abundance (%)
y1
b2
y2
b3
y5
b6
y6
y7
Precursor m/z: 478.8000
Charge: +2
Fragmented Bonds: 5/7
SA: 0.17 (0.26)
PCC: 0.22 (0.34)
I P F P R T L L

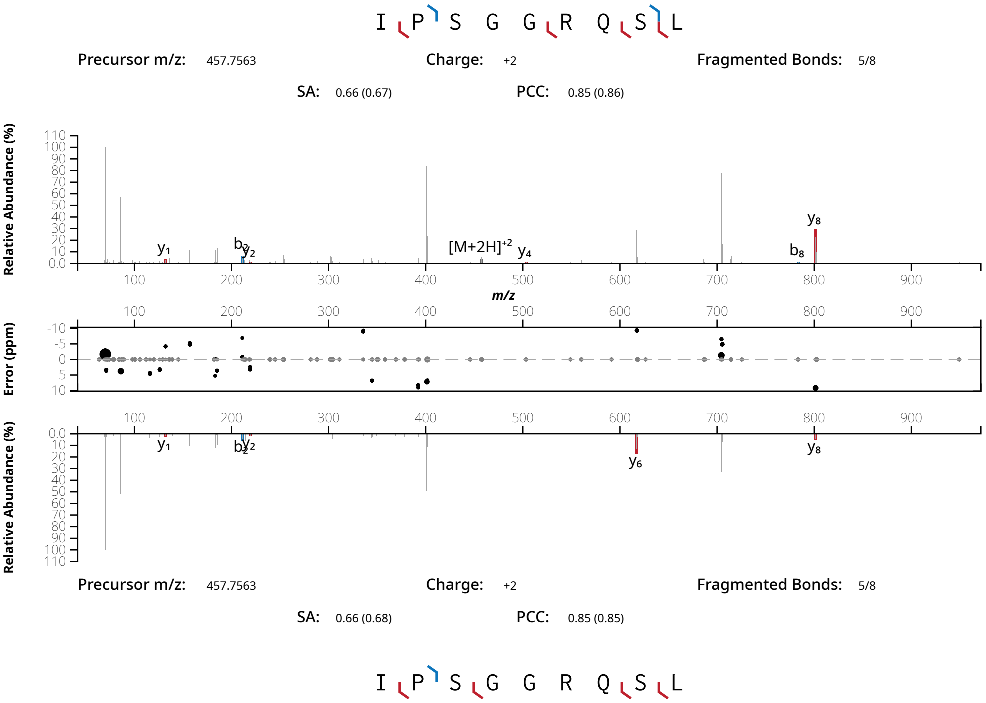
I P S G G R Q S L
Precursor m/z: 457.7563
Charge: +2
Fragmented Bonds: 5/8
SA: 0.66 (0.67)
PCC: 0.85 (0.86)
Relative Abundance (%)
y1
b2
y2
[M+2H]+2
y4
b8
y8
m/z
Error (ppm)
y6
Precursor m/z: 457.7563
Charge: +2
Fragmented Bonds: 5/8
SA: 0.66 (0.68)
PCC: 0.85 (0.85)

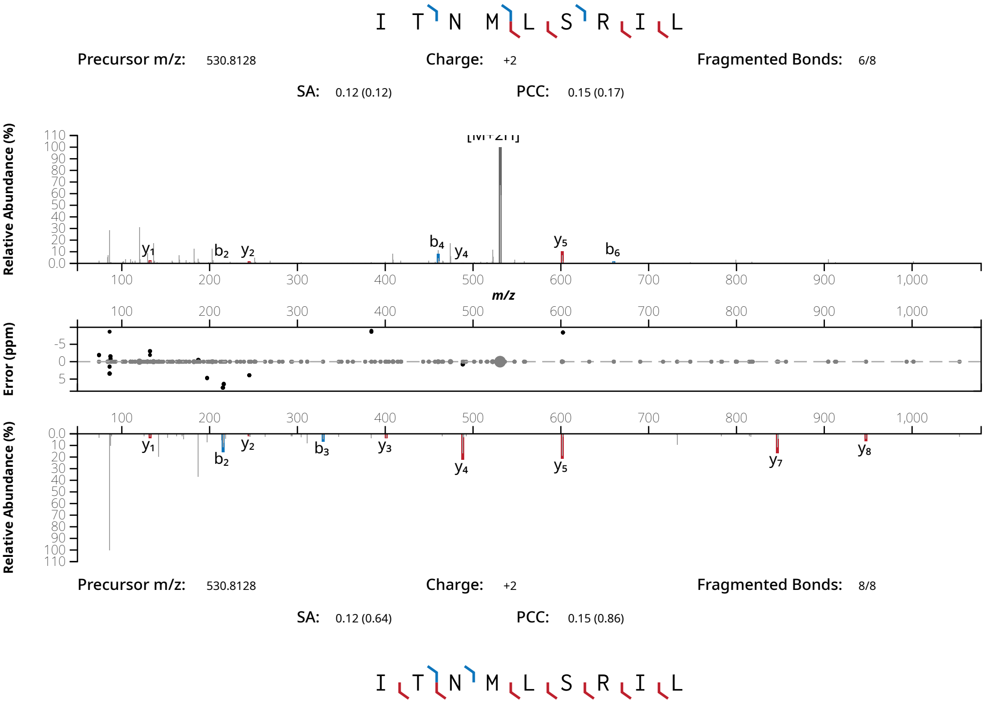
I T N M L S R I L
Precursor m/z: 530.8128
Charge: +2
Fragmented Bonds: 6/8
SA: 0.12 (0.12)
PCC: 0.15 (0.17)
Relative Abundance (%)
[M+2H]
y1
b2
y2
b4
y4
y5
b6
m/z
Error (ppm)
Relative Abundance (%)
y1
b2
y2
b3
y3
y4
y5
y7
y8
Precursor m/z: 530.8128
Charge: +2
Fragmented Bonds: 8/8
SA: 0.12 (0.64)
PCC: 0.15 (0.86)
I T N M L S R I L

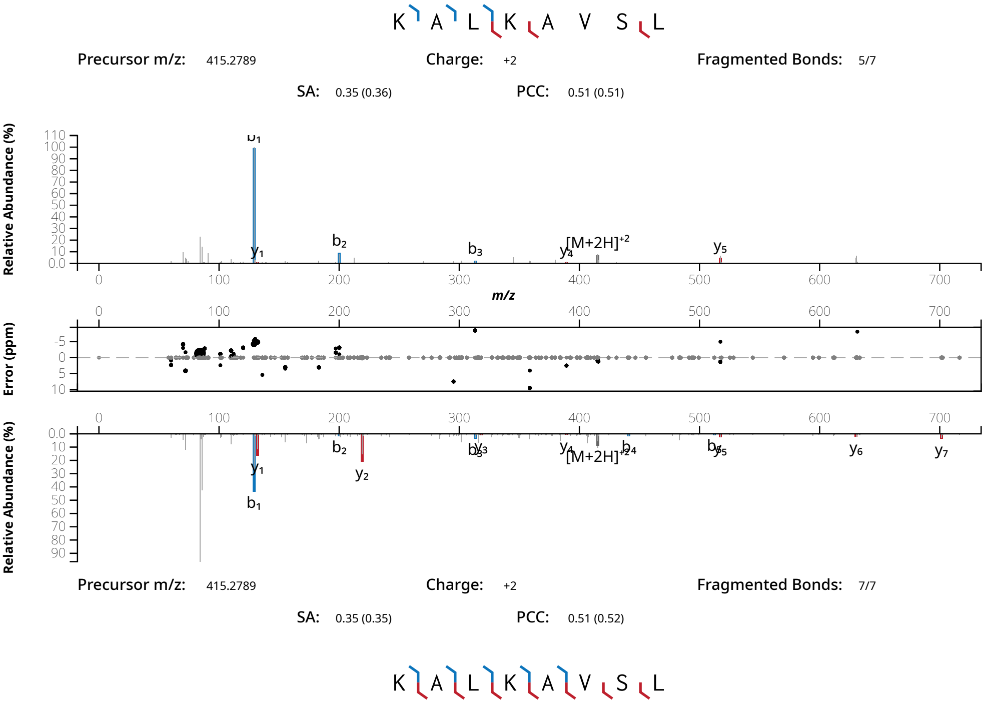
K A L K A V S L
Precursor m/z: 415.2789
Charge: +2
Fragmented Bonds: 5/7
SA: 0.35 (0.36)
PCC: 0.51 (0.51)
Relative Abundance (%)
b1
y1
b2
b3
y4
[M+2H]+2
y5
m/z
Error (ppm)
y1
b1
b2
y2
b3
y3
y4
[M+2H]+2
b4
b5
y5
y6
y7
Relative Abundance (%)
Precursor m/z: 415.2789
Charge: +2
Fragmented Bonds: 7/7
SA: 0.35 (0.35)
PCC: 0.51 (0.52)
K A L K A V S L

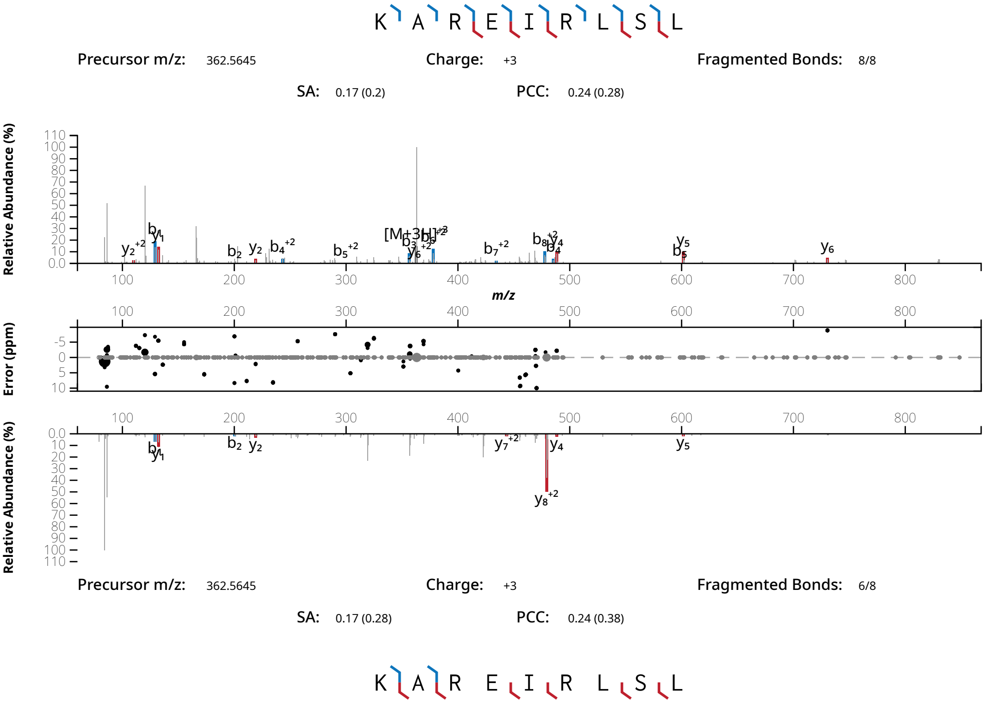
K A R E I R L S L
Precursor m/z: 362.5645
Charge: +3
Fragmented Bonds: 8/8
SA: 0.17 (0.2)
PCC: 0.24 (0.28)
Relative Abundance (%)
m/z
Error (ppm)
Relative Abundance (%)
Precursor m/z: 362.5645
Charge: +3
Fragmented Bonds: 6/8
SA: 0.17 (0.28)
PCC: 0.24 (0.38)
K A R E I R L S L

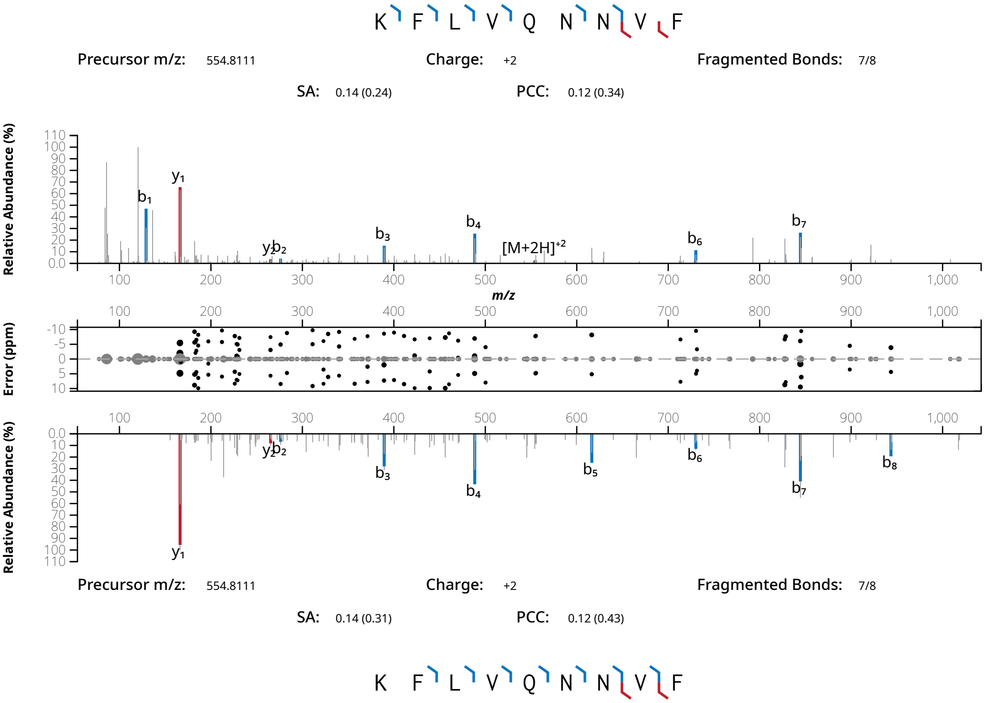
K F L V Q N N V F
Precursor m/z: 554.8111
Charge: +2
Fragmented Bonds: 7/8
SA: 0.14 (0.24)
PCC: 0.12 (0.34)
Relative Abundance (%)
b1
y1
y2
b2
b3
b4
[M+2H]+2
b6
b7
m/z
Error (ppm)
b5
b8
Precursor m/z: 554.8111
Charge: +2
Fragmented Bonds: 7/8
SA: 0.14 (0.31)
PCC: 0.12 (0.43)
K F L V Q N N V F

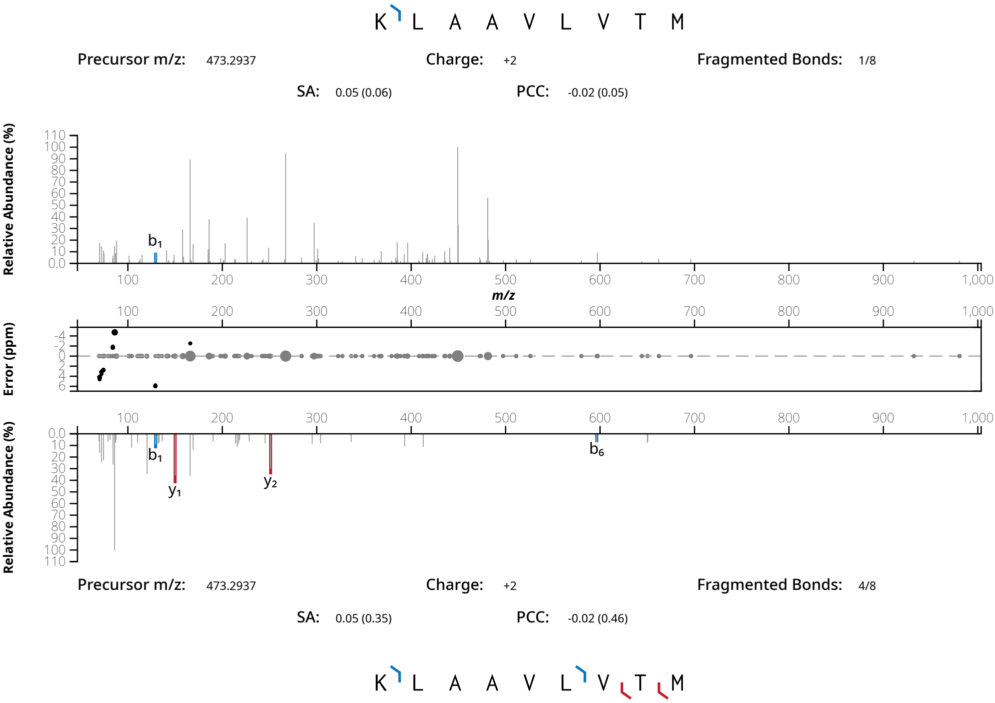
K L A A V L V T M
Precursor m/z: 473.2937
Charge: +2
Fragmented Bonds: 1/8
SA: 0.05 (0.06)
PCC: -0.02 (0.05)
Relative Abundance (%)
m/z
Error (ppm)
b1
y1
y2
b6
Precursor m/z: 473.2937
Charge: +2
Fragmented Bonds: 4/8
SA: 0.05 (0.35)
PCC: -0.02 (0.46)
K L A A V L V T M

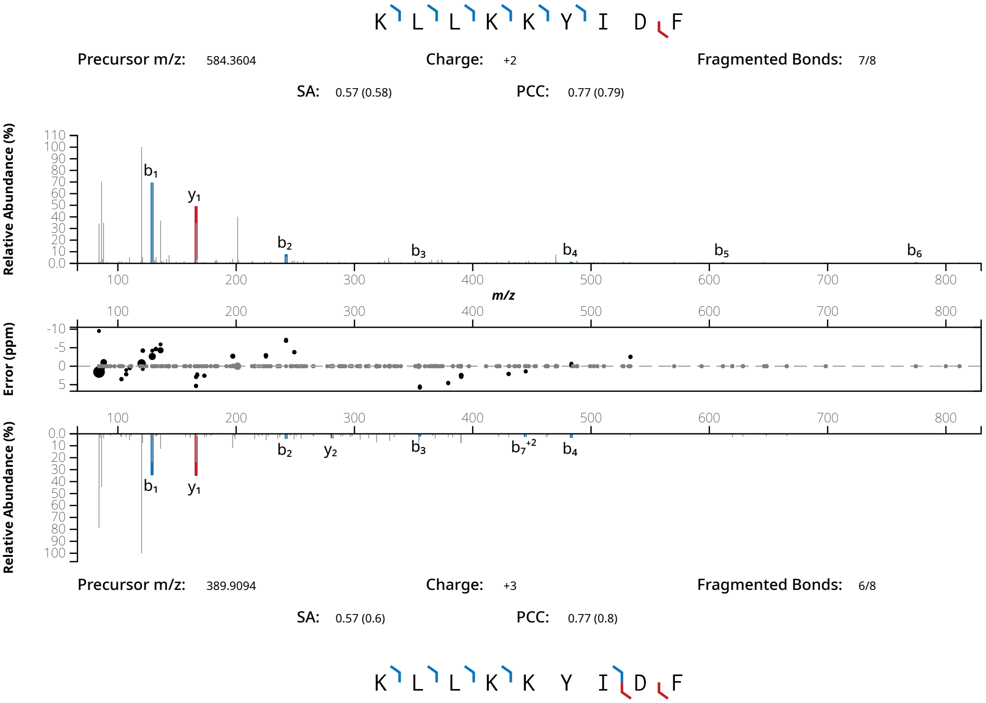
K L L K K Y I D F
Precursor m/z: 584.3604
Charge: +2
Fragmented Bonds: 7/8
SA: 0.57 (0.58)
PCC: 0.77 (0.79)
Relative Abundance (%)
Error (ppm)
m/z
b1
y1
b2
b3
b4
b5
b6
y2
b7+2
Precursor m/z: 389.9094
Charge: +3
Fragmented Bonds: 6/8
SA: 0.57 (0.6)
PCC: 0.77 (0.8)
K L L K K Y I D F

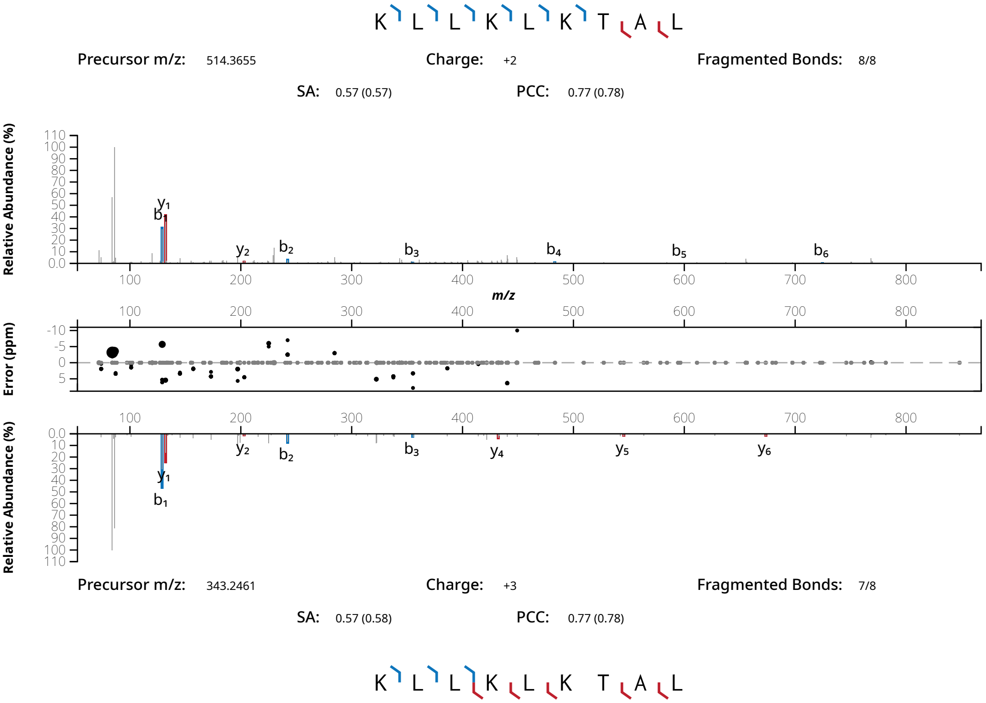
K L L K L K T A L
Precursor m/z: 514.3655
Charge: +2
Fragmented Bonds: 8/8
SA: 0.57 (0.57)
PCC: 0.77 (0.78)
Relative Abundance (%)
m/z
Error (ppm)
Precursor m/z: 343.2461
Charge: +3
Fragmented Bonds: 7/8
SA: 0.57 (0.58)
PCC: 0.77 (0.78)
K L L K L K T A L

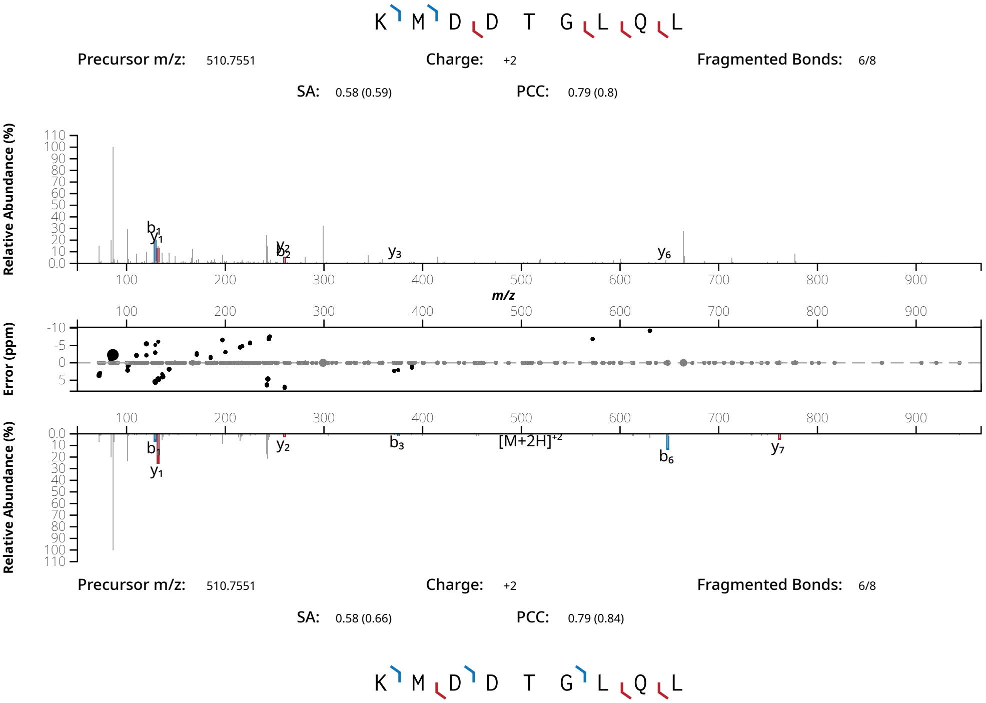

K M D D T G L Q L
Precursor m/z: 510.7551
Charge: +2
Fragmented Bonds: 6/8
SA: 0.58 (0.59)
PCC: 0.79 (0.8)
Relative Abundance (%)
b1
y1
y2
b2
y3
y6
m/z
Error (ppm)
Relative Abundance (%)
b1
y1
y2
b3
[M+2H]+2
b6
y7
Precursor m/z: 510.7551
Charge: +2
Fragmented Bonds: 6/8
SA: 0.58 (0.66)
PCC: 0.79 (0.84)
K M D D T G L Q L

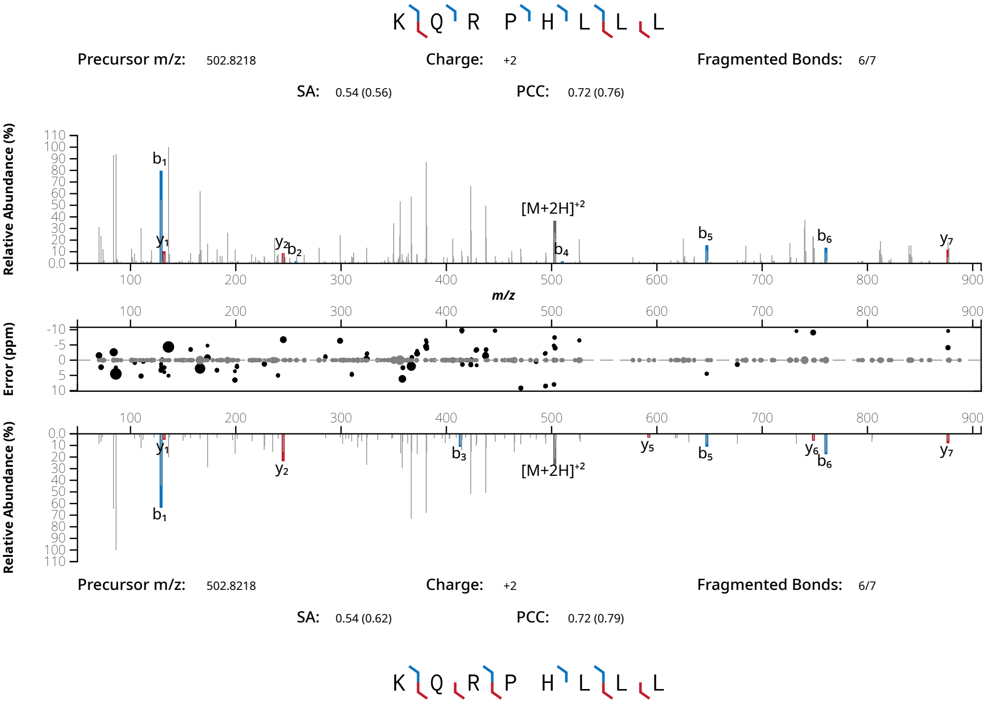
K Q R P H L L L
Precursor m/z: 502.8218
Charge: +2
Fragmented Bonds: 6/7
SA: 0.54 (0.56)
PCC: 0.72 (0.76)
Relative Abundance (%)
m/z
Error (ppm)
Relative Abundance (%)
Precursor m/z: 502.8218
Charge: +2
Fragmented Bonds: 6/7
SA: 0.54 (0.62)
PCC: 0.72 (0.79)
K Q R P H L L L

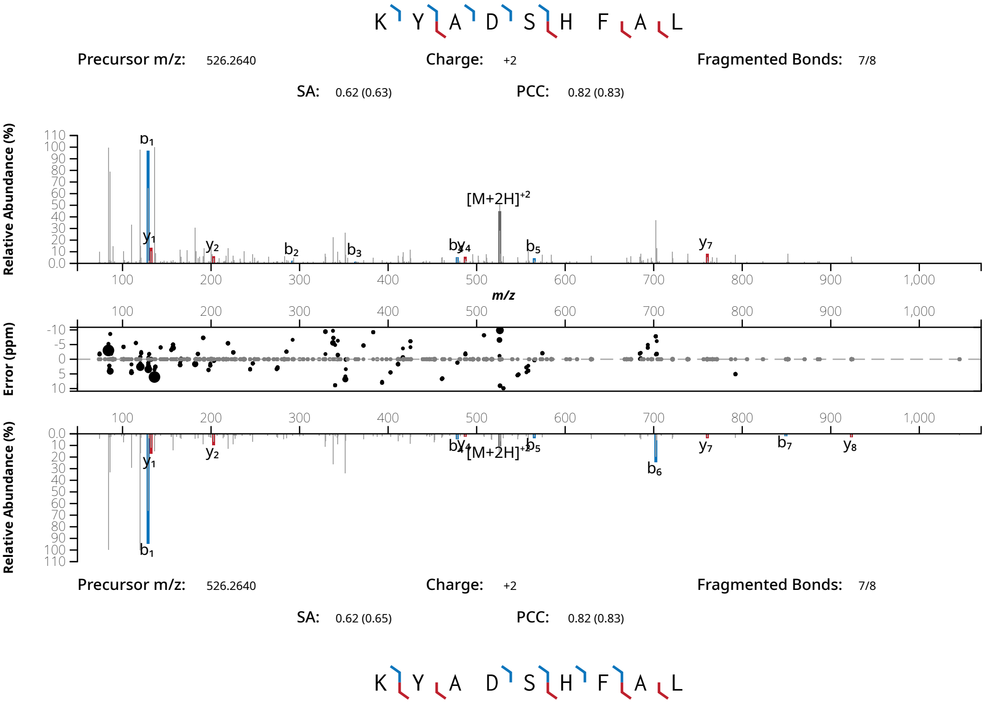
K Y A D S H F A L
Precursor m/z: 526.2640
Charge: +2
Fragmented Bonds: 7/8
SA: 0.62 (0.63)
PCC: 0.82 (0.83)
Relative Abundance (%)
b1
y1
y2
b2
b3
[M+2H]+2
by4
b5
y7
m/z
Error (ppm)
Relative Abundance (%)
y1
b1
y2
by4
[M+2H]+2
b5
b6
y7
b7
y8
Precursor m/z: 526.2640
Charge: +2
Fragmented Bonds: 7/8
SA: 0.62 (0.65)
PCC: 0.82 (0.83)
K Y A D S H F A L

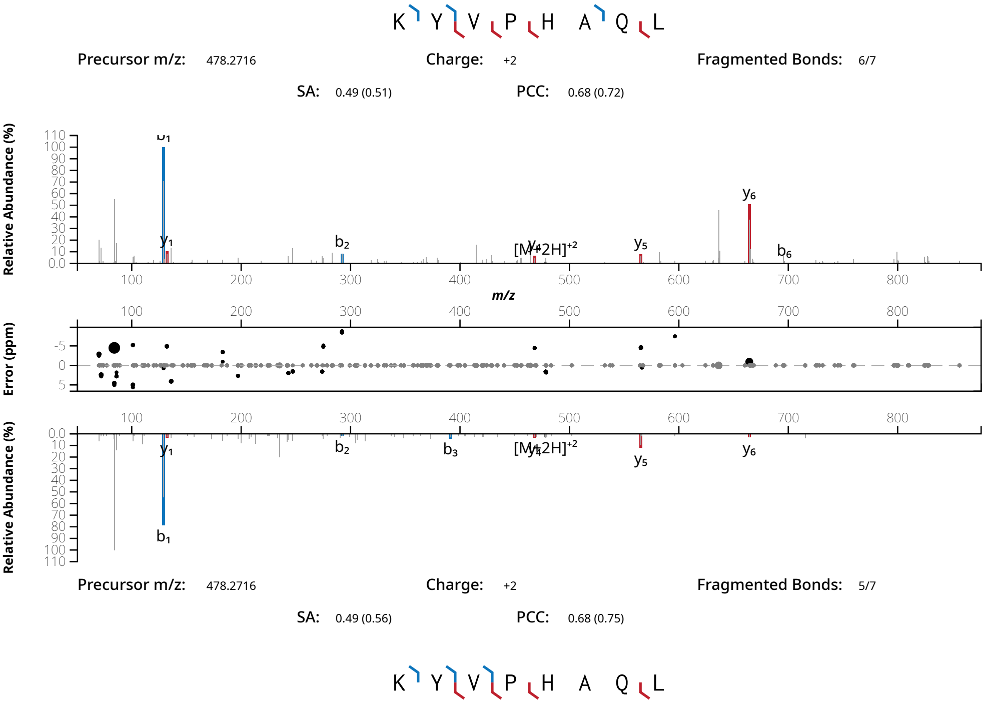
K Y V P H A Q L
Precursor m/z: 478.2716
Charge: +2
Fragmented Bonds: 6/7
SA: 0.49 (0.51)
PCC: 0.68 (0.72)
Relative Abundance (%)
b1
y1
b2
[M+2H]+2
y5
y6
b6
m/z
Error (ppm)
Relative Abundance (%)
y1
b2
b3
[M+2H]+2
y5
y6
b1
Precursor m/z: 478.2716
Charge: +2
Fragmented Bonds: 5/7
SA: 0.49 (0.56)
PCC: 0.68 (0.75)
K Y V P H A Q L

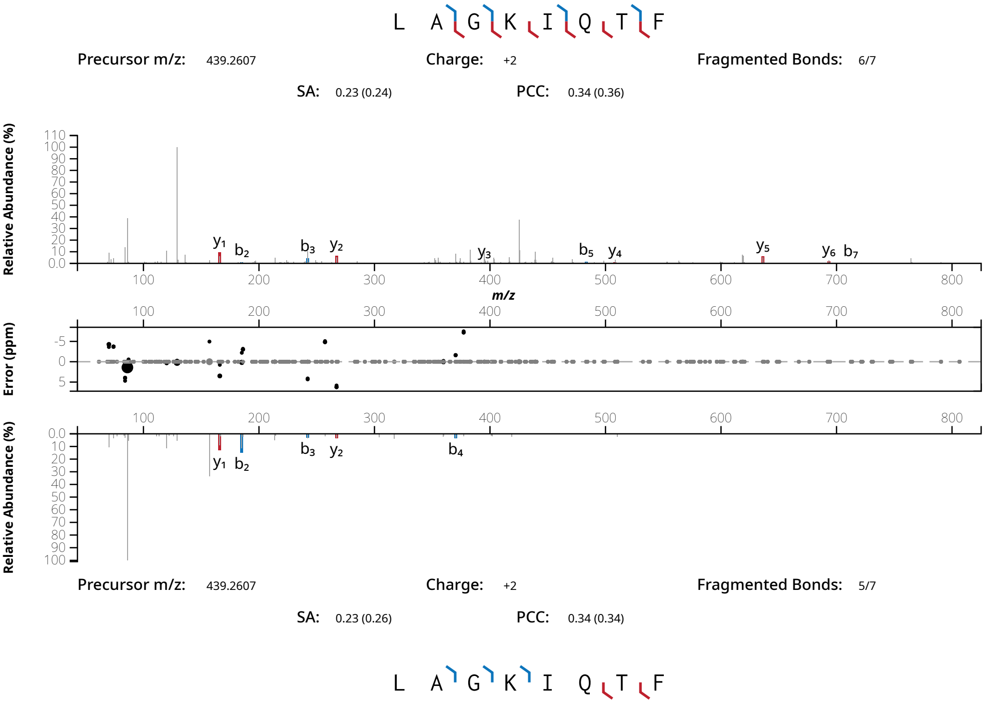

L A G K I Q T F
Precursor m/z: 439.2607
Charge: +2
Fragmented Bonds: 6/7
SA: 0.23 (0.24)
PCC: 0.34 (0.36)
Relative Abundance (%)
Error (ppm)
m/z
y1 b2 b3 y2 y3 b5 y4 y5 y6 b7
y1 b2 b3 y2 b4
Precursor m/z: 439.2607
Charge: +2
Fragmented Bonds: 5/7
SA: 0.23 (0.26)
PCC: 0.34 (0.34)
L A G K I Q T F

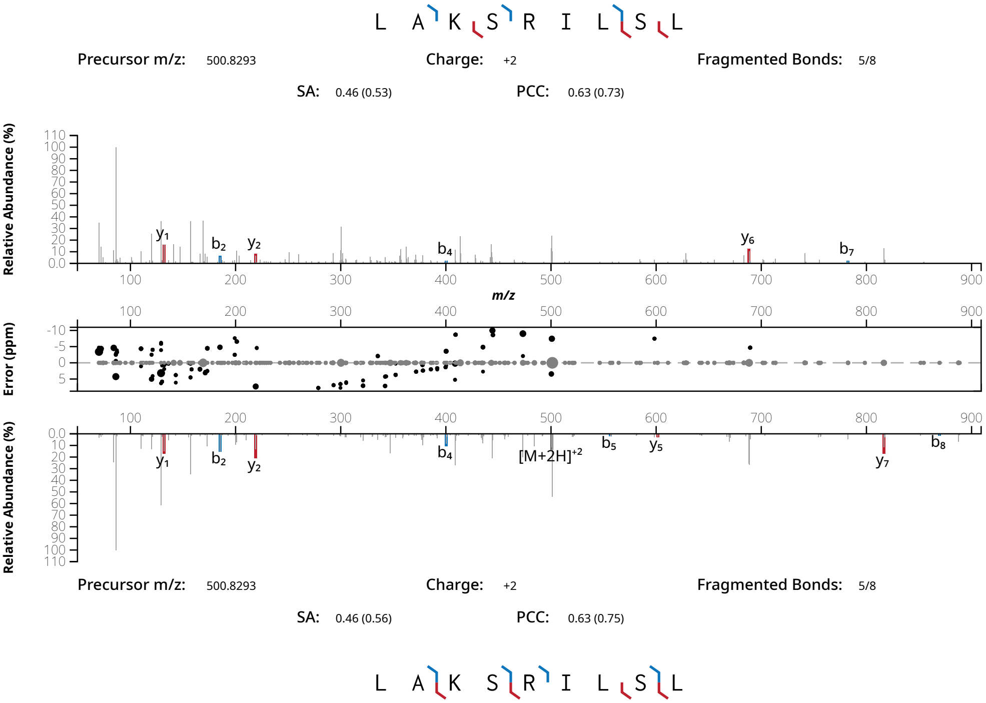
L A K S R I L S L
Precursor m/z: 500.8293
Charge: +2
Fragmented Bonds: 5/8
SA: 0.46 (0.53)
PCC: 0.63 (0.73)
Relative Abundance (%)
y1
b2
y2
b4
y6
b7
m/z
Error (ppm)
Relative Abundance (%)
y1
b2
y2
b4
[M+2H]+2
b5
y5
y7
b8
Precursor m/z: 500.8293
Charge: +2
Fragmented Bonds: 5/8
SA: 0.46 (0.56)
PCC: 0.63 (0.75)
L A K S R I L S L

L D M K R N R A L

Precursor m/z: 558.8189 Charge: +2 Fragmented Bonds: 7/8

SA: 0.69 (0.7) PCC: 0.88 (0.89)

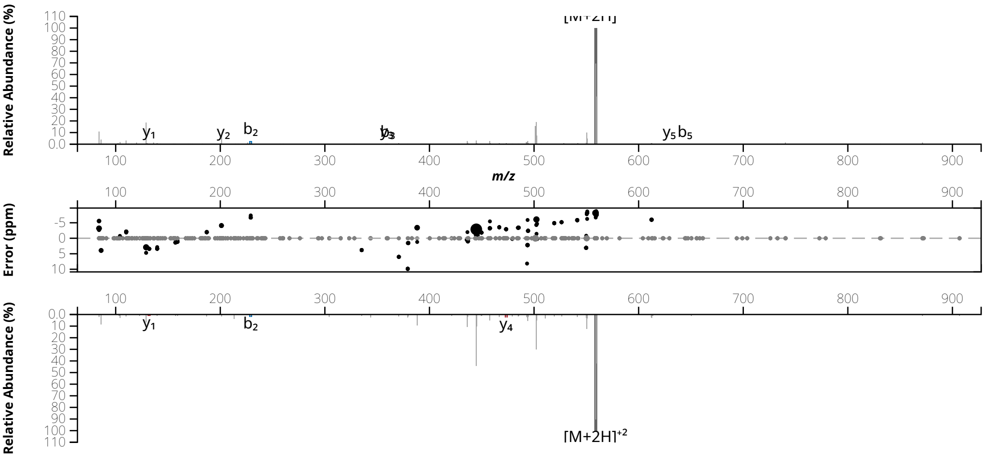

Precursor m/z: 558.8189 Charge: +2 Fragmented Bonds: 3/8

SA: 0.69 (0.71) PCC: 0.88 (0.89)

L D M K R N R A L

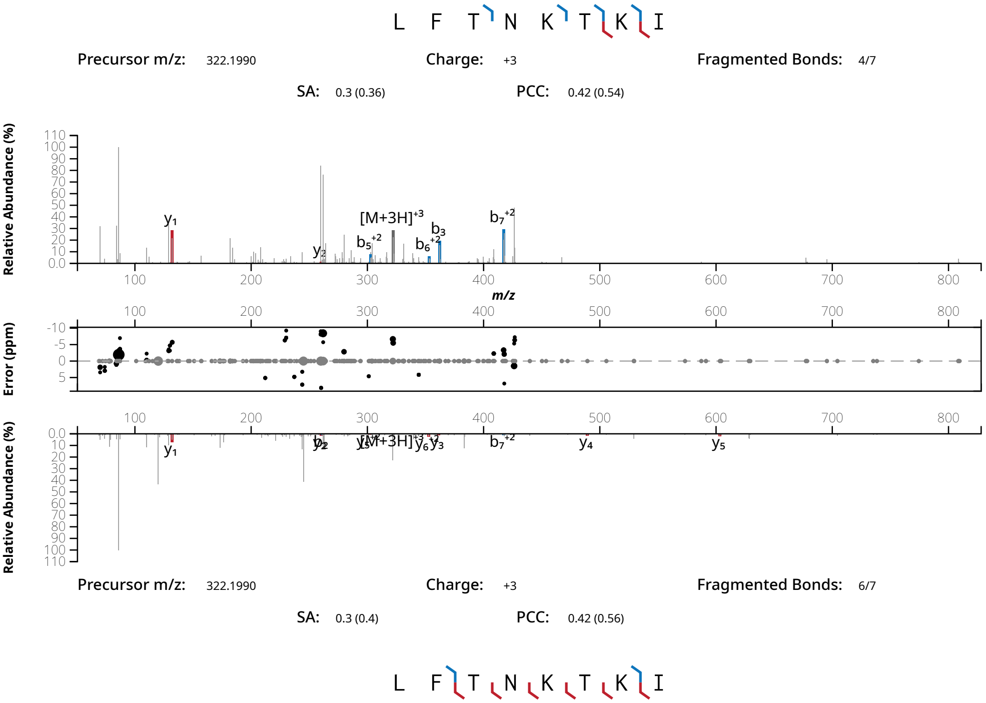
L F T N K T K I
Precursor m/z: 322.1990
Charge: +3
Fragmented Bonds: 4/7
SA: 0.3 (0.36)
PCC: 0.42 (0.54)
Relative Abundance (%)
Error (ppm)
m/z
Precursor m/z: 322.1990
Charge: +3
Fragmented Bonds: 6/7
SA: 0.3 (0.4)
PCC: 0.42 (0.56)
L F T N K T K I

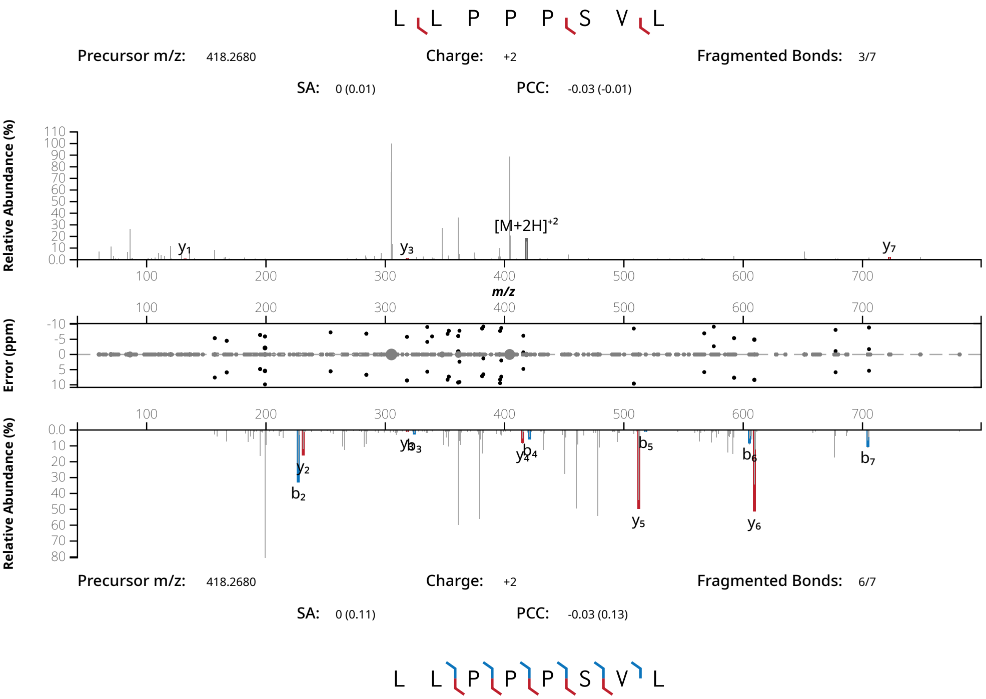
L L P P P S V L
Precursor m/z: 418.2680
Charge: +2
Fragmented Bonds: 3/7
SA: 0 (0.01)
PCC: -0.03 (-0.01)
Relative Abundance (%)
[M+2H]+2
y1
y3
y7
m/z
Error (ppm)
b2
y2
b3
y4
b4
b5
y5
b6
y6
b7
Precursor m/z: 418.2680
Charge: +2
Fragmented Bonds: 6/7
SA: 0 (0.11)
PCC: -0.03 (0.13)
L L P P P S V L

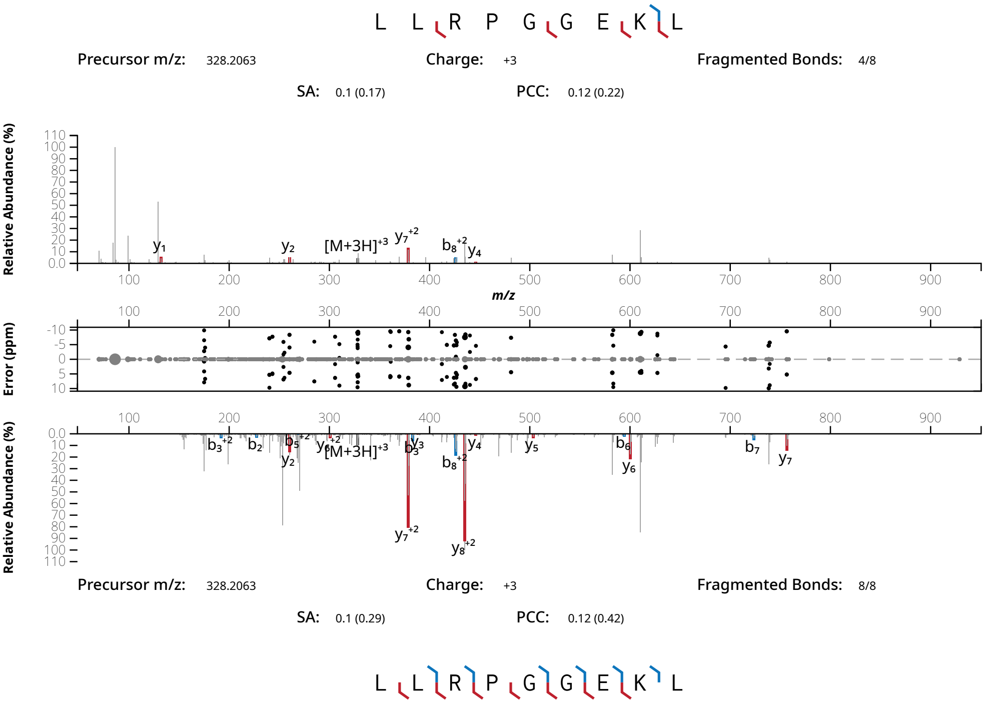
L L R P G G E K L
Precursor m/z: 328.2063
Charge: +3
Fragmented Bonds: 4/8
SA: 0.1 (0.17)
PCC: 0.12 (0.22)
Relative Abundance (%)
m/z
Error (ppm)
Relative Abundance (%)
Precursor m/z: 328.2063
Charge: +3
Fragmented Bonds: 8/8
SA: 0.1 (0.29)
PCC: 0.12 (0.42)
L L R P G G E K L

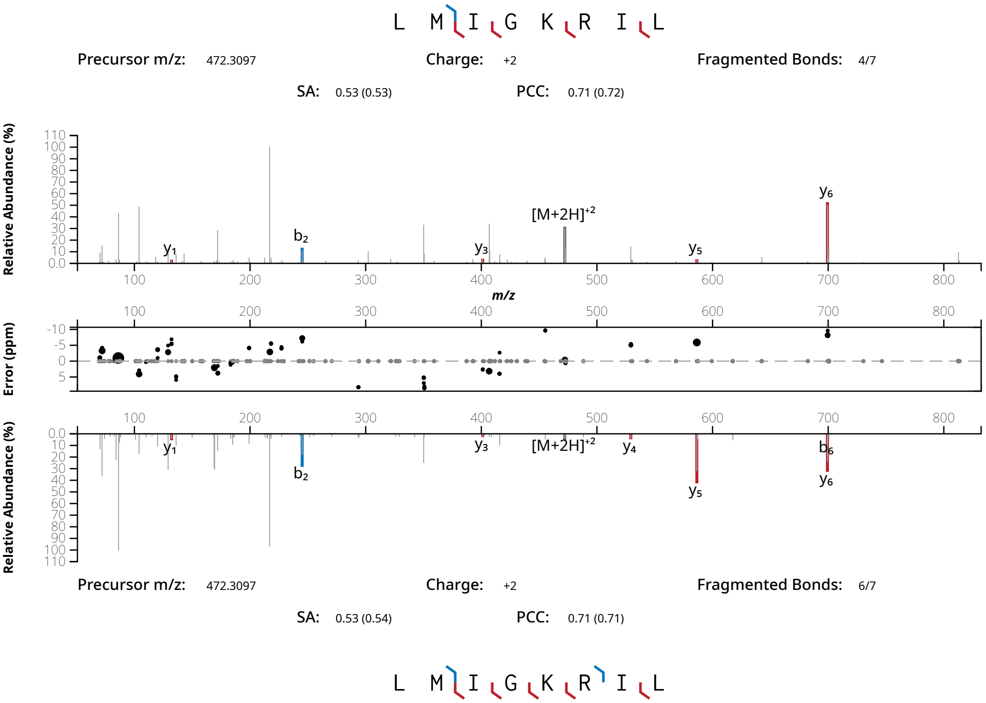
L M I G K R I L
Precursor m/z: 472.3097
Charge: +2
Fragmented Bonds: 4/7
SA: 0.53 (0.53)
PCC: 0.71 (0.72)
Relative Abundance (%)
m/z
Error (ppm)
Relative Abundance (%)
Precursor m/z: 472.3097
Charge: +2
Fragmented Bonds: 6/7
SA: 0.53 (0.54)
PCC: 0.71 (0.71)
L M I G K R I L

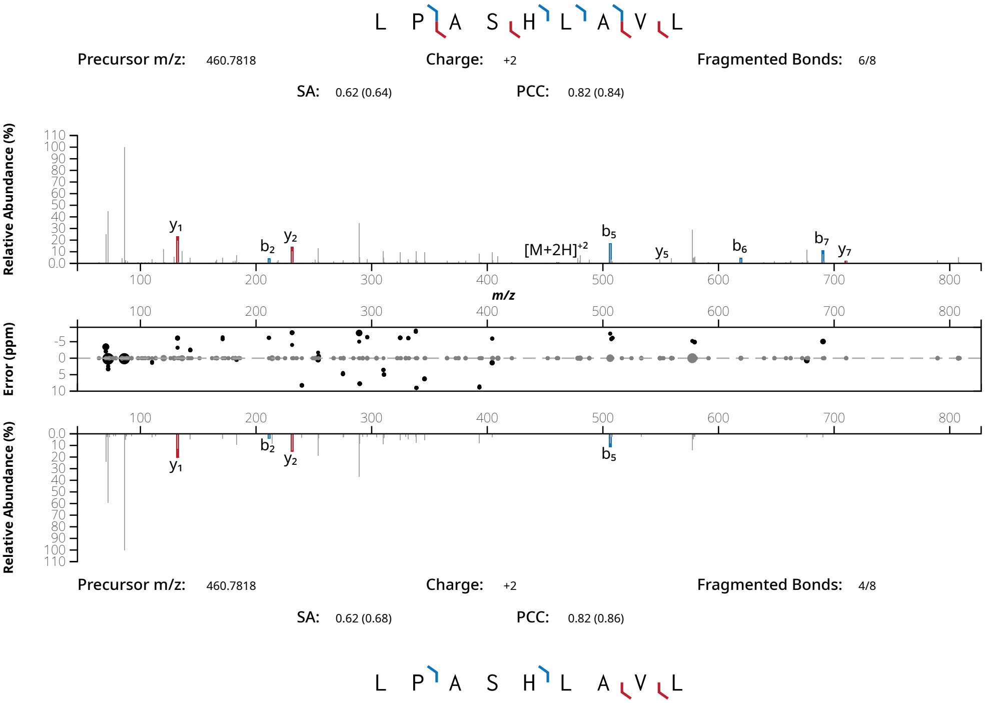

L P A S H L A V L
Precursor m/z: 460.7818
Charge: +2
Fragmented Bonds: 6/8
SA: 0.62 (0.64)
PCC: 0.82 (0.84)
Relative Abundance (%)
y1 b2 y2 [M+2H]+2 b5 y5 b6 b7 y7
m/z
Error (ppm)
Relative Abundance (%)
y1 b2 y2 b5
Precursor m/z: 460.7818
Charge: +2
Fragmented Bonds: 4/8
SA: 0.62 (0.68)
PCC: 0.82 (0.86)
L P A S H L A V L

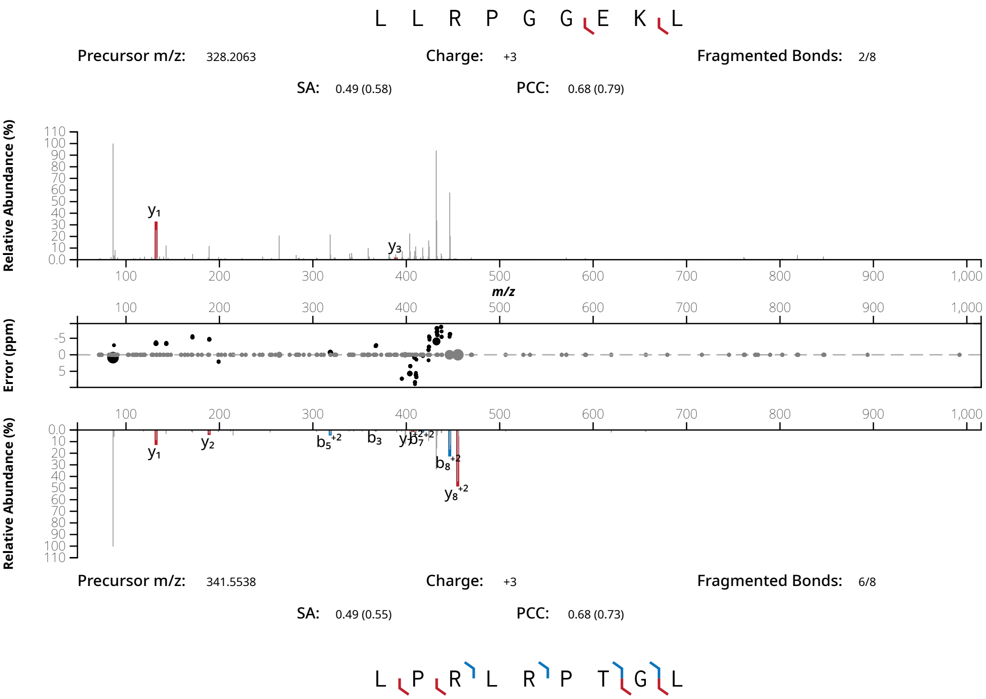

L L R P G G E K L
Precursor m/z: 328.2063
Charge: +3
Fragmented Bonds: 2/8
SA: 0.49 (0.58)
PCC: 0.68 (0.79)
Relative Abundance (%)
m/z
Error (ppm)
Relative Abundance (%)
Precursor m/z: 341.5538
Charge: +3
Fragmented Bonds: 6/8
SA: 0.49 (0.55)
PCC: 0.68 (0.73)
L P R L R P T G L

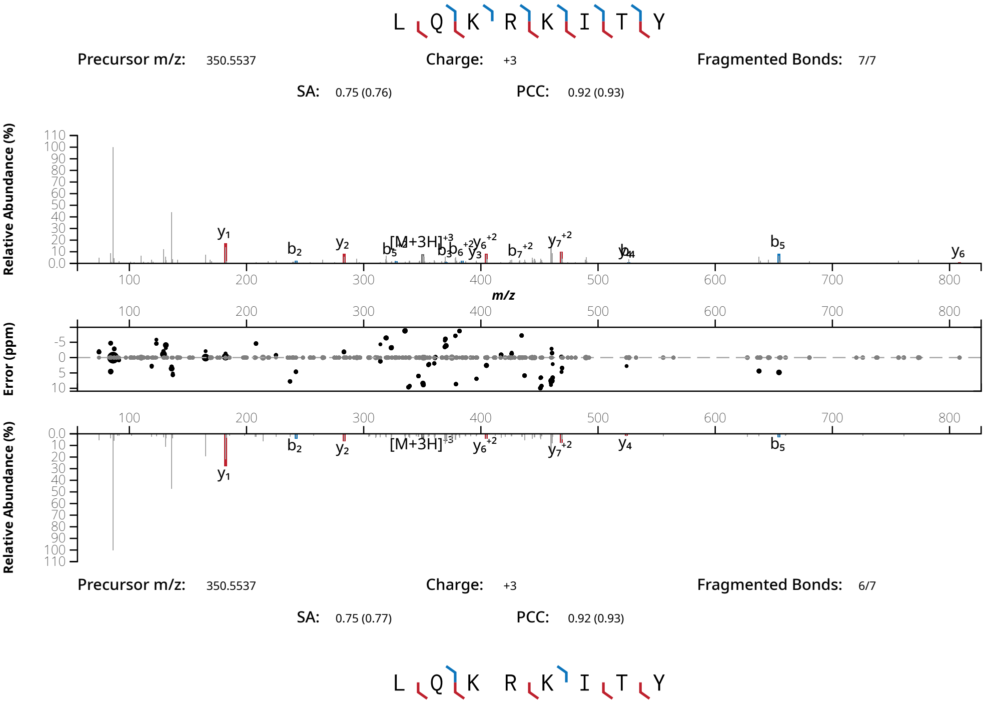
L Q K R K I T Y
Precursor m/z: 350.5537
Charge: +3
Fragmented Bonds: 7/7
SA: 0.75 (0.76)
PCC: 0.92 (0.93)
Relative Abundance (%)
m/z
Error (ppm)
Relative Abundance (%)
Precursor m/z: 350.5537
Charge: +3
Fragmented Bonds: 6/7
SA: 0.75 (0.77)
PCC: 0.92 (0.93)
L Q K R K I T Y

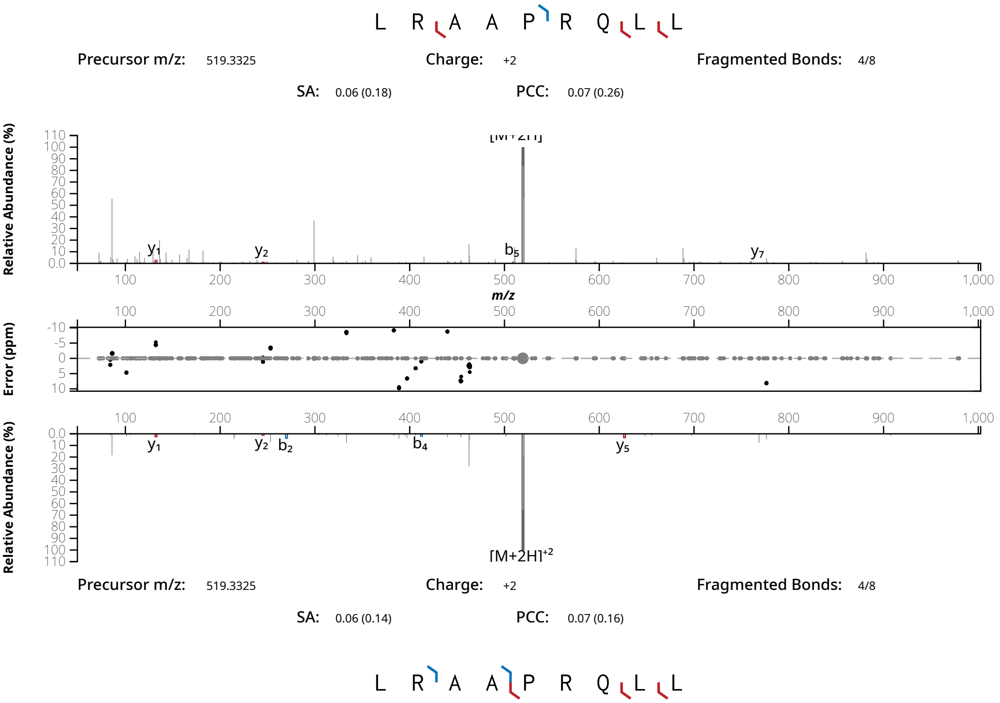
L R A A P R Q L L L
Precursor m/z: 519.3325
Charge: +2
Fragmented Bonds: 4/8
SA: 0.06 (0.18)
PCC: 0.07 (0.26)
Relative Abundance (%)
y1
y2
b5
y7
m/z
Error (ppm)
Relative Abundance (%)
y1
y2
b2
b4
y5
[M+2H]+2
Precursor m/z: 519.3325
Charge: +2
Fragmented Bonds: 4/8
SA: 0.06 (0.14)
PCC: 0.07 (0.16)
L R A A P R Q L L L

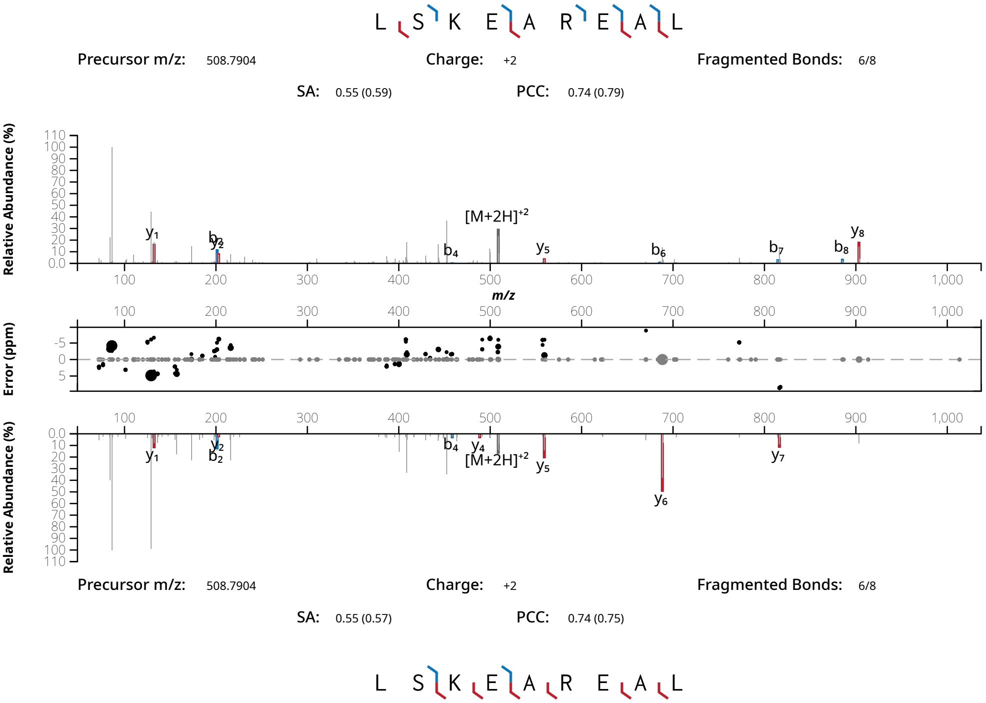
L S K E A R E A L
Precursor m/z: 508.7904
Charge: +2
Fragmented Bonds: 6/8
SA: 0.55 (0.59)
PCC: 0.74 (0.79)
Relative Abundance (%)
[M+2H]+2
y1
b2
y2
b4
y5
b6
b7
b8
y8
m/z
Error (ppm)
Relative Abundance (%)
y1
y2
b2
b4
y4
[M+2H]+2
y5
y6
y7
Precursor m/z: 508.7904
Charge: +2
Fragmented Bonds: 6/8
SA: 0.55 (0.57)
PCC: 0.74 (0.75)
L S K E A R E A L

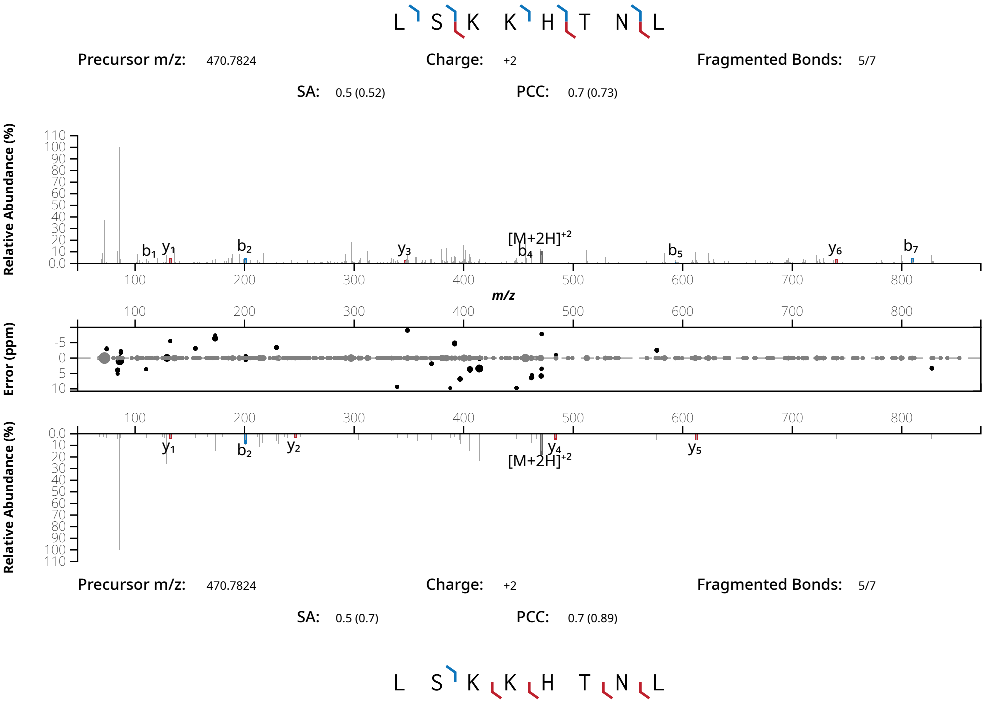
L S K K H T N L
Precursor m/z: 470.7824
Charge: +2
Fragmented Bonds: 5/7
SA: 0.5 (0.52)
PCC: 0.7 (0.73)
Relative Abundance (%)
m/z
Error (ppm)
Relative Abundance (%)
Precursor m/z: 470.7824
Charge: +2
Fragmented Bonds: 5/7
SA: 0.5 (0.7)
PCC: 0.7 (0.89)
L S K K H T N L

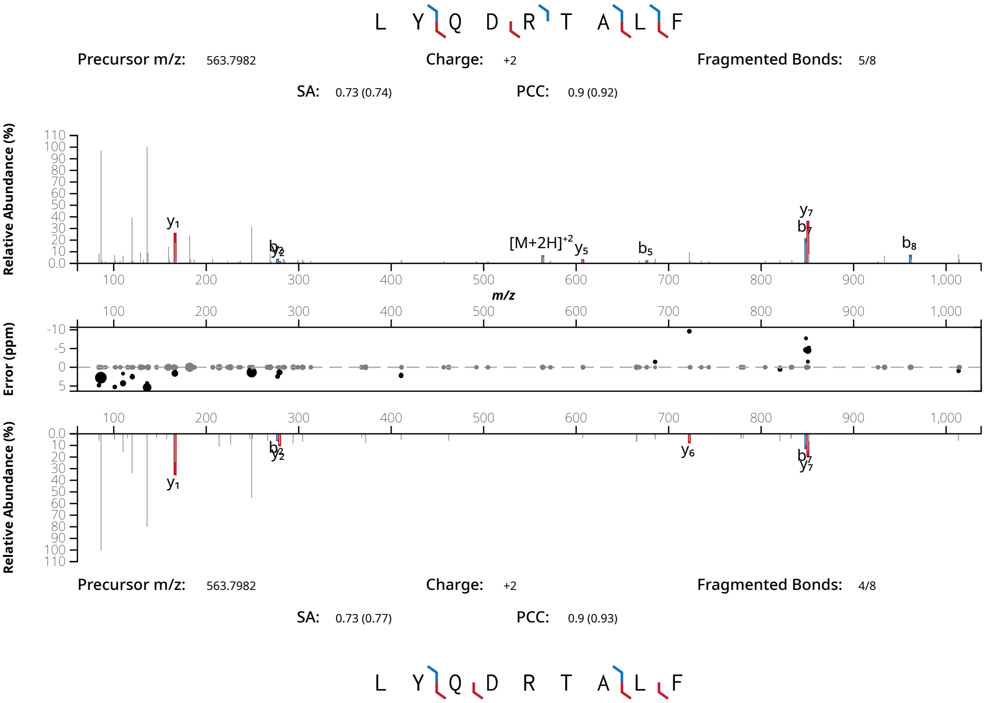
L Y Q D R T A L F
Precursor m/z: 563.7982
Charge: +2
Fragmented Bonds: 5/8
SA: 0.73 (0.74)
PCC: 0.9 (0.92)
Relative Abundance (%)
y1
b2
y2
[M+2H]+2
y5
b5
y7
b7
b8
m/z
Error (ppm)
Relative Abundance (%)
y1
b2
y2
y6
b7
y7
Precursor m/z: 563.7982
Charge: +2
Fragmented Bonds: 4/8
SA: 0.73 (0.77)
PCC: 0.9 (0.93)
L Y Q D R T A L F

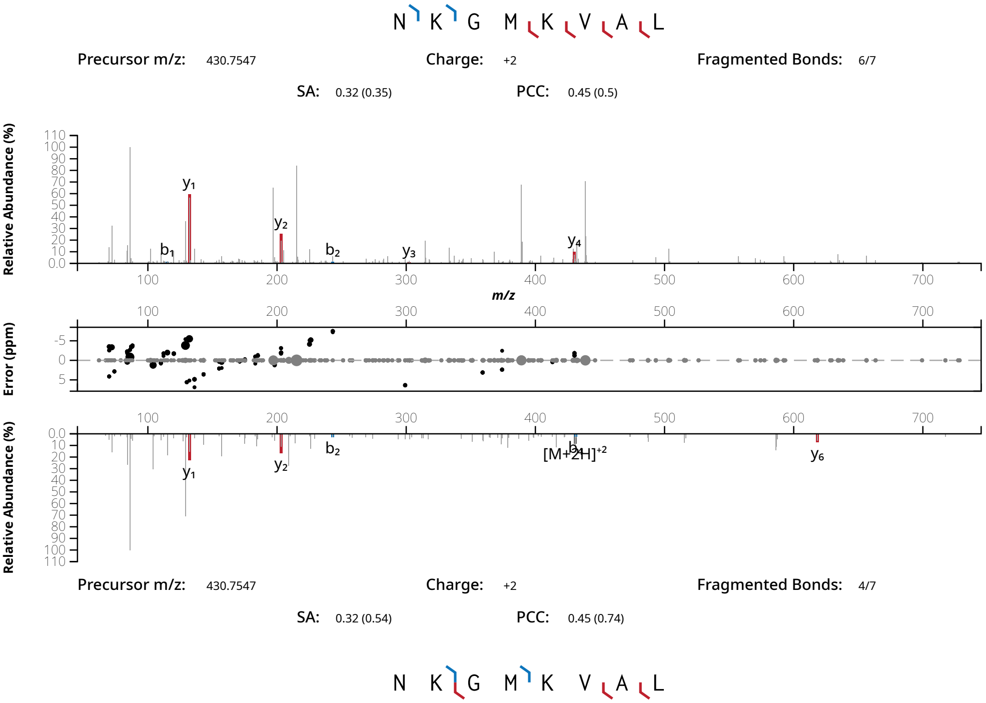

N K G M K V A L
Precursor m/z: 430.7547
Charge: +2
Fragmented Bonds: 6/7
SA: 0.32 (0.35)
PCC: 0.45 (0.5)
Relative Abundance (%)
y1
y2
b1
b2
y3
y4
m/z
Error (ppm)
Relative Abundance (%)
y1
y2
b2
b4
[M+2H]+2
y6
Precursor m/z: 430.7547
Charge: +2
Fragmented Bonds: 4/7
SA: 0.32 (0.54)
PCC: 0.45 (0.74)
N K G M K V A L

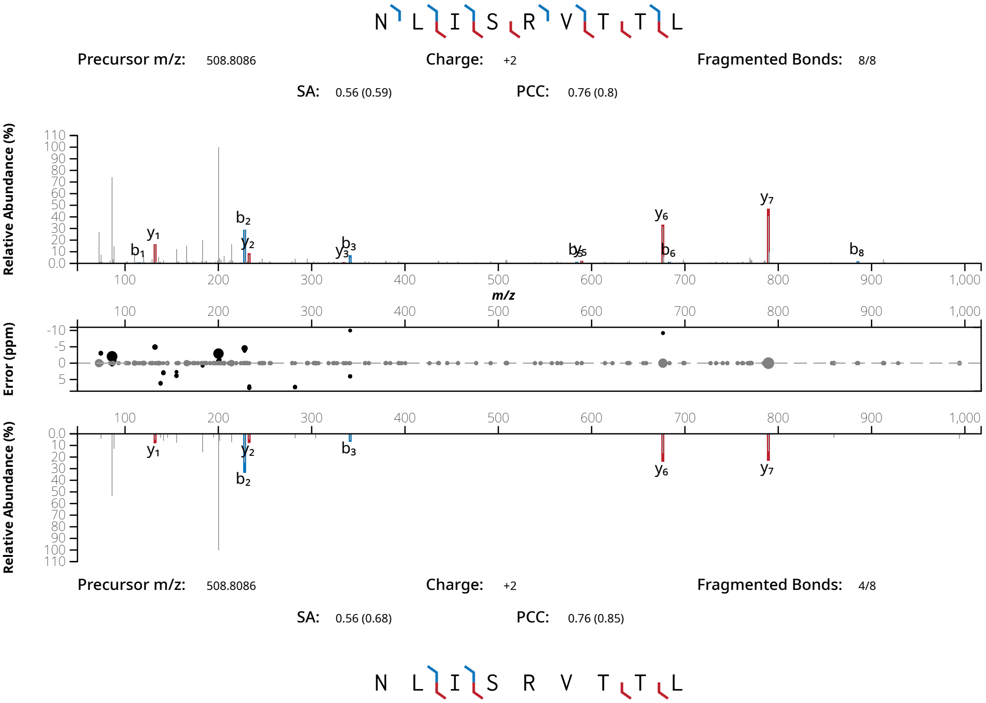
N L I S R V T T L
Precursor m/z: 508.8086
Charge: +2
Fragmented Bonds: 8/8
SA: 0.56 (0.59)
PCC: 0.76 (0.8)
Relative Abundance (%)
m/z
Error (ppm)
Relative Abundance (%)
Precursor m/z: 508.8086
Charge: +2
Fragmented Bonds: 4/8
SA: 0.56 (0.68)
PCC: 0.76 (0.85)
N L I S R V T T L

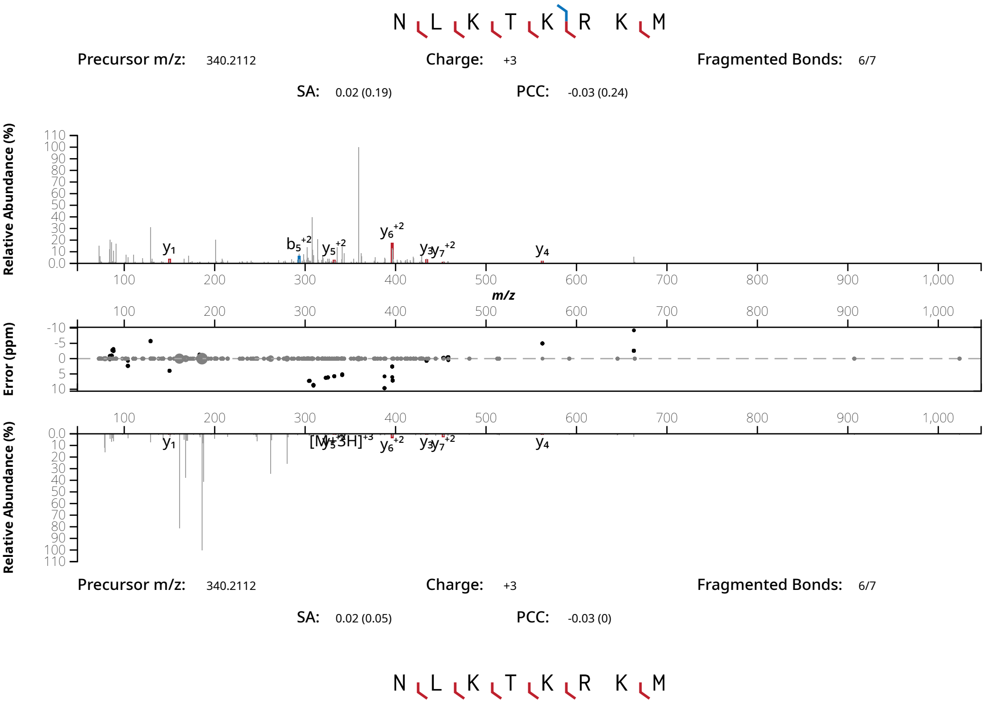
N L K T K R K M
Precursor m/z: 340.2112
Charge: +3
Fragmented Bonds: 6/7
SA: 0.02 (0.19)
PCC: -0.03 (0.24)
Relative Abundance (%)
m/z
Error (ppm)
Relative Abundance (%)
Precursor m/z: 340.2112
Charge: +3
Fragmented Bonds: 6/7
SA: 0.02 (0.05)
PCC: -0.03 (0)
N L K T K R K M

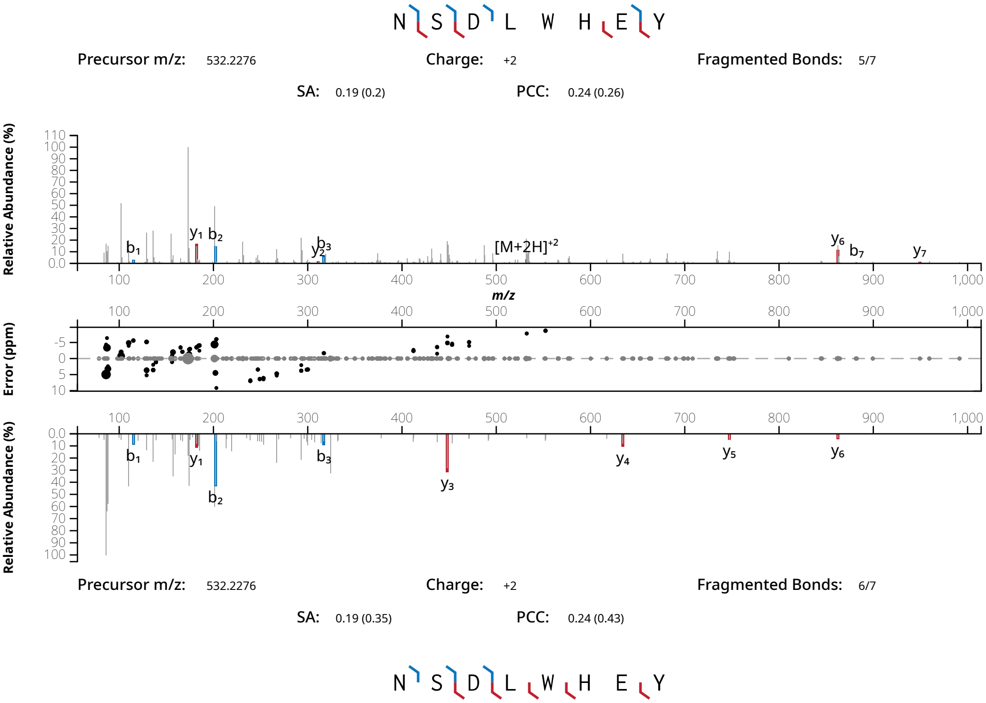

N S D L W H E Y
Precursor m/z: 532.2276
Charge: +2
Fragmented Bonds: 5/7
SA: 0.19 (0.2)
PCC: 0.24 (0.26)
Relative Abundance (%)
m/z
Error (ppm)
Relative Abundance (%)
Precursor m/z: 532.2276
Charge: +2
Fragmented Bonds: 6/7
SA: 0.19 (0.35)
PCC: 0.24 (0.43)
N S D L W H E Y

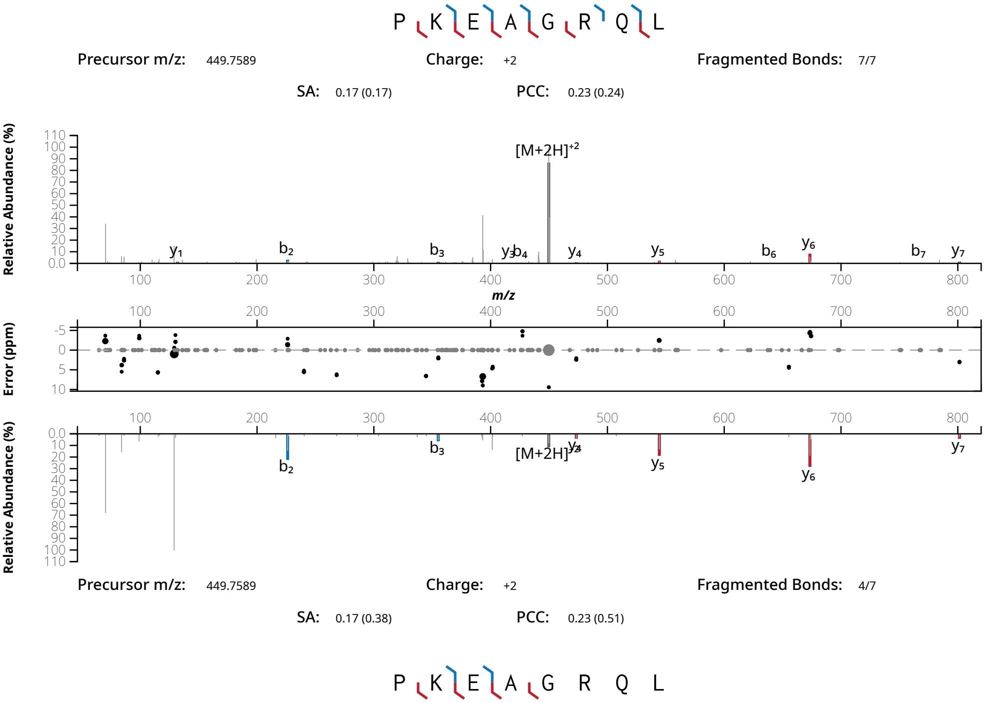
P K E A G R Q L
Precursor m/z: 449.7589
Charge: +2
Fragmented Bonds: 7/7
SA: 0.17 (0.17)
PCC: 0.23 (0.24)
Relative Abundance (%)
[M+2H]+2
y1 b2 b3 y3 b4 y4 y5 b6 y6 b7 y7
m/z
Error (ppm)
Relative Abundance (%)
b2 b3 [M+2H]+2 y4 y5 y6 y7
Precursor m/z: 449.7589
Charge: +2
Fragmented Bonds: 4/7
SA: 0.17 (0.38)
PCC: 0.23 (0.51)
P K E A G R Q L

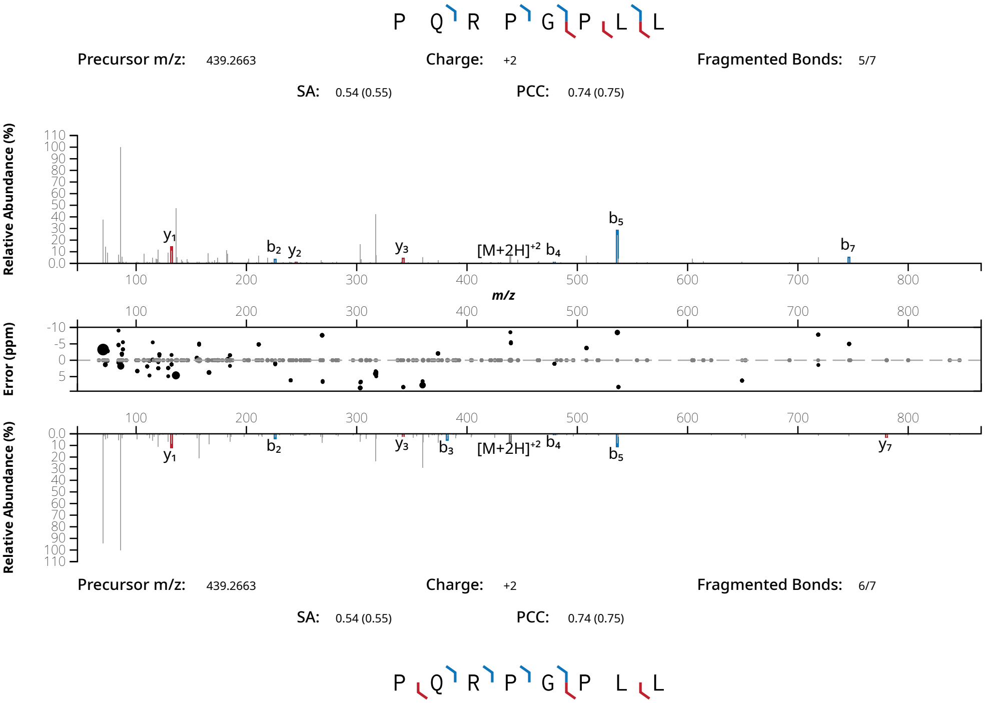
P Q R P G P L L
Precursor m/z: 439.2663
Charge: +2
Fragmented Bonds: 5/7
SA: 0.54 (0.55)
PCC: 0.74 (0.75)
Relative Abundance (%)
y1
b2
y2
y3
[M+2H]+2
b4
b5
b7
m/z
Error (ppm)
b3
y7
Precursor m/z: 439.2663
Charge: +2
Fragmented Bonds: 6/7
SA: 0.54 (0.55)
PCC: 0.74 (0.75)
P Q R P G P L L

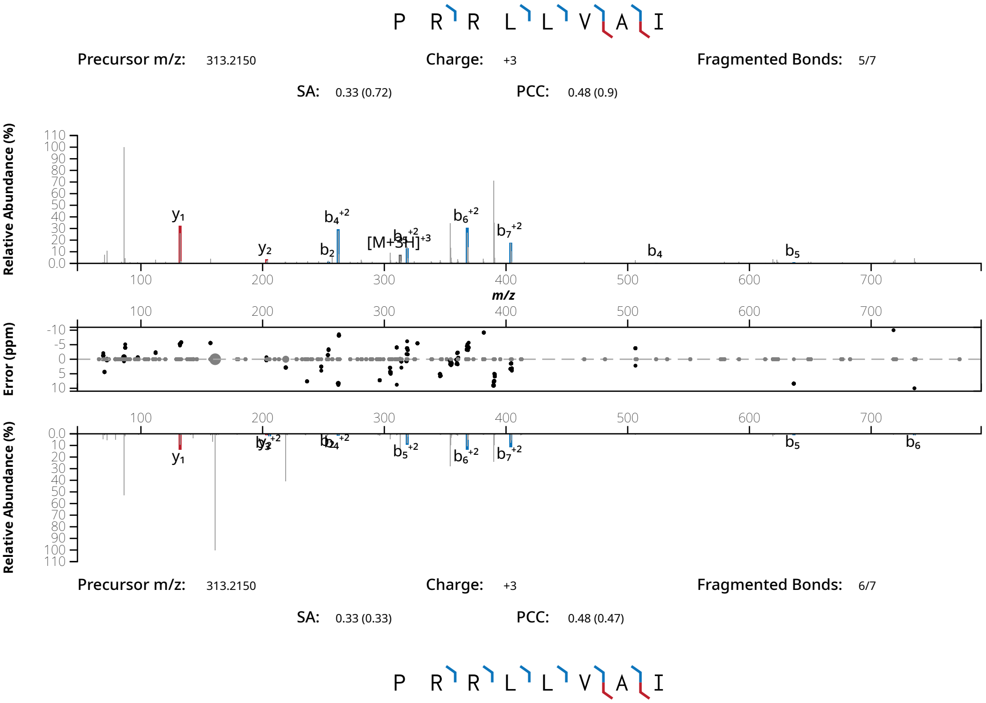
P R R L L V A I
Precursor m/z: 313.2150
Charge: +3
Fragmented Bonds: 5/7
SA: 0.33 (0.72)
PCC: 0.48 (0.9)
Relative Abundance (%)
m/z
Error (ppm)
Relative Abundance (%)
Precursor m/z: 313.2150
Charge: +3
Fragmented Bonds: 6/7
SA: 0.33 (0.33)
PCC: 0.48 (0.47)
P R R L L V A I

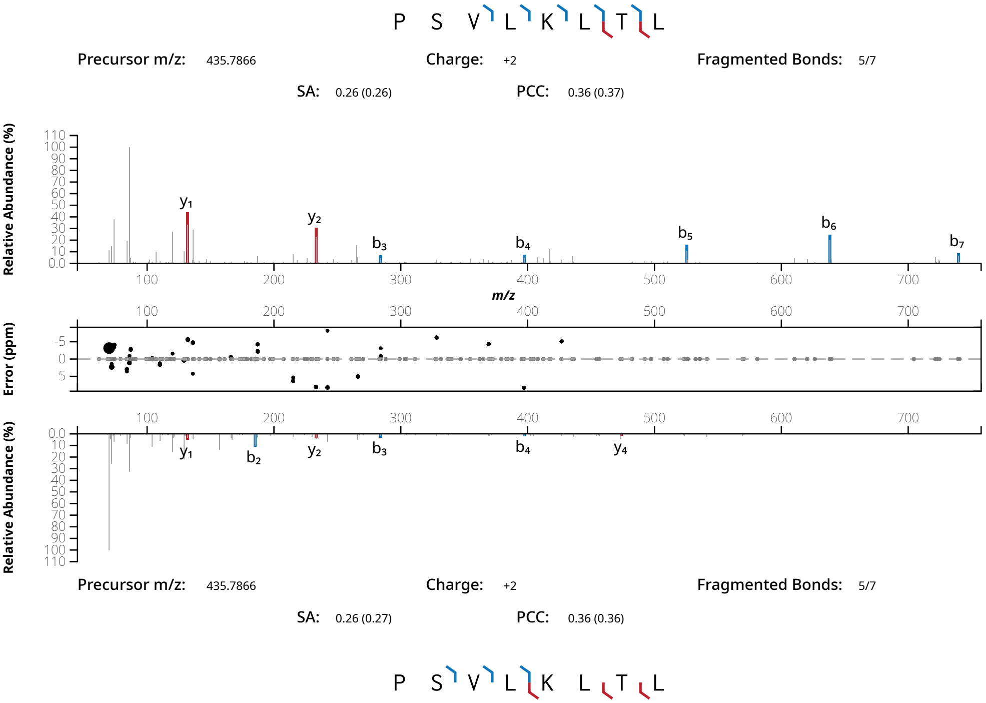
P S V L K L T L
Precursor m/z: 435.7866
Charge: +2
Fragmented Bonds: 5/7
SA: 0.26 (0.26)
PCC: 0.36 (0.37)
Relative Abundance (%)
Error (ppm)
m/z
Precursor m/z: 435.7866
Charge: +2
Fragmented Bonds: 5/7
SA: 0.26 (0.27)
PCC: 0.36 (0.36)
P S V L K L T L

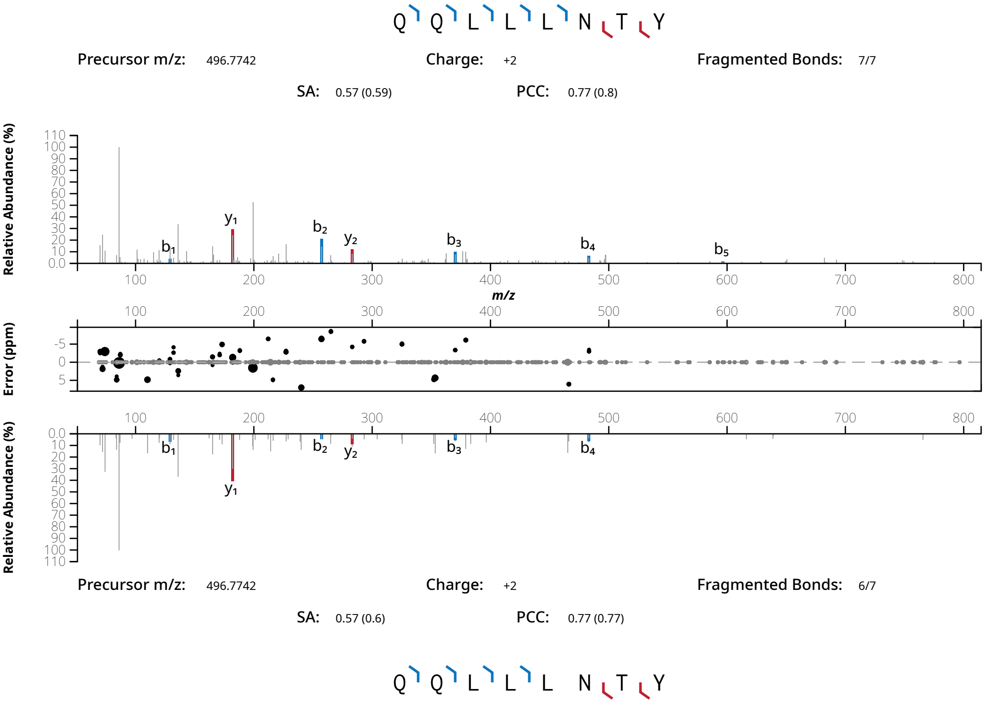
Q Q L L L N T Y
Precursor m/z: 496.7742
Charge: +2
Fragmented Bonds: 7/7
SA: 0.57 (0.59)
PCC: 0.77 (0.8)
Relative Abundance (%)
y1
b1
b2
y2
b3
b4
b5
m/z
Error (ppm)
Relative Abundance (%)
Precursor m/z: 496.7742
Charge: +2
Fragmented Bonds: 6/7
SA: 0.57 (0.6)
PCC: 0.77 (0.77)
Q Q L L L N T Y

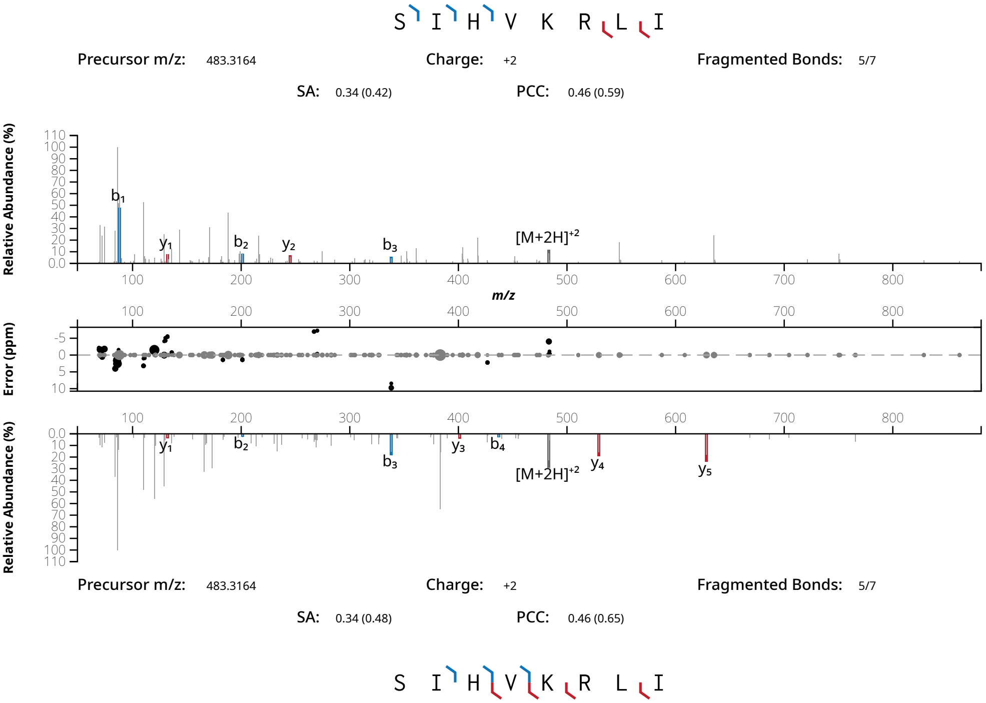
S I H V K R L I
Precursor m/z: 483.3164
Charge: +2
Fragmented Bonds: 5/7
SA: 0.34 (0.42)
PCC: 0.46 (0.59)
Relative Abundance (%)
b1
y1
b2
y2
b3
[M+2H]+2
m/z
Error (ppm)
Relative Abundance (%)
y1
b2
b3
y3
b4
[M+2H]+2
y4
y5
Precursor m/z: 483.3164
Charge: +2
Fragmented Bonds: 5/7
SA: 0.34 (0.48)
PCC: 0.46 (0.65)
S I H V K R L I

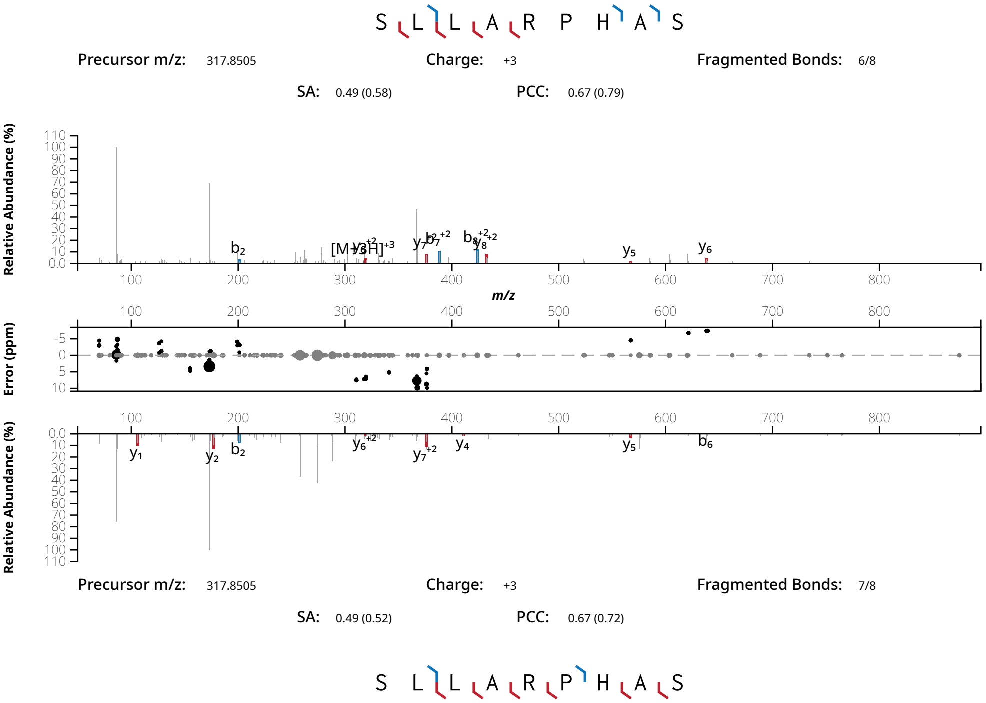
S L L A R P H A S
Precursor m/z: 317.8505
Charge: +3
Fragmented Bonds: 6/8
SA: 0.49 (0.58)
PCC: 0.67 (0.79)
Relative Abundance (%)
m/z
Error (ppm)
Relative Abundance (%)
Precursor m/z: 317.8505
Charge: +3
Fragmented Bonds: 7/8
SA: 0.49 (0.52)
PCC: 0.67 (0.72)
S L L A R P H A S

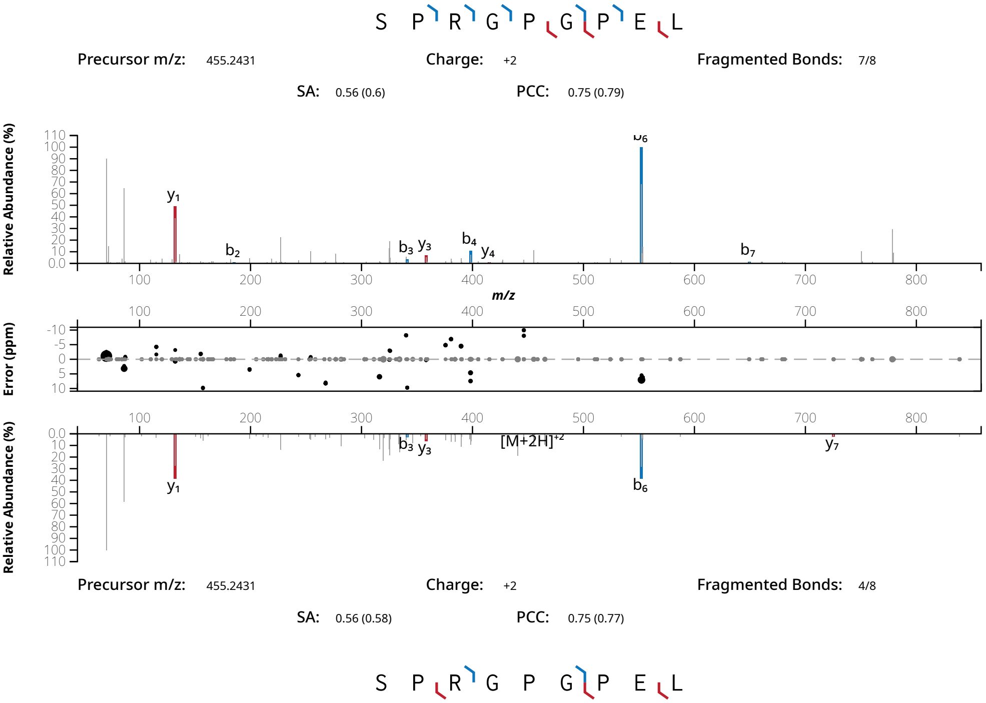
S P R G P G P E L
Precursor m/z: 455.2431
Charge: +2
Fragmented Bonds: 7/8
SA: 0.56 (0.6)
PCC: 0.75 (0.79)
Relative Abundance (%)
y1
b2
b3
y3
b4
y4
b6
b7
m/z
Error (ppm)
Relative Abundance (%)
b3
y3
[M+2H]+2
y7
y1
b6
Precursor m/z: 455.2431
Charge: +2
Fragmented Bonds: 4/8
SA: 0.56 (0.58)
PCC: 0.75 (0.77)
S P R G P G P E L

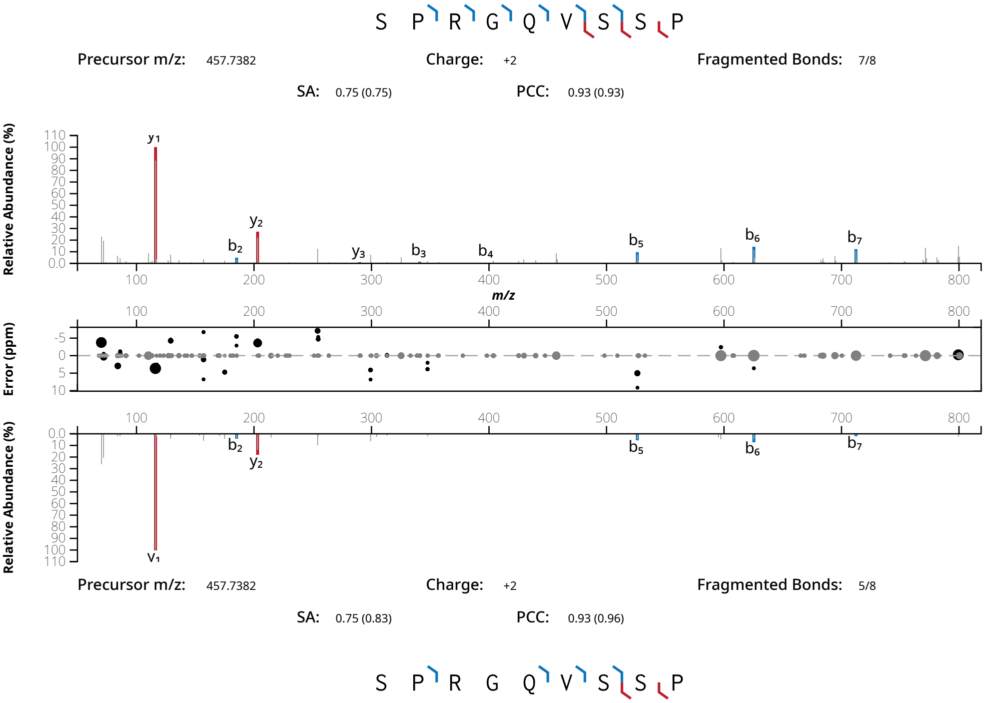
S P R G Q V S S P
Precursor m/z: 457.7382
Charge: +2
Fragmented Bonds: 7/8
SA: 0.75 (0.75)
PCC: 0.93 (0.93)
Relative Abundance (%)
Error (ppm)
m/z
Precursor m/z: 457.7382
Charge: +2
Fragmented Bonds: 5/8
SA: 0.75 (0.83)
PCC: 0.93 (0.96)
S P R G Q V S S P

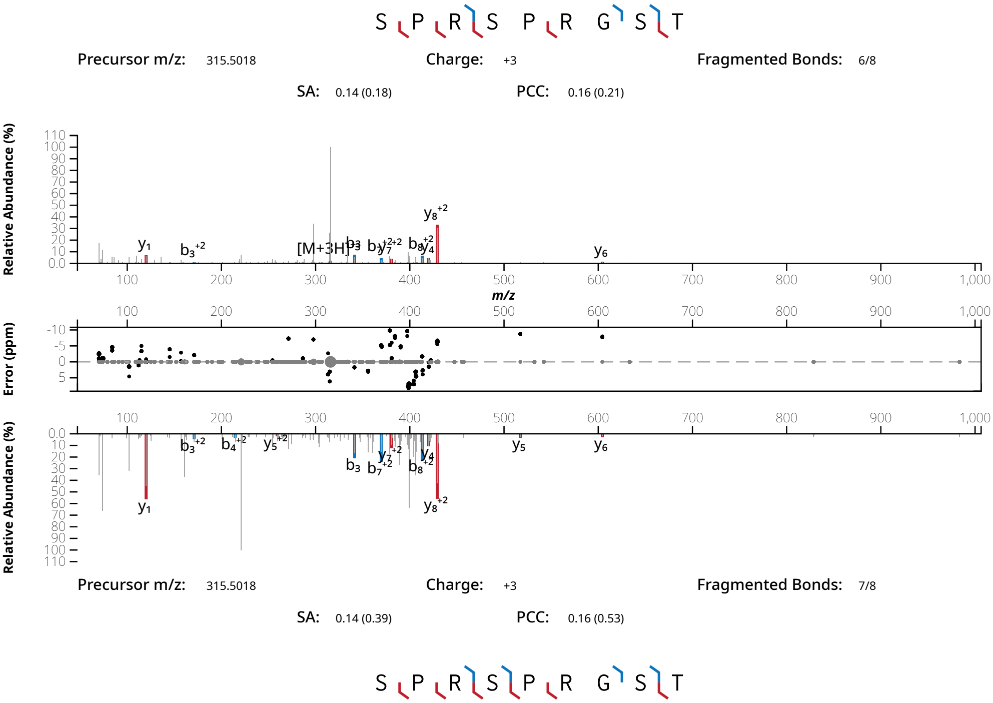

S P R S P R G S T
Precursor m/z: 315.5018
Charge: +3
Fragmented Bonds: 6/8
SA: 0.14 (0.18)
PCC: 0.16 (0.21)
Relative Abundance (%)
m/z
Error (ppm)
Relative Abundance (%)
Precursor m/z: 315.5018
Charge: +3
Fragmented Bonds: 7/8
SA: 0.14 (0.39)
PCC: 0.16 (0.53)
S P R S P R G S T

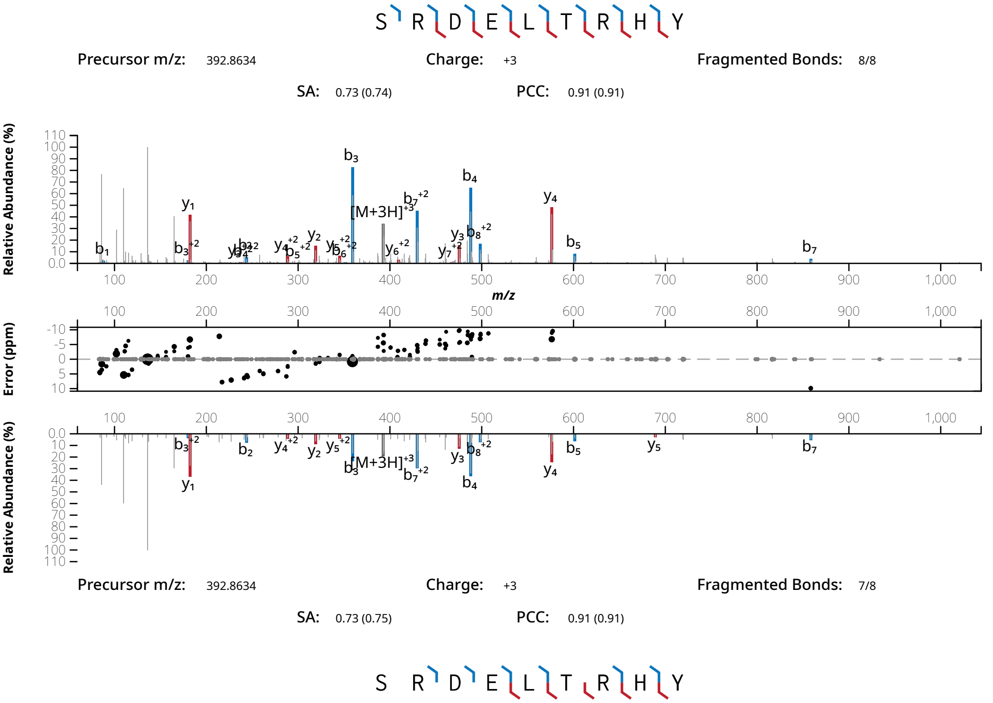
S R D E L T R H Y
Precursor m/z: 392.8634
Charge: +3
Fragmented Bonds: 8/8
SA: 0.73 (0.74)
PCC: 0.91 (0.91)
Relative Abundance (%)
m/z
Error (ppm)
Relative Abundance (%)
Precursor m/z: 392.8634
Charge: +3
Fragmented Bonds: 7/8
SA: 0.73 (0.75)
PCC: 0.91 (0.91)
S R D E L T R H Y

S S A P P L L L

Precursor m/z: 797.4767 Charge: +1 Fragmented Bonds: 0/7

SA: 0.76 (0.78) PCC: 0.92 (0.94)

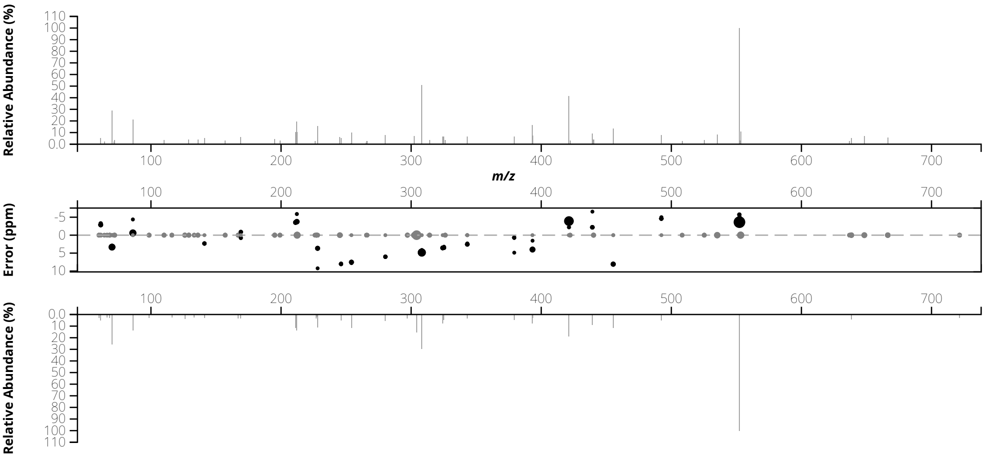

Precursor m/z: 797.4767 Charge: +1 Fragmented Bonds: 0/7

SA: 0.76 (0.79) PCC: 0.92 (0.94)

S S A P P L L L

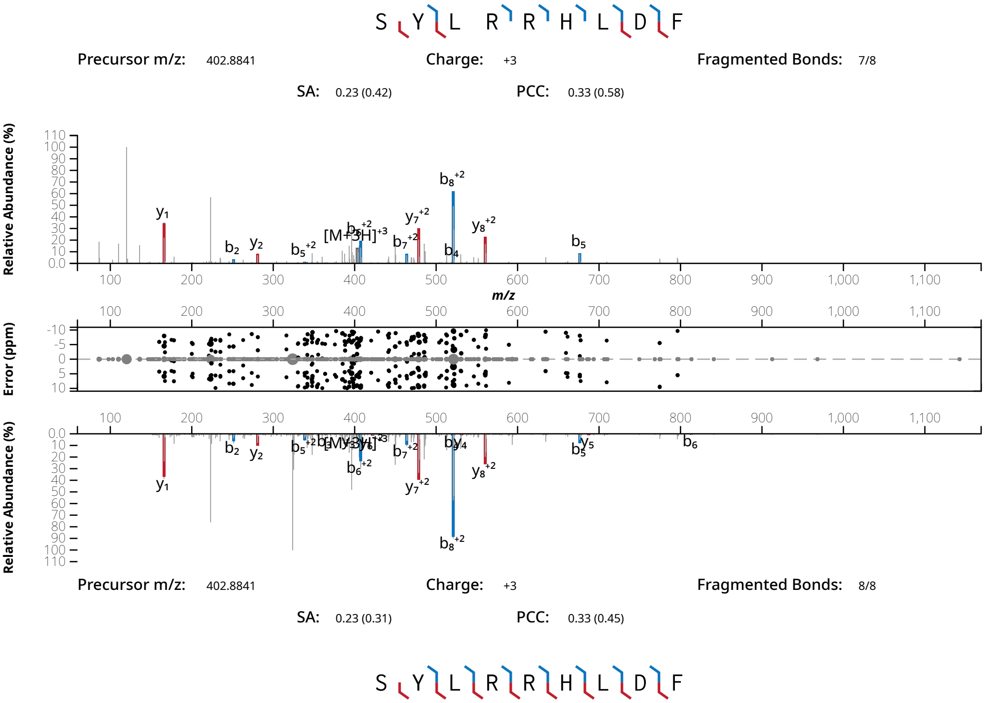
S Y L R R H L D F
Precursor m/z: 402.8841
Charge: +3
Fragmented Bonds: 7/8
SA: 0.23 (0.42)
PCC: 0.33 (0.58)
Relative Abundance (%)
m/z
Error (ppm)
Relative Abundance (%)
Precursor m/z: 402.8841
Charge: +3
Fragmented Bonds: 8/8
SA: 0.23 (0.31)
PCC: 0.33 (0.45)
S Y L R R H L D F

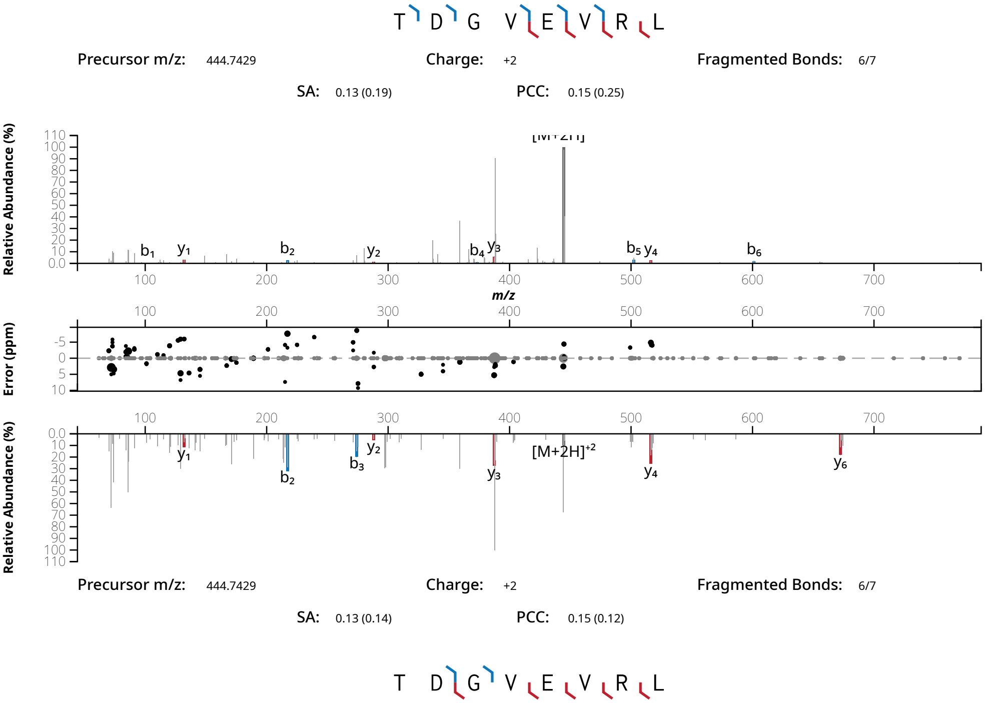
T D G V E V R L
Precursor m/z: 444.7429
Charge: +2
Fragmented Bonds: 6/7
SA: 0.13 (0.19)
PCC: 0.15 (0.25)
Relative Abundance (%)
m/z
Error (ppm)
Relative Abundance (%)
[M+2H]+2
Precursor m/z: 444.7429
Charge: +2
Fragmented Bonds: 6/7
SA: 0.13 (0.14)
PCC: 0.15 (0.12)
T D G V E V R L

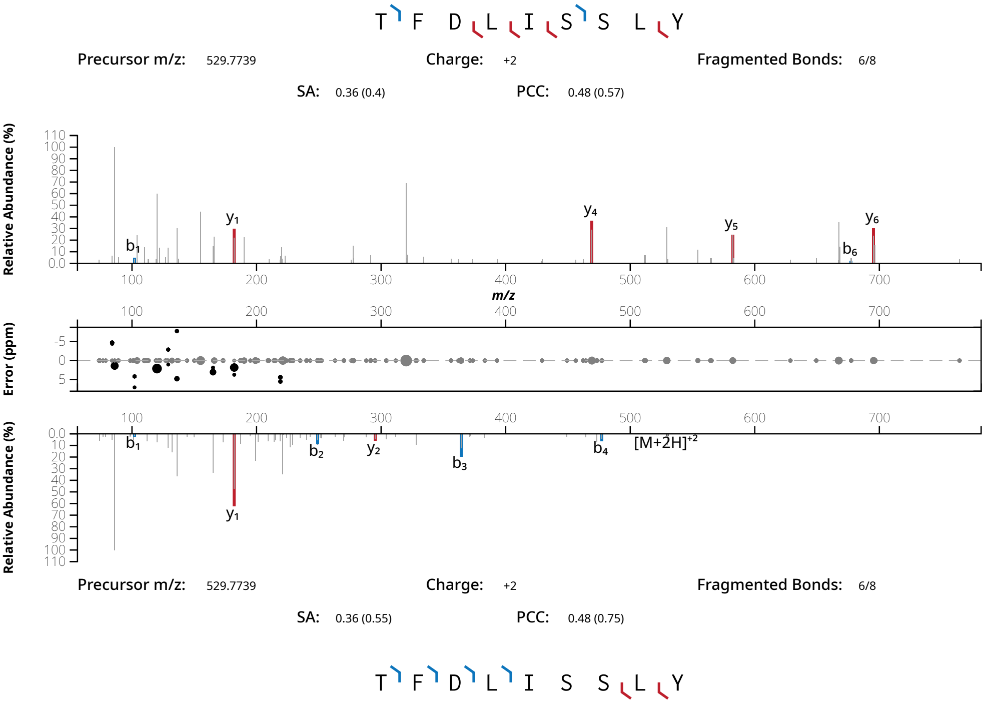
T F D L I S S L Y
Precursor m/z: 529.7739
Charge: +2
Fragmented Bonds: 6/8
SA: 0.36 (0.4)
PCC: 0.48 (0.57)
Relative Abundance (%)
b1
y1
y4
y5
b6
y6
m/z
Error (ppm)
Relative Abundance (%)
b1
b2
y2
b3
b4
[M+2H]+2
y1
Precursor m/z: 529.7739
Charge: +2
Fragmented Bonds: 6/8
SA: 0.36 (0.55)
PCC: 0.48 (0.75)
T F D L I S S L Y

T F Q K K T K E M

Precursor m/z: 570.8077 Charge: +2 Fragmented Bonds: 3/8

SA: 0.32 (0.49) PCC: 0.44 (0.7)

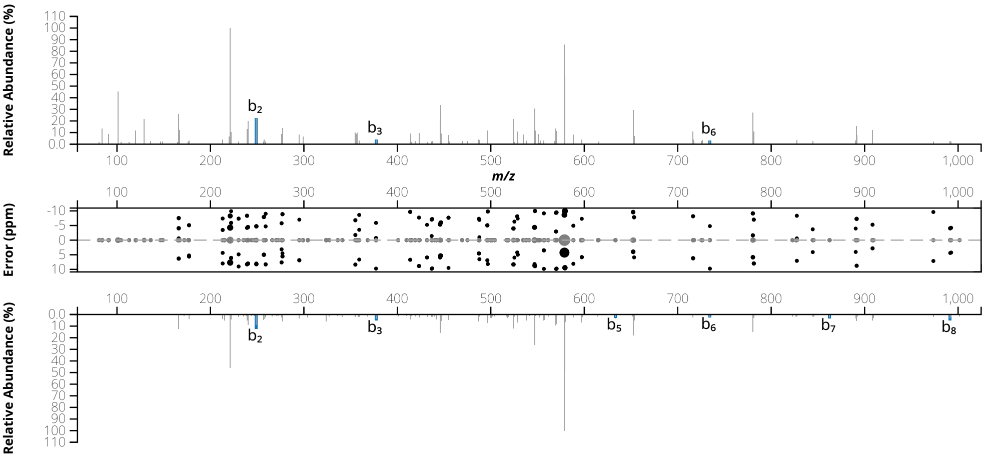

Precursor m/z: 570.8077 Charge: +2 Fragmented Bonds: 6/8

SA: 0.32 (0.36) PCC: 0.44 (0.49)

T F Q K K T K E M

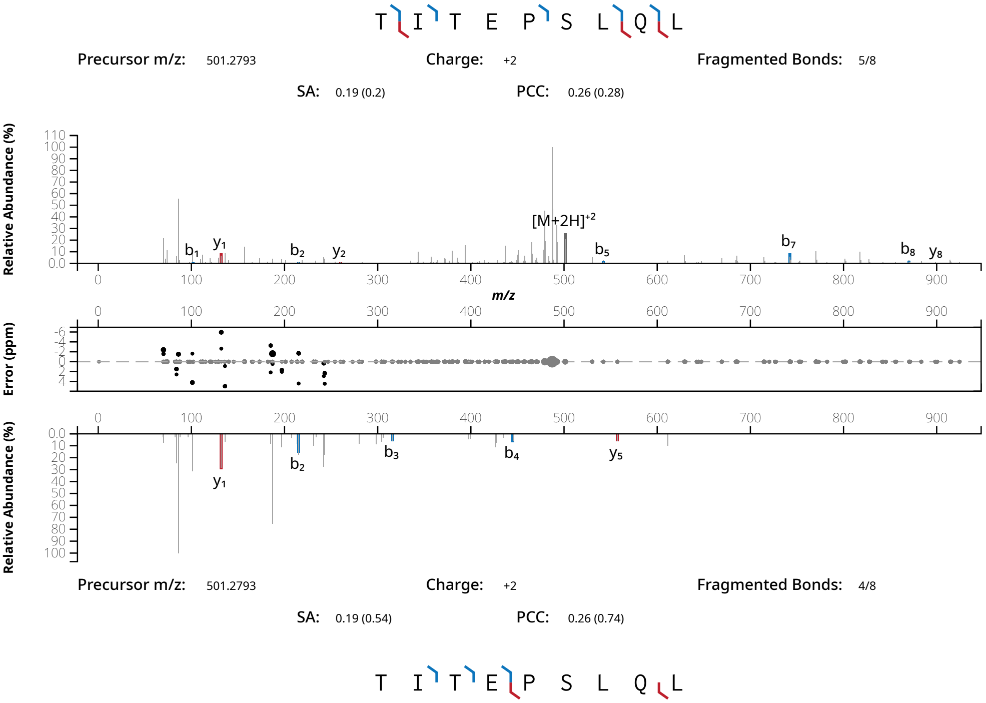
T I T E P S L Q L
Precursor m/z: 501.2793
Charge: +2
Fragmented Bonds: 5/8
SA: 0.19 (0.2)
PCC: 0.26 (0.28)
Relative Abundance (%)
[M+2H]+2
b1
y1
b2
y2
b5
b7
b8
y8
m/z
Error (ppm)
Relative Abundance (%)
y1
b2
b3
b4
y5
Precursor m/z: 501.2793
Charge: +2
Fragmented Bonds: 4/8
SA: 0.19 (0.54)
PCC: 0.26 (0.74)
T I T E P S L Q L

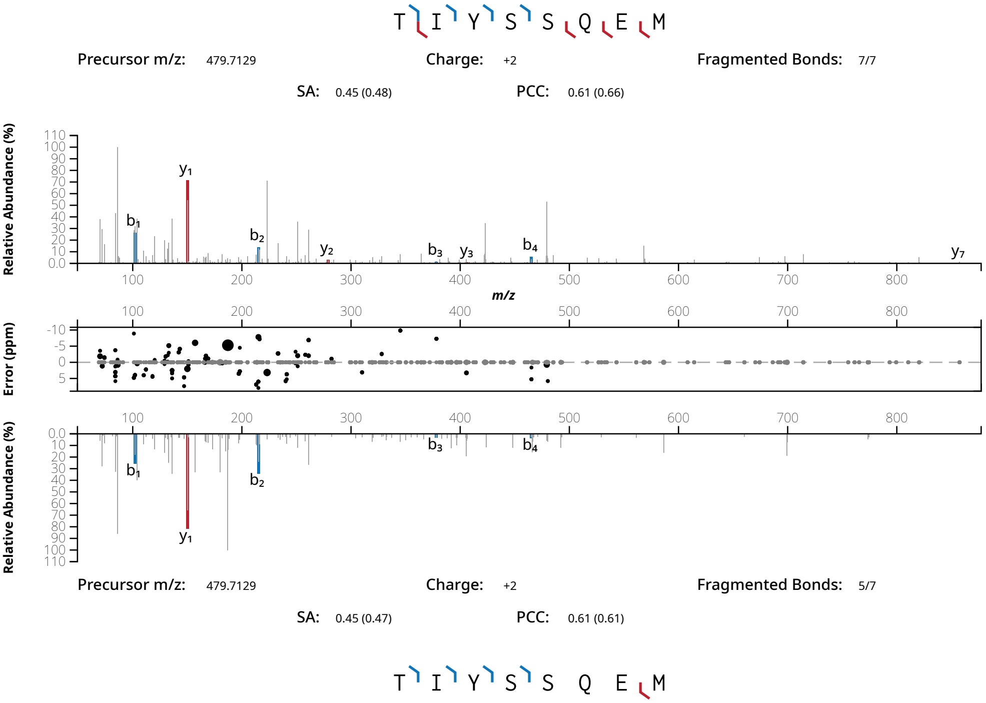
T I Y S S Q E M
Precursor m/z: 479.7129
Charge: +2
Fragmented Bonds: 7/7
SA: 0.45 (0.48)
PCC: 0.61 (0.66)
Relative Abundance (%)
Error (ppm)
m/z
Precursor m/z: 479.7129
Charge: +2
Fragmented Bonds: 5/7
SA: 0.45 (0.47)
PCC: 0.61 (0.61)
T I Y S S Q E M

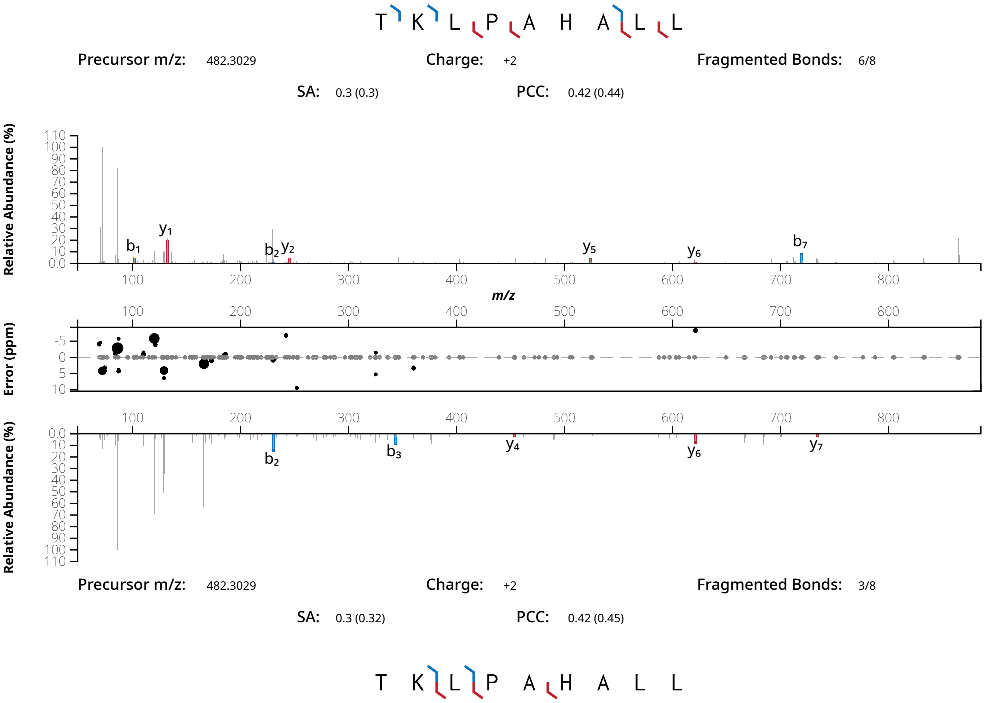
T K L P A H A L L
Precursor m/z: 482.3029
Charge: +2
Fragmented Bonds: 6/8
SA: 0.3 (0.3)
PCC: 0.42 (0.44)
Relative Abundance (%)
m/z
Error (ppm)
Relative Abundance (%)
Precursor m/z: 482.3029
Charge: +2
Fragmented Bonds: 3/8
SA: 0.3 (0.32)
PCC: 0.42 (0.45)
T K L P A H A L L

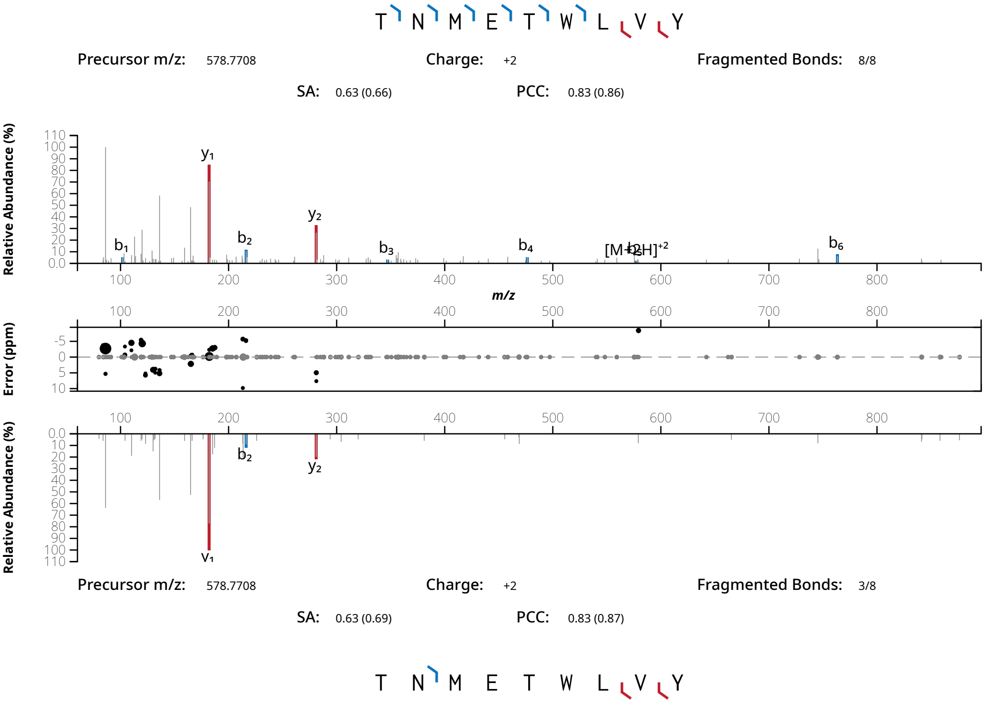
T N M E T W L V Y
Precursor m/z: 578.7708
Charge: +2
Fragmented Bonds: 8/8
SA: 0.63 (0.66)
PCC: 0.83 (0.86)
Relative Abundance (%)
y1
y2
b1
b2
b3
b4
b6
[M+2H]+2
m/z
Error (ppm)
Relative Abundance (%)
b2
y2
y1
Precursor m/z: 578.7708
Charge: +2
Fragmented Bonds: 3/8
SA: 0.63 (0.69)
PCC: 0.83 (0.87)
T N M E T W L V Y

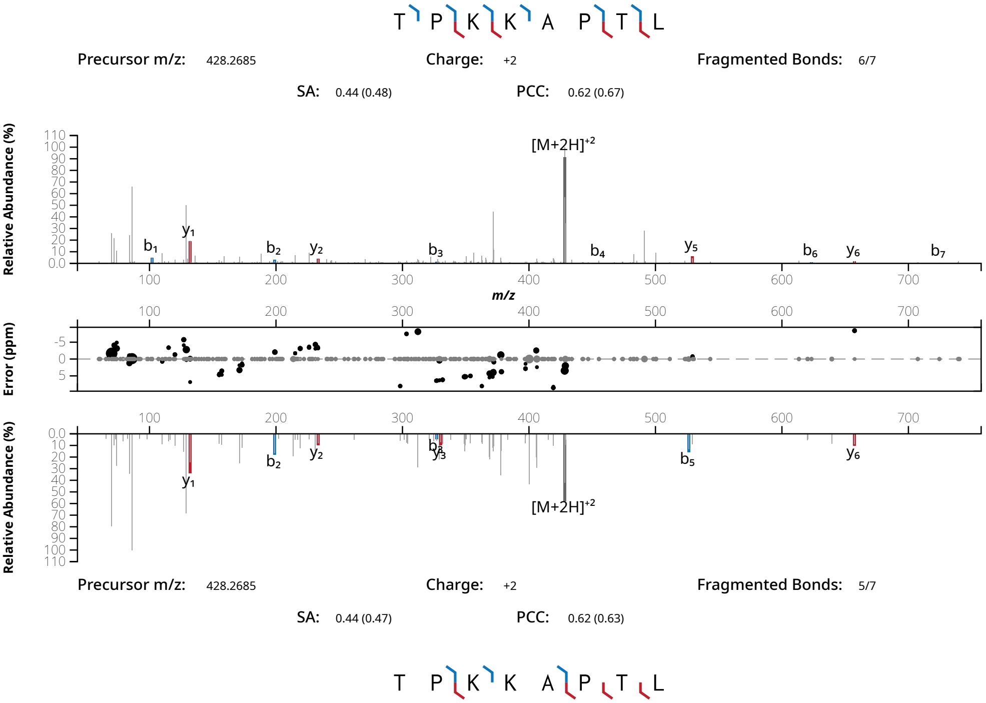
T P K K A P T L
Precursor m/z: 428.2685
Charge: +2
Fragmented Bonds: 6/7
SA: 0.44 (0.48)
PCC: 0.62 (0.67)
Relative Abundance (%)
[M+2H]+2
b1
y1
b2
y2
b3
b4
y5
b6
y6
b7
m/z
Error (ppm)
b2
y2
b3
y3
b5
y6
y1
[M+2H]+2
Relative Abundance (%)
Precursor m/z: 428.2685
Charge: +2
Fragmented Bonds: 5/7
SA: 0.44 (0.47)
PCC: 0.62 (0.63)
T P K K A P T L

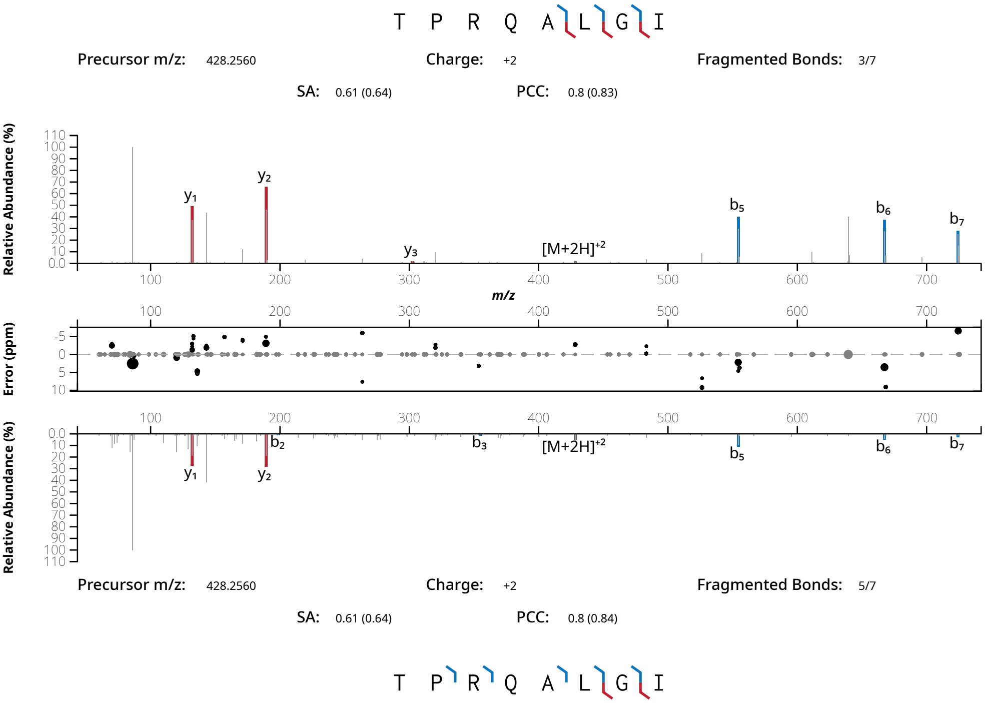

T P R Q A L G I
Precursor m/z: 428.2560
Charge: +2
Fragmented Bonds: 3/7
SA: 0.61 (0.64)
PCC: 0.8 (0.83)
Relative Abundance (%)
y1
y2
y3
[M+2H]+2
b5
b6
b7
m/z
Error (ppm)
b2
b3
Relative Abundance (%)
Precursor m/z: 428.2560
Charge: +2
Fragmented Bonds: 5/7
SA: 0.61 (0.64)
PCC: 0.8 (0.84)
T P R Q A L G I

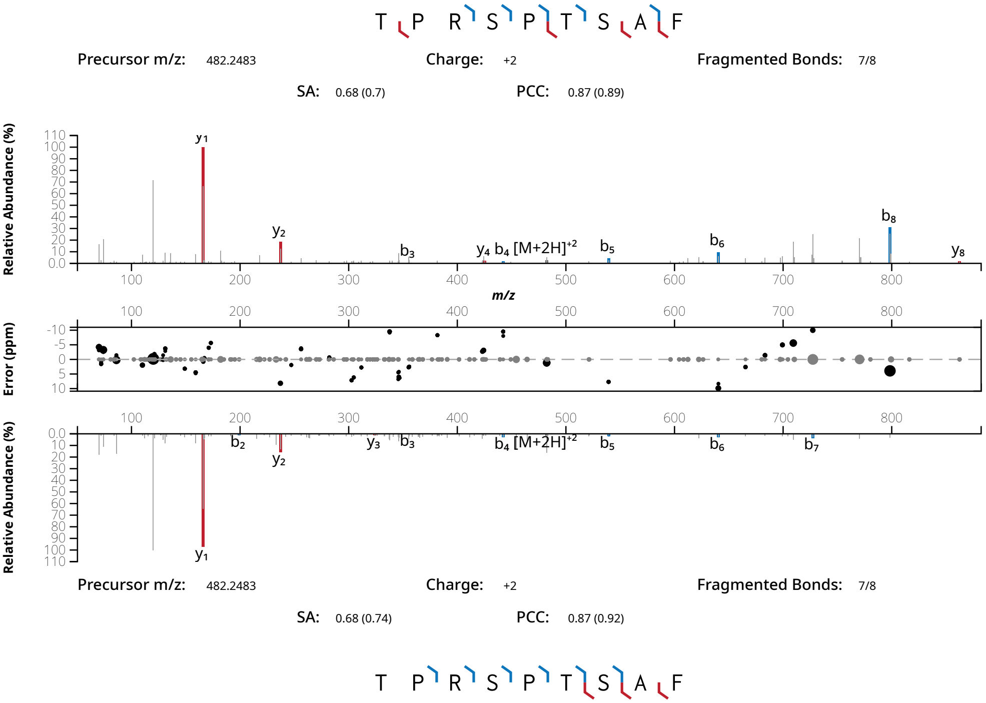
T P R S P T S A F
Precursor m/z: 482.2483
Charge: +2
Fragmented Bonds: 7/8
SA: 0.68 (0.7)
PCC: 0.87 (0.89)
Relative Abundance (%)
y1
y2
b3
y4
b4
[M+2H]+2
b5
b6
b8
y8
m/z
Error (ppm)
Relative Abundance (%)
b2
y2
y3
b3
b4
[M+2H]+2
b5
b6
b7
y1
Precursor m/z: 482.2483
Charge: +2
Fragmented Bonds: 7/8
SA: 0.68 (0.74)
PCC: 0.87 (0.92)
T P R S P T S A F

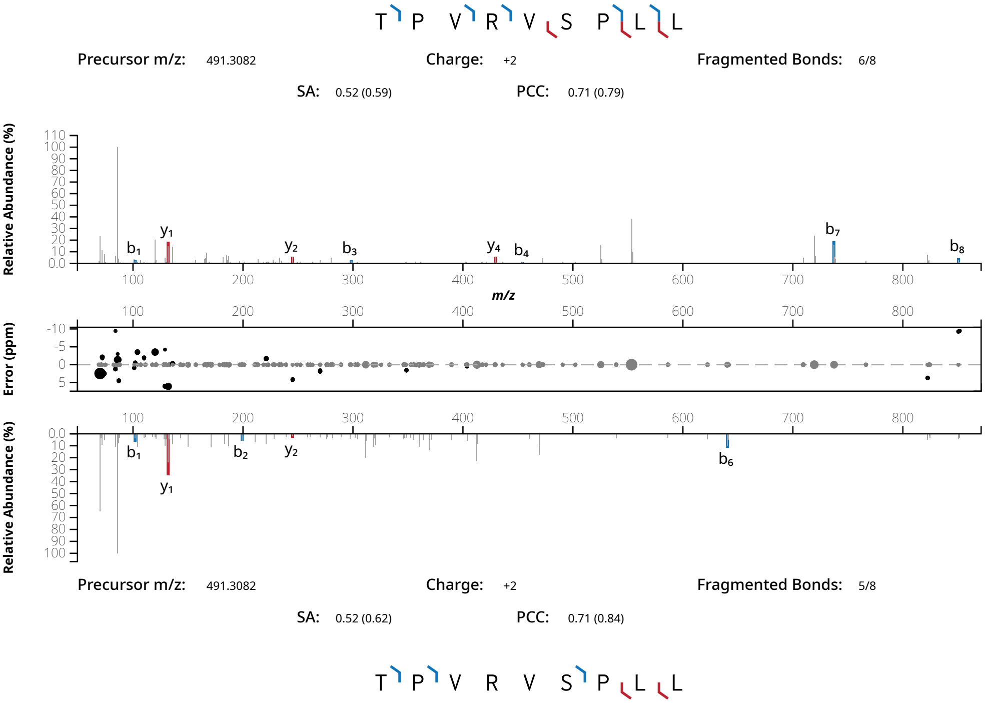
T P V R V S P L L
Precursor m/z: 491.3082
Charge: +2
Fragmented Bonds: 6/8
SA: 0.52 (0.59)
PCC: 0.71 (0.79)
Relative Abundance (%)
m/z
Error (ppm)
Relative Abundance (%)
Precursor m/z: 491.3082
Charge: +2
Fragmented Bonds: 5/8
SA: 0.52 (0.62)
PCC: 0.71 (0.84)
T P V R V S P L L

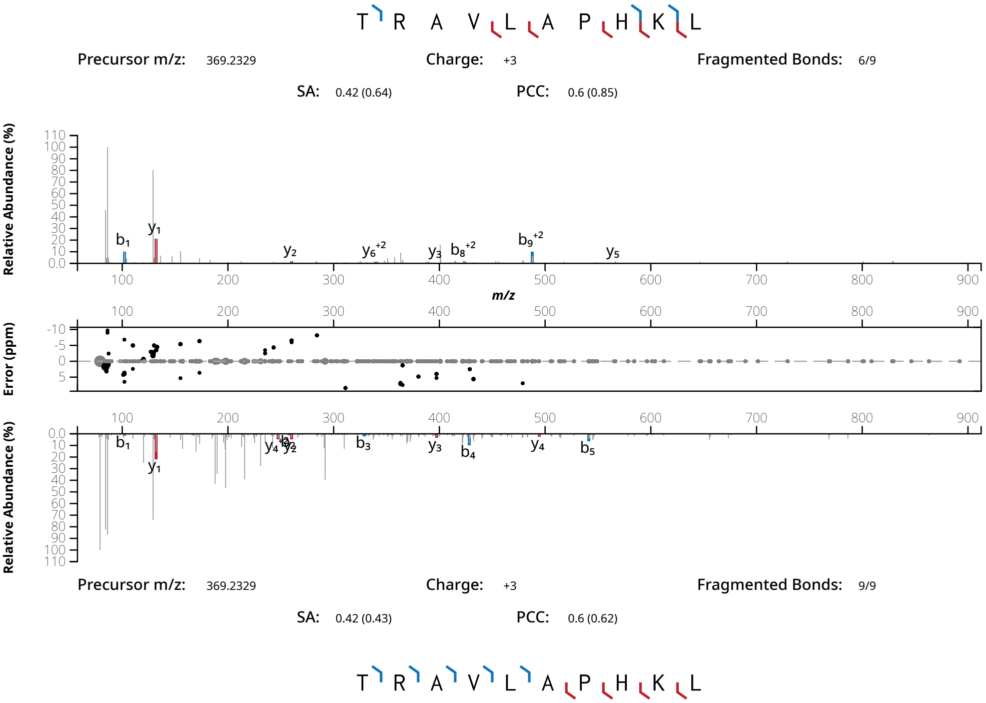
T R A V L A P H K L
Precursor m/z: 369.2329
Charge: +3
Fragmented Bonds: 6/9
SA: 0.42 (0.64)
PCC: 0.6 (0.85)
Relative Abundance (%)
b1 y1 y2 y6+2 y3 b8+2 b9+2 y5
m/z
Error (ppm)
b1 y1 y4+2 y2 b3 y3 b4 y4 b5
Relative Abundance (%)
Precursor m/z: 369.2329
Charge: +3
Fragmented Bonds: 9/9
SA: 0.42 (0.43)
PCC: 0.6 (0.62)
T R A V L A P H K L

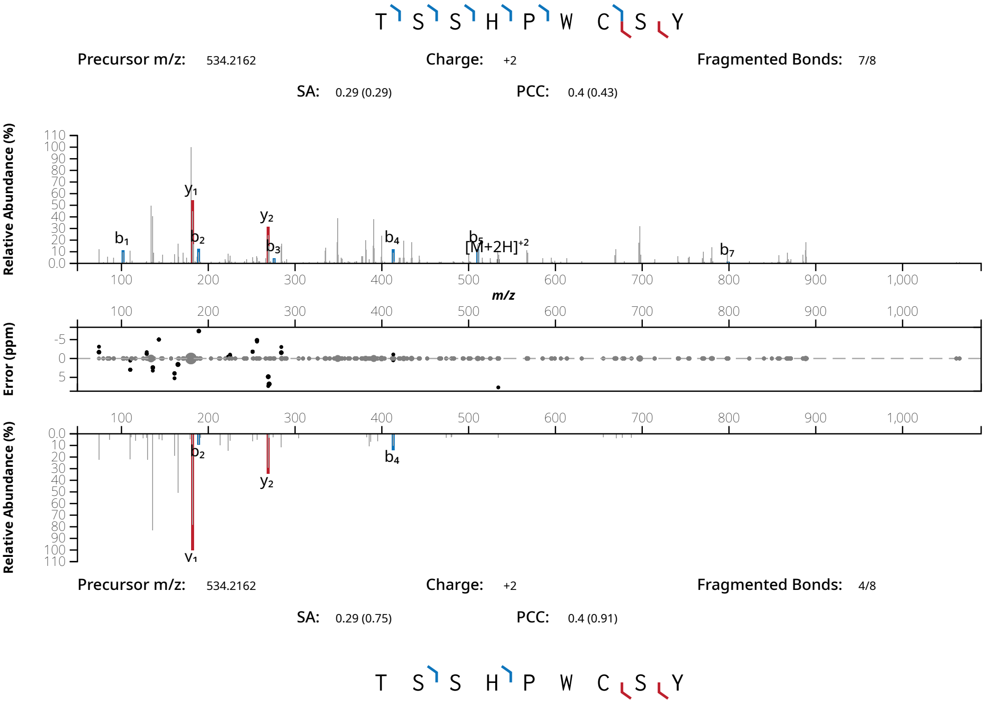
T S S H P W C S Y
Precursor m/z: 534.2162
Charge: +2
Fragmented Bonds: 7/8
SA: 0.29 (0.29)
PCC: 0.4 (0.43)
Relative Abundance (%)
Error (ppm)
m/z
b1 y1 b2 y2 b3 b4 b5 [M+2H]+2 b7
Precursor m/z: 534.2162
Charge: +2
Fragmented Bonds: 4/8
SA: 0.29 (0.75)
PCC: 0.4 (0.91)
T S S H P W C S Y

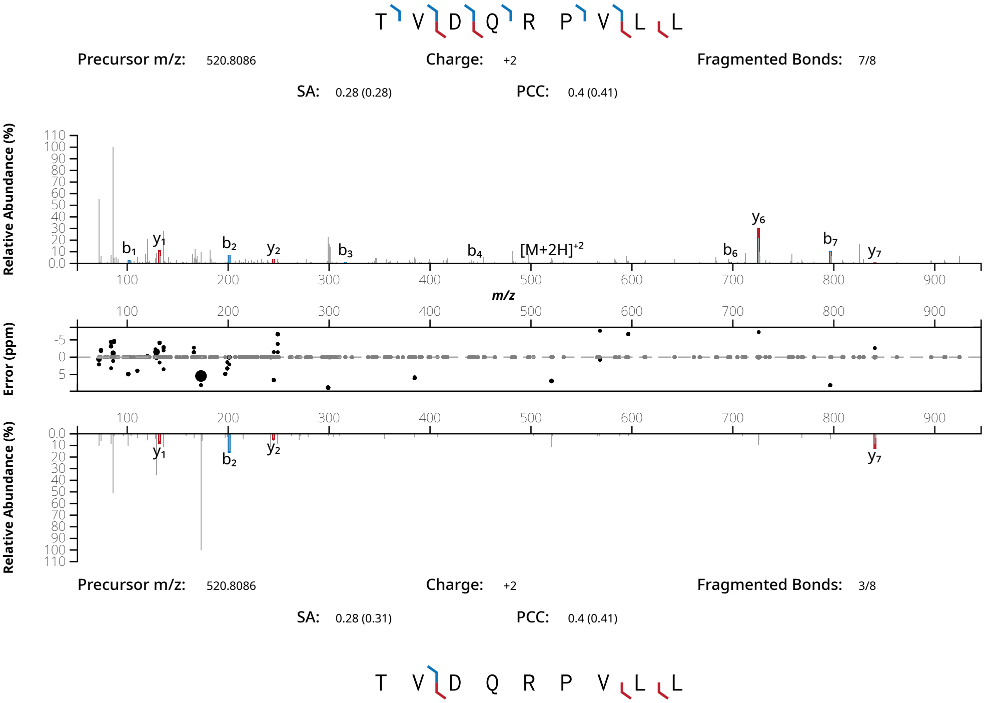
T V D Q R P V L L
Precursor m/z: 520.8086
Charge: +2
Fragmented Bonds: 7/8
SA: 0.28 (0.28)
PCC: 0.4 (0.41)
Relative Abundance (%)
m/z
Error (ppm)
Relative Abundance (%)
Precursor m/z: 520.8086
Charge: +2
Fragmented Bonds: 3/8
SA: 0.28 (0.31)
PCC: 0.4 (0.41)
T V D Q R P V L L

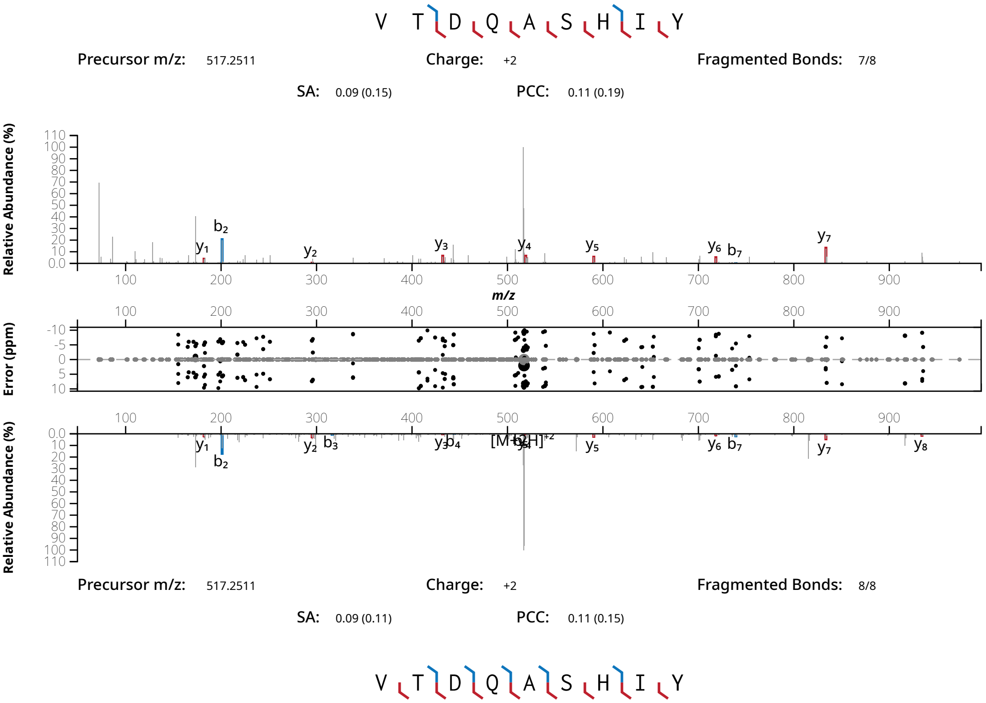
V T D Q A S H I Y
Precursor m/z: 517.2511
Charge: +2
Fragmented Bonds: 7/8
SA: 0.09 (0.15)
PCC: 0.11 (0.19)
Relative Abundance (%)
y1
b2
y2
y3
y4
y5
y6
b7
y7
m/z
Error (ppm)
y1
b2
y2
b3
y3
b4
[M+2H]+2
y4
y5
y6
b7
y7
y8
Relative Abundance (%)
Precursor m/z: 517.2511
Charge: +2
Fragmented Bonds: 8/8
SA: 0.09 (0.11)
PCC: 0.11 (0.15)
V T D Q A S H I Y

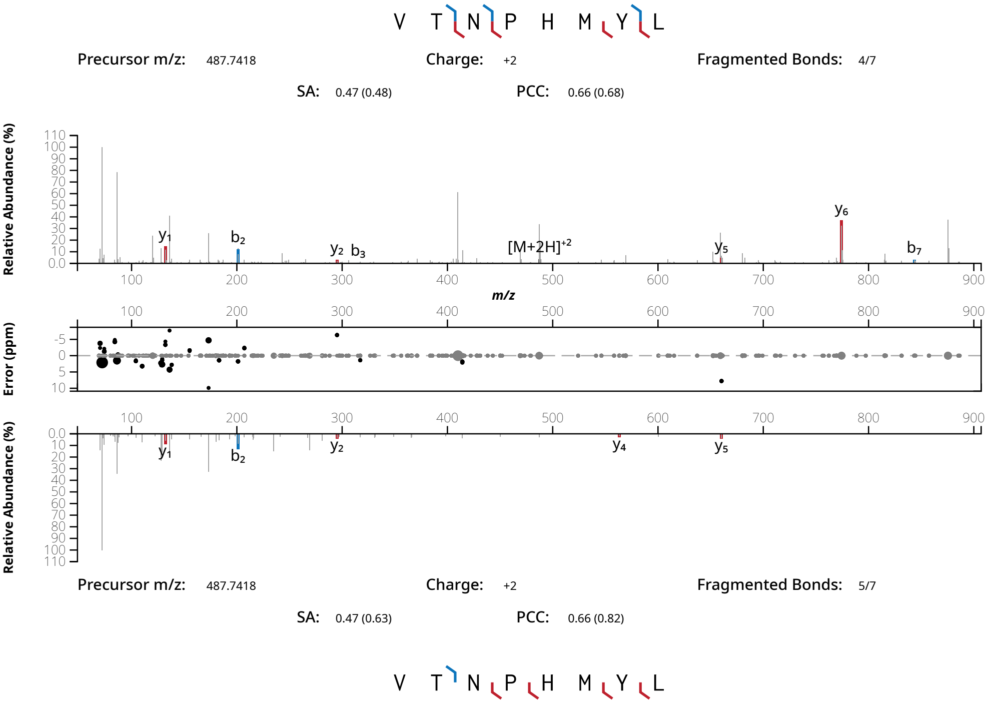
V T N P H M Y L
Precursor m/z: 487.7418
Charge: +2
Fragmented Bonds: 4/7
SA: 0.47 (0.48)
PCC: 0.66 (0.68)
Relative Abundance (%)
y1
b2
y2
b3
[M+2H]+2
y5
y6
b7
m/z
Error (ppm)
Relative Abundance (%)
y1
b2
y2
y4
y5
Precursor m/z: 487.7418
Charge: +2
Fragmented Bonds: 5/7
SA: 0.47 (0.63)
PCC: 0.66 (0.82)
V T N P H M Y L

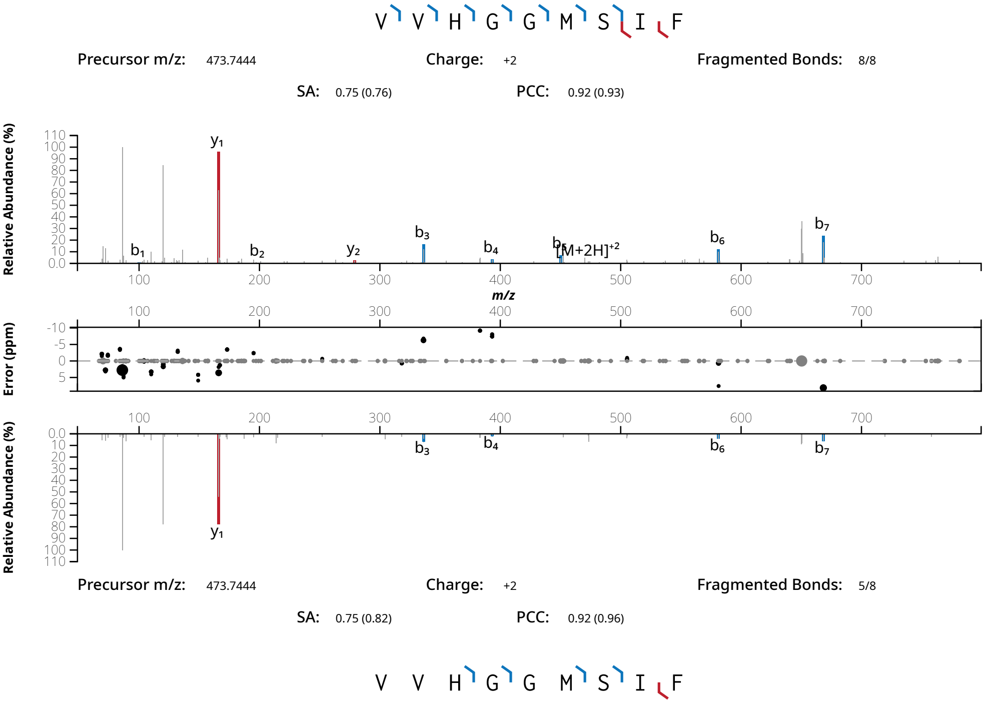
V V H G G M S I F
Precursor m/z: 473.7444
Charge: +2
Fragmented Bonds: 8/8
SA: 0.75 (0.76)
PCC: 0.92 (0.93)
Relative Abundance (%)
y1
b1
b2
y2
b3
b4
b5
[M+2H]+2
b6
b7
m/z
Error (ppm)
Relative Abundance (%)
Precursor m/z: 473.7444
Charge: +2
Fragmented Bonds: 5/8
SA: 0.75 (0.82)
PCC: 0.92 (0.96)
V V H G G M S I F

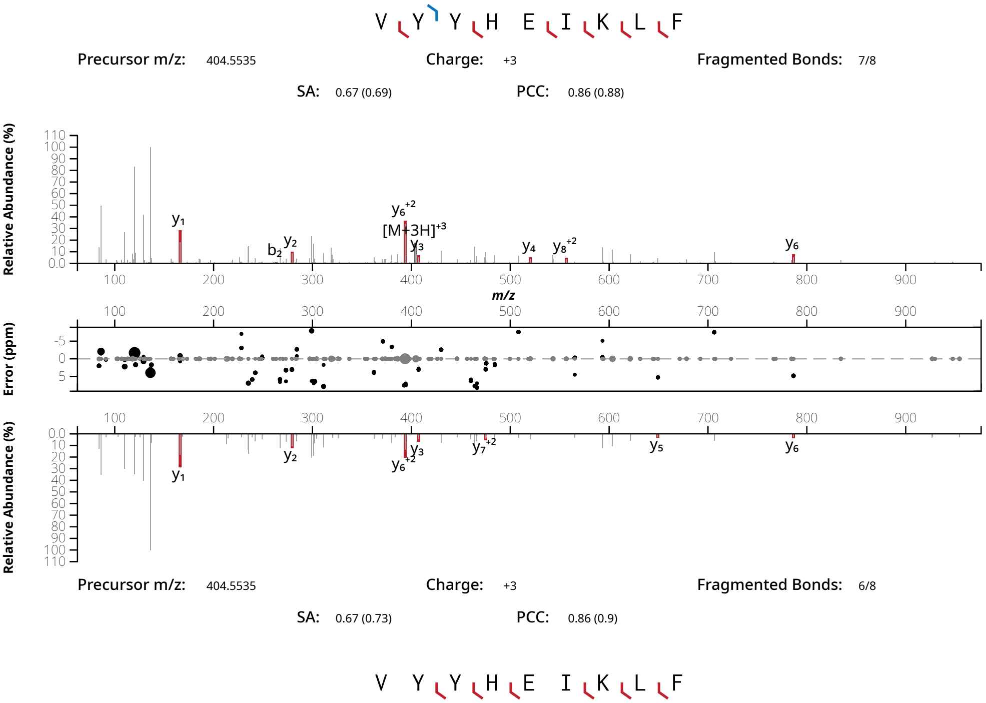
V Y Y H E I K L F
Precursor m/z: 404.5535
Charge: +3
Fragmented Bonds: 7/8
SA: 0.67 (0.69)
PCC: 0.86 (0.88)
Relative Abundance (%)
m/z
Error (ppm)
Relative Abundance (%)
y1
b2
y2
y6+2
[M+3H]+3
y3
y4
y8+2
y6
y7+2
y5
Precursor m/z: 404.5535
Charge: +3
Fragmented Bonds: 6/8
SA: 0.67 (0.73)
PCC: 0.86 (0.9)
V Y Y H E I K L F

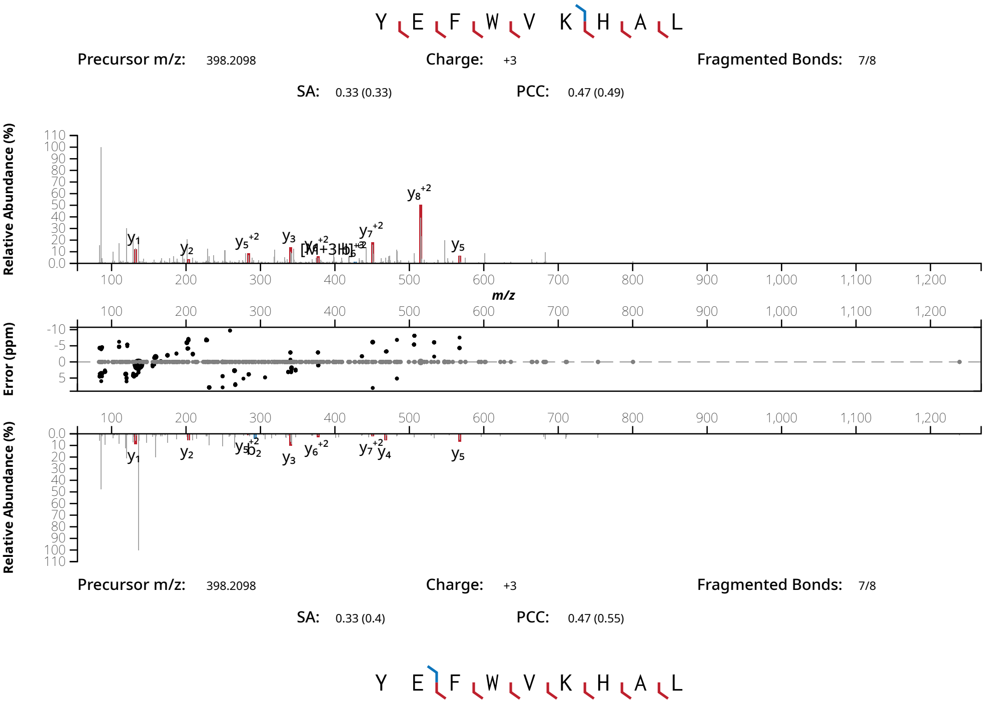
Y E F W V K H A L
Precursor m/z: 398.2098
Charge: +3
Fragmented Bonds: 7/8
SA: 0.33 (0.33)
PCC: 0.47 (0.49)
Relative Abundance (%)
m/z
Error (ppm)
Relative Abundance (%)
Precursor m/z: 398.2098
Charge: +3
Fragmented Bonds: 7/8
SA: 0.33 (0.4)
PCC: 0.47 (0.55)
Y E F W V K H A L

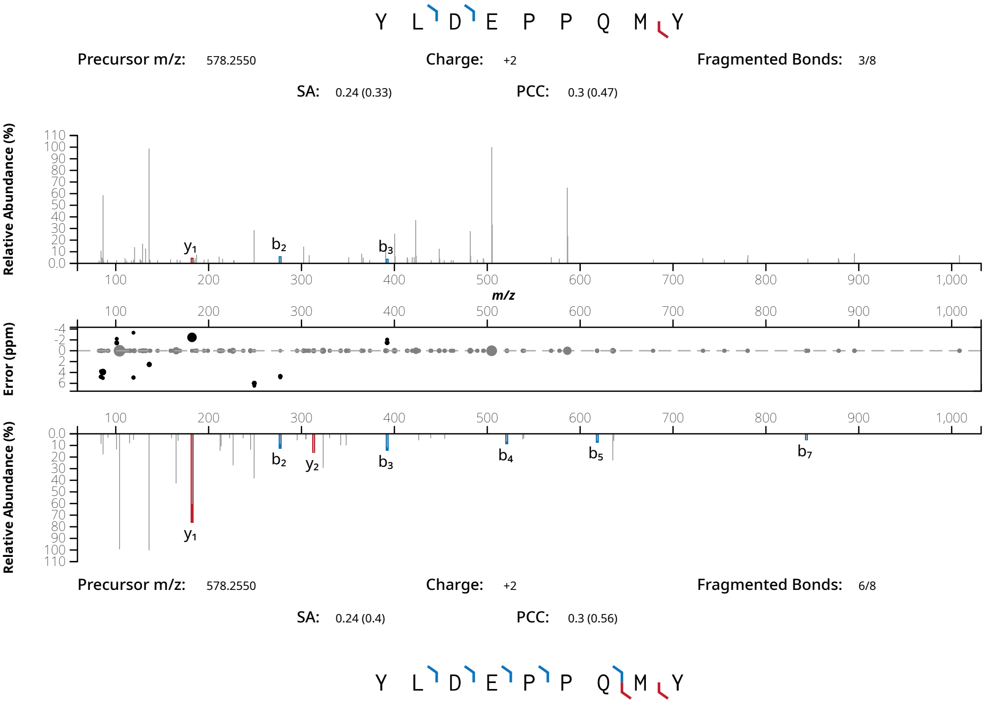
Y L D E P P Q M Y
Precursor m/z: 578.2550
Charge: +2
Fragmented Bonds: 3/8
SA: 0.24 (0.33)
PCC: 0.3 (0.47)
Relative Abundance (%)
m/z
Error (ppm)
y1
b2
b3
y2
b4
b5
b7
Precursor m/z: 578.2550
Charge: +2
Fragmented Bonds: 6/8
SA: 0.24 (0.4)
PCC: 0.3 (0.56)
Y L D E P P Q M Y

Y S D S I S S Y

Precursor m/z: 921.3836 Charge: +1 Fragmented Bonds: 0/7

SA: 0.47 (0.51) PCC: 0.62 (0.68)

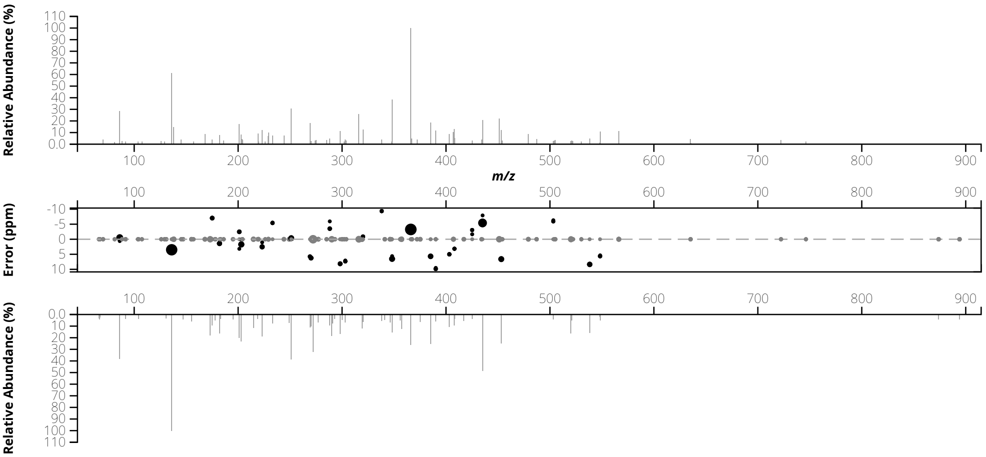

Precursor m/z: 921.3836 Charge: +1 Fragmented Bonds: 0/7

SA: 0.47 (0.51) PCC: 0.62 (0.67)

Y S D S I S S Y
