# Supplementary material for: MARS an improved de novo peptide candidate selection method for non-canonical antigen target discovery in cancer
Source: Nat Commun. 2024 Jan 22;15:661. doi: 10.1038/s41467-023-44460-z (PMC10803737; doi:10.1038/s41467-023-44460-z)
Supplement: Supplementary file 6 — Reporting Summary [file 41467_2023_44460_MOESM6_ESM.pdf]

nature portfolio

Corresponding author(s): Nicola Ternette

Last updated by author(s): Oct 16, 2023

Reporting Summary

Nature Portfolio wishes to improve the reproducibility of the work that we publish. This form provides structure for consistency and transparency in reporting. For further information on Nature Portfolio policies, see our Editorial Policies and the Editorial Policy Checklist.

Statistics

For all statistical analyses, confirm that the following items are present in the figure legend, table legend, main text, or Methods section.

n/a	Confirmed	
☐	☒	The exact sample size (*n*) for each experimental group/condition, given as a discrete number and unit of measurement
☐	☒	A statement on whether measurements were taken from distinct samples or whether the same sample was measured repeatedly
☐	☒	The statistical test(s) used AND whether they are one- or two-sided<br>*Only common tests should be described solely by name; describe more complex techniques in the Methods section.*
☐	☒	A description of all covariates tested
☐	☒	A description of any assumptions or corrections, such as tests of normality and adjustment for multiple comparisons
☐	☒	A full description of the statistical parameters including central tendency (e.g. means) or other basic estimates (e.g. regression coefficient) AND variation (e.g. standard deviation) or associated estimates of uncertainty (e.g. confidence intervals)
☐	☒	For null hypothesis testing, the test statistic (e.g. *F*, *t*, *r*) with confidence intervals, effect sizes, degrees of freedom and *P* value noted<br>*Give P values as exact values whenever suitable.*
☒	☐	For Bayesian analysis, information on the choice of priors and Markov chain Monte Carlo settings
☒	☐	For hierarchical and complex designs, identification of the appropriate level for tests and full reporting of outcomes
☒	☐	Estimates of effect sizes (e.g. Cohen's *d*, Pearson's *r*), indicating how they were calculated

Our web collection on statistics for biologists contains articles on many of the points above.

Software and code

Policy information about availability of computer code

Data collection	MARS software integrates HLA binding prediction, peptide retention time prediction, and average local confidence scores culminating in a linear machine learning model that re-scores de dovo peptide sequencing candidates with improved accuracy. The software further maps identified sequences to their respective origin in the human transcriptome and/or genome, using mainly UNIPROT, ENSEMBLE and GENCODE annotations.
Data analysis	The downloaded original experimental datasets were processed with commercial software Peaks (version X, https://www.bioinfor.com/), then processed by adopted/containerized (Singularity container version 3.2 https://docs.sylabs.io/guides/3.2/user-guide/installation.html), versions of previously published, publicly available softwares: NetMHCpan version 4.1 (https://services.healthtech.dtu.dk/service.php?NetMHCpan-4.1) and DeepRTplus (no version number, https://github.com/horsepurve/DeepRTplus), and seqkit version 2.0.0 (https://github.com/shenwei356/seqkit). Main workflow and analysis were composed in R programme language version 4.2.0. Custom Linux shell scripts were also used in submitting cluster computation jobs to SGE maintained by BMRC, University of Oxford (https://www.medsci.ox.ac.uk/divisional-services/support-services-1/bmrc). MSconvert version 3.0 (https://proteowizard.sourceforge.io/) was used for data format conversion. Universal Spectrum Explorer website (https://www.proteomicsdb.org/use/) was used for mass spectrum visualization.

For manuscripts utilizing custom algorithms or software that are central to the research but not yet described in published literature, software must be made available to editors and reviewers. We strongly encourage code deposition in a community repository (e.g. GitHub). See the Nature Portfolio guidelines for submitting code & software for further information.

Data

Policy information about availability of data

All manuscripts must include a data availability statement. This statement should provide the following information, where applicable:

- Accession codes, unique identifiers, or web links for publicly available datasets
- A description of any restrictions on data availability
- For clinical datasets or third party data, please ensure that the statement adheres to our policy

All data are collected from ProteomeXchange (http://www.proteomexchange.org/), a well-known public Proteomics data repository hosting previously published datasets, including the following studies (accessions):
1. https://doi.org/10.1073/pnas.1911622116 (PXD015489).
1. https://doi:10.1002/pmic.201800357 (PXD011723).
2. https://doi.org/10.1038/s41467-021-23713-9 (PXD021013).
3. https://doi.org/10.1038/s41467-020-14968-9 (PXD013649).
Cervical tumour data has been deposited via ProteomeXchange (http://www.proteomexchange.org/) under PXD046182.

Research involving human participants, their data, or biological material

Policy information about studies with human participants or human data. See also policy information about sex, gender (identity/presentation), and sexual orientation and race, ethnicity and racism.

Reporting on sex and gender	Sex and gender were not considered for cell line and publicly available datasets. Cervical tumour samples were sourced from women only.
Reporting on race, ethnicity, or other socially relevant groupings	This information is not available for human samples used in this study.
Population characteristics	This information is not available for human samples used in this study.
Recruitment	Not applicable
Ethics oversight	University Research Ethics Committee (CUREC) of the University of Oxford, Reference R68126/RE001

Note that full information on the approval of the study protocol must also be provided in the manuscript.

Field-specific reporting

Please select the one below that is the best fit for your research. If you are not sure, read the appropriate sections before making your selection.

[x] Life sciences [ ] Behavioural & social sciences [ ] Ecological, evolutionary & environmental sciences

For a reference copy of the document with all sections, see nature.com/documents/nr-reporting-summary-flat.pdf

Life sciences study design

All studies must disclose on these points even when the disclosure is negative.

Sample size	The sample size was defined by the extend of previously published datasets available and chosen for reanalysis.
Data exclusions	No data was excluded.
Replication	This study is based on a bioinformatics tool which is applied to data, and outcomes are identical with each replicate performance.
Randomization	All spectra were considered in our analyses, randomization of data was not required.
Blinding	We did not perform a trial, and blinding is not applicable in the present study.

Reporting for specific materials, systems and methods

We require information from authors about some types of materials, experimental systems and methods used in many studies. Here, indicate whether each material, system or method listed is relevant to your study. If you are not sure if a list item applies to your research, read the appropriate section before selecting a response.

Materials & experimental systems

n/a	Involved in the study
☐	☒ Antibodies
☐	☒ Eukaryotic cell lines
☒	☐ Palaeontology and archaeology
☒	☐ Animals and other organisms
☒	☐ Clinical data
☒	☐ Dual use research of concern
☒	☐ Plants

Methods

n/a	Involved in the study
☒	☐ ChIP-seq
☒	☐ Flow cytometry
☒	☐ MRI-based neuroimaging

Antibodies

Antibodies used

Pan HLA antibody clone W6/32 (ATCC HB95)

Validation

We are using this antibody for a decade to isolate HLA class I complexes. The specificity of the antibody has been validated multiple times by proteomic analyses of the immunoprecipitated material.

The original work characterising the antibody specificity is referenced here:

Brodsky FM, Parham P. Monomorphic anti-HLA-A,B,C monoclonal antibodies detecting molecular subunits and combinatorial determinants. J. Immunol. 128: 129-135, 1982. PubMed: 6172474

Barnstable CJ, et al. Production of monoclonal antibodies to group A erythrocytes, HLA and other human cell surface antigens -- new tools for genetic analysis. Cell 14: 9-20, 1978. PubMed: 667938

Parham P, et al. Use of a monoclonal antibody (W6/32) in structural studies of HLA- A,B,C, antigens. J. Immunol. 123: 342-349, 1979. PubMed: 87477

Additional experimental evidence for the specificity if this clone is detailed here:

https://www.abcam.com/products/primary-antibodies/hla-class-i-antibody-w632-low-endotoxin-azide-free-ab23755.html

Eukaryotic cell lines

Policy information about cell lines and Sex and Gender in Research

Cell line source(s)

Cell line 721.221 is a female B cell line derived from PBMC immortalized with EBV. The Jurkat cell line is a male T cell acute lymphoblastic leukemia cancer cell line. We used publicly available data for these cell lines.

Authentication

cell lines were not authenticated

Mycoplasma contamination

Mycoplasma testing has been performed and was negative.

Commonly misidentified lines (See ICLAC register)

no misidentified cell lines have been used

Plants

Seed stocks

Report on the source of all seed stocks or other plant material used. If applicable, state the seed stock centre and catalogue number. If plant specimens were collected from the field, describe the collection location, date and sampling procedures.

Novel plant genotypes

Describe the methods by which all novel plant genotypes were produced. This includes those generated by transgenic approaches, gene editing, chemical/radiation-based mutagenesis and hybridization. For transgenic lines, describe the transformation method, the number of independent lines analyzed and the generation upon which experiments were performed. For gene-edited lines, describe the editor used, the endogenous sequence targeted for editing, the targeting guide RNA sequence (if applicable) and how the editor was applied.

Authentication

Describe any authentication procedures for each seed stock used or novel genotype generated. Describe any experiments used to assess the effect of a mutation and, where applicable, how potential secondary effects (e.g. second site T-DNA insertions, mosiacism, off-target gene editing) were examined.
